# Helix–strand interaction regulates stability and aggregation of the human mitochondrial membrane protein channel VDAC3

**DOI:** 10.1085/jgp.201812272

**Published:** 2019-01-23

**Authors:** Ankit Gupta, Radhakrishnan Mahalakshmi

**Affiliations:** Molecular Biophysics Laboratory, Department of Biological Sciences, Indian Institute of Science Education and Research, Bhopal, India

## Abstract

Voltage-dependent anion channels (VDACs) are β-sheet–rich transmembrane β-barrels that are vital for metabolite transport across the mitochondrial membrane. Under cellular stress, human VDACs hetero-oligomerize and coaggregate with proteins that can form amyloidogenic and neurodegenerative deposits, implicating a role for VDACs in proteotoxicity. However, whether VDACs possess intrinsic interaction sites that can lead to protein aggregation is not known. Here, we couple a systematic thiol replacement strategy with far-UV circular dichroism spectropolarimetry and UV scattering spectroscopy to map aggregation-prone regions of human VDACs, using isoform 3 as our model VDAC. We show that the region comprising strands β7–β9 is highly aggregation prone. Further, we find that an α1–β7–β9 interaction (involving the hVDAC3 N-terminal α1 helix) can lower protein aggregation, whereas perturbations of this interaction promote VDAC aggregation. We also show that hVDAC3 aggregation proceeds via a partially unfolded structure. Our findings allow us to propose a plausible mechanism for the role of human VDACs in forming proteotoxic aggregates in the cell. The key target sites on VDACs—strands β7–β9—may be useful for developing VDAC aggregation inhibitors.

## Abstract (precis)

Human mitochondrial VDACs bind amyloidogenic proteins, but do not intrinsically aggregate. Gupta and Mahalakshmi find that an interaction between the N-terminal α-helix and strands β7–β9 regulates VDAC aggregation and stability, providing a plausible mechanism for VDAC coaggregation in cells.

## Introduction

Neurodegenerative diseases such as Alzheimer’s disease (AD) and Parkinson’s disease (PD) involve the progressive and destructive association of proteins intracellularly as plaques (Gorbenko and Trusova, 2011; Betaneli et al., 2012; Boulbrima et al., 2016; Iadanza et al., 2018). With increasing incidence of proteotoxic disorders worldwide, a cure for AD, PD, aging, and other forms of neurodegeneration is extremely vital. A suggested cure is to inhibit protein aggregation (Cuadrado-Tejedor et al., 2011; Smilansky et al., 2015; Boulbrima et al., 2016; Magrì et al., 2016; Saletti et al., 2018). Several proteins can form neurodegenerative aggregates (Iadanza et al., 2018). One of the key membrane proteins that exhibits strong binding affinity with amyloidogenic proteins is the voltage-dependent anion channel (VDAC). VDACs interact with Aβ peptide and form amyloid aggregates (Smilansky et al., 2015; Boulbrima et al., 2016). VDACs are known binding partners for α-synuclein, tau, and superoxide dismutase (SOD); the latter are implicated in PD, AD, and other neurodegenerative disorders (Cuadrado-Tejedor et al., 2011; Rostovtseva et al., 2015; Smilansky et al., 2015; Magri and Messina, 2017; Sorrentino et al., 2017; Iadanza et al., 2018). Owing to the interaction sites available on VDACs for Aβ, α-synuclein, tau, and SOD, it is speculated that VDACs can coaggregate in amyloidogenic structures formed intracellularly. As VDACs function as pro- and anti-apoptotic elements and decide the fate of the cell (Naghdi et al., 2015; Hoogerheide et al., 2017; Magrì et al., 2018), VDAC inhibitors are purported to represent broad spectrum therapeutics for various neurodegenerative diseases (Smilansky et al., 2015; Magri and Messina, 2017). Yet, we have no direct information on whether molecular elements of VDAC sequence and structure can lead to proteopathies and neurodegeneration.

In resting cells, VDACs transport metabolites and ions across the outer mitochondrial membrane and maintain cellular homeostasis (Boulbrima et al., 2016; Maurya and Mahalakshmi, 2017). Humans have three near-identical VDAC isoforms, 1, 2, and 3, that homo- and hetero-oligomerize (Betaneli et al., 2012; Boulbrima et al., 2016; Bergdoll et al., 2018). For example, hetero-oligomerization of human VDAC2 with Bak occurs through strands β7–β10 (Naghdi et al., 2015). VDACs establish a robust interaction network in the cell (Caterino et al., 2017; Hoogerheide et al., 2017) and therefore play a central role in regulating essential cellular processes. Indeed, recent findings establish that VDAC-mediated superior mitochondrial homeostasis offsets the effects of Aβ toxicity (Sorrentino et al., 2017). VDAC dysregulation could cause the protein to bind nonspecifically with other proteins or interact with Aβ, α-synuclein, tau, etc. (Table S1), resulting in disease states (Thinnes, 2011; Rostovtseva et al., 2015; Boulbrima et al., 2016; Magri and Messina, 2017). The three human VDACs share >70% sequence identity, making studies on one isoform also largely relevant to the others. While α-synuclein, tau, Aβ, and other proteins are known to bind VDAC1, we do not have clear evidence that these proteins do not bind VDAC2 or VDAC3. Hence, it is important to gain considerable understanding of all three VDAC isoforms in humans. Recent findings have revealed the importance of VDAC2 and VDAC3 cysteines in sensing and responding to mitochondrial oxidative stress by way of cysteine modifications (Guardiani et al., 2016; Reina et al., 2016a,b; Iadanza et al., 2018; Saletti et al., 2018). Furthermore, human VDAC3 (hV3) cysteines are oxidized to S–OH, S–O_2_H, and S–O_3_H when reactive oxygen species accumulate in the cell (Okazaki et al., 2015; De Pinto et al., 2016; Reina et al., 2016a,b; Saletti et al., 2017), causing hV3 to aggregate. Such aggregates can lead to cellular toxicity (Boulbrima et al., 2016). Therefore, we have reason to believe that the two characteristics—aggregation and cysteine oxidation—are connected.

Aggregation hotspots in proteins can be excellent targets for developing inhibitors of protein aggregation (Sievers et al., 2011; Boulbrima et al., 2016). Although VDACs are implicated in various neurodegenerative diseases, direct evidence for the VDAC aggregation sites is not available. In other words, whether the VDAC β-sheet structure possesses intrinsic aggregation loci is not known. These hotspots cannot be predicted with confidence for membrane proteins (Boulbrima et al., 2016), as the latter are rich in hydrophobic residues. Novel approaches to identify aggregation-prone regions of membrane proteins are therefore essential. Scattering and thioflavin T binding measurements are widely used to study amyloidogenic proteins and their aggregation. With this approach, we recently mapped potential VDAC aggregation hotspots using peptide analogues of the full-length protein (Lella and Mahalakshmi, 2018). Hence, we asked if scattering studies could also be used to obtain information on aggregation-prone regions of the folded VDAC β-barrel. Here, we couple far-UV circular dichroism (CD) and scattering measurements with in silico studies of the full-length hV3 to map the potential aggregation sites of human VDACs, using hV3 as our model protein. We show that the vicinity of strands β7–β9 represents a key aggregation zone for hV3. We also find that in the presence of the interaction network established between α1–β7–β9, hV3 aggregation is suppressed. On the basis of our findings, we propose that the α1–β7–β9 interaction can serve as a promising therapeutic target for stabilizing VDACs and lowering their aggregation in the membrane.

## Materials and methods

### Gene cloning and protein expression

The human *VDAC3* gene (without the intron regions) was originally a gift from V. de Pinto (University of Catania, Italy). The final *hVDAC3* gene used in this study corresponds to the mature hV3 protein (NCBI accession no. NP_005653.3). The gene was cloned in pET-21a vector between Nde1 and Xho1 sites. Single Cys mutants (C2A, C8A, and C122A), double cysteine mutants (C2,8A, C2,122A, and C8,122A), triple mutant (C2,8,122A), tetra mutants (C2,8,122,229A and C2,36,65,229A), and Cys-less mutant C0A were generated using site-directed mutagenesis using transfer PCR (Maurya and Mahalakshmi, 2015). The mutations were further confirmed by Sanger DNA sequencing. The plasmid was used to transform *Escherichia coli* C41 cells as reported earlier (Maurya and Mahalakshmi, 2013, 2015). All the proteins were expressed with a C-terminal hexa-His tag as inclusion bodies in these cells by inducing protein expression using 1 mM isopropyl β-d-1-thiogalactopyranoside at 37°C.

### Protein purification

Protein expressed as inclusion bodies was processed using the rapid inclusion body preparation method described earlier (Gupta et al., 2015) with minor modifications. Briefly, the cells were lysed using flash freezing in liquid nitrogen followed by lysozyme treatment and sonication. The membrane fraction was removed using 2% Triton X-100 (here, the wash volume was increased threefold more than the reported protocol), following which residual membranous components were removed with methanol. The desired protein was obtained as a white insoluble inclusion body fraction. The protein pellet was air dried to remove residual methanol and then dissolved in 50 mM carbonate buffer, pH 10.5, containing 8 M urea for further purification. The denatured protein in urea was purified on an anion exchange column. The bound protein on anion exchange matrix was eluted against a gradient of NaCl in 50 mM carbonate buffer, pH 10.5, containing 8 M urea and 1 M NaCl. The protein in its pure form eluted at ∼12% NaCl. Fractions from chromatography corresponding to the pure protein were pooled and further purified using Ni-NTA chromatography. Here, protein fractions were eluted using 100 mM imidazole prepared in 50 mM carbonate buffer, pH 10.5, containing 8.0 M urea and 15 mM β-mercaptoethanol. Samples checked on 12% SDS-PAGE for protein purity were pooled and dialyzed extensively against water to remove imidazole, urea, and residual salt. The precipitated protein was then lyophilized and the protein obtained as a powder was stored at –80°C until further use.

### Protein folding

The protein powder was dissolved in 8 M guanidine hydrochloride (GdnHCl) containing 10 mM 1,4-dithiothreitol (DTT) and 25% lauryldimethylamine oxide (LDAO; *N*,*N*-dimethyldodecan-1-amine oxide), with the help of an ultrasonicator bath for ∼30 min. The temperature of the sonicator bath was maintained at 25°C. This was necessary to ensure that the unfolded protein was in its fully reduced form and nonspecific disulfides that may have formed during purification were reduced. Residual aggregated or undissolved protein particles were removed by high-speed centrifugation at 18,000 *g* at 25°C. The supernatant containing the unfolded stock (250 µM hV3, 10 mM DTT, and 25% LDAO) was then folded by rapid 10-fold dilution into 50 mM phosphate buffer, pH 7.2, containing 100 mM NaCl and 10 mM DTT, using the protocol described previously for the hVDAC2 isoform (Maurya and Mahalakshmi, 2013). The folded stock solution finally contained 25 µM protein, 2.5% LDAO, 10 mM DTT, 100 mM NaCl, and 800 mM GdnHCl in 50 mM phosphate buffer, pH 7.2. The sample was then incubated overnight at 4°C with rotational mixing. Residual aggregated protein particles were removed the next day by high-speed centrifugation at 18,000 *g* at 4°C. This stock was then diluted fivefold in 1× buffer (50 mM phosphate buffer, pH 7.2, containing 100 mM NaCl and excess 0.6% LDAO) to achieve 5 µM protein in 1% LDAO, 2 mM DTT, 100 mM NaCl, and 160 mM GdnHCl. This final 1× folded stock was quantified using *A*_280_, using the respective extinction coefficient for the WT hV3 protein and each cysteine mutant (calculated using the Expasy ProtParam tool; Gasteiger et al., 2005; http://web.expasy.org/protparam/). The final folded protein was used for all the experiments. Details of how the final protein concentrations and detergent–protein ratios (DPRs) were achieved are below.

### Screening of conditions for hV3 aggregation studies

To identify optimal protein and LDAO concentrations required for hV3 aggregation studies, unfolded protein stocks were first prepared in 8 M GdnHCl as described above. 1% LDAO has previously been used to fold hV3 in its bioactive form (Reina et al., 2016a). Based on this, increasing concentrations of hV3 (5 to 25 µM) were folded in a final detergent concentration of 1% LDAO (DPR varied from ∼8,500:1 to ∼1,700:1). Next, with a constant DPR of 8,500:1, the protein and LDAO concentrations were varied from 2.5 to 20 µM and 0.5 to 4%, respectively. The secondary structure content was estimated in all conditions using far-UV CD spectropolarimetry, and thermal denaturation was performed at various temperature ramp rates (0.5°C/min, 1°C/min, 2°C/min, and 5°C/min; details in the following sections). The thermal denaturation coupled scattering experiments were performed using the TMSPC-8 *T*_m_ analysis system (details in the following sections). The profiles were analyzed to derive the *T*_m_, which was compared across all conditions to identify the best protein concentration, LDAO concentration, DPR, and temperature ramp rates, for subsequent spectroscopic measurements. The results obtained are summarized in Fig. 3.

### Disulfide mapping using mass spectrometry

The folded hV3 WT protein was run on 12% SDS-PAGE, and the monomer band was excised into a fresh vial. The gel pieces were then washed with wash solution (50% acetonitrile prepared with 50 mM ammonium bicarbonate) and incubated at 25°C for 15 min with gentle mixing. The washing step was repeated two to three times until the Coomassie stain was no longer visible. After destaining, the gel pieces were dehydrated by treatment with 100% acetonitrile. The gel pieces were then air dried. The dried gel pieces were used for in-gel trypsin digestion using reported protocols (Maurya and Mahalakshmi, 2013). The following modifications were introduced in the complete protocol: (a) samples were not boiled before loading on SDS-PAGE; (b) no iodoacetamide treatment was provided, so that the true state of cysteines in the folded sample could be confirmed; and (c) the reduction and alkylation steps were not performed, to observe the true oxidation state of cysteines in the folded protein.

Peptide mass fingerprints were obtained on an UltrafleXtreme MALDI ToF/ToF instrument (Bruker Daltonik). Peak identification was performed using BioTools v3.2 (Bruker Daltonik) and Mascot (Matrix Science). The search parameters used for peak identification were as follows: Enzyme, Trypsin; Partials, 2; Mass tolerance, 1.0 D; Taxonomy, *Homo sapiens*; Database, SwissProt; Signal-to-noise ratio, 4:1.

### Electrophoretic mobility analysis to measure protein oligomerization

All the hV3 cysteine mutants in their final folded form were analyzed for their electrophoretic mobility on 12% SDS-PAGE. Folded protein samples were diluted 1.25-fold in reducing dye containing 50 mM DTT. Samples were analyzed without or with boiling at 100°C for 3 min. Protein bands were visualized using Coomassie brilliant blue R-250. The total monomer content present in the folded sample was calculated by gel densitometry using Multi Gauge v2.3, as a percentage of the total lane density, after correcting for the contribution of background.

### Secondary structure estimation and thermal denaturation using far-UV CD

All the CD measurements were performed on a J-815 CD spectropolarimeter (JASCO) equipped with a water-cooled Peltier six-cell system. The secondary structure content of folded hV3 was determined using far-UV CD, using reported parameters (Maurya and Mahalakshmi, 2015). Briefly, a far-UV CD spectrum for all the folded samples was recorded from 203 to 260 nm at a scan speed of 100 nm/min, with a data integration time of 1 s and data pitch of 0.5 nm. The data were averaged over three accumulations and blank corrected for contributions from buffer, LDAO, and DTT. The raw ellipticity value at 215 nm was converted to molar ellipticity (*ME*_215_) by adjusting for individual protein concentrations, using the formula reported earlier (Maurya et al., 2013), and plotted for comparison. For all the CD measurements, a 5-µM folded protein in the folding reaction containing 50 mM phosphate buffer, pH 7.2, 2 mM DTT, 100 mM NaCl, and 1% LDAO was maintained. Samples were analyzed electrophoretically before and after all the CD measurements to check for sample integrity (not depicted). All experiments were repeated two to three times using independently prepared folded proteins, to confirm the experimental accuracy and reproducibility.

Protein unfolding was monitored by thermal denaturation, by measuring the change in raw ellipticity at 215 nm (converted subsequently to *ME*_215_, as outlined above). All data were acquired between 4°C to 95°C, at a fixed temperature ramping of 1.0°C/min, as described previously (Maurya and Mahalakshmi, 2013, 2014). The data were normalized and the unfolded fraction (*f*_U_) at each temperature was calculated (Maurya and Mahalakshmi, 2013, 2014). Individual *f*_U_ data at 215 nm was fitted to a two-state unfolding equation for thermal denaturation (Maurya and Mahalakshmi, 2013), and the midpoint of thermal denaturation (*T*_m-ME_) and apparent enthalpy (Δ*H*_ME-app_) of the reaction were obtained (schematic provided in Fig. 5 A and Fig. 8 A). The standard deviation was calculated over a minimum three independent datasets. We further estimated the nucleation point of unfolding (*T*_m-start-ME_), endpoint of the unfolding reaction (*T*_m-end-ME_), and the cooperativity of the unfolding process by estimating Δ*T*_m_ (schematic provided in Fig. 5 A and Fig. 8 A). Here, Δ*T*_m-ME_ = *T*_m-end-ME_ – *T*_m-start-ME_ and reflects how rapid or cooperative the unfolding process is. All the thermal parameters derived from thermal denaturation measurements across the mutants were plotted in the form of histograms and heat maps to allow for a simplified comparison of the various parameters.

### Thermal denaturation and aggregation measurements using UV light scattering experiments

All the scattering experiments were performed using the TMSPC-8 *T*_m_ analysis system available as an accessory to the UV-VIS spectrophotometer 1800 from Shimadzu Scientific Instruments. Here, the auto zero function was used to normalize for voltage fluctuation, for the calibration of the instrument, and to get a stable baseline. Once the calibration was done, the folded protein samples along with the respective blanks (1% LDAO in 50 mM phosphate buffer, 2 mM DTT, 100 mM NaCl, and 160 mM GdnHCl) were heated. The thermal denaturation was performed between temperature ranges of 20°C and 95°C at a fixed ramping rate of 1.0°C/min. Owing to their small size, protein aggregates scatter light at wavelengths of 300–340 nm, based on the size of the particle. We monitored the increase in absorbance at 280, 320, and 340 nm (*A*_280_, *A*_320_, and *A*_340_) to detect the formation of temperature-induced oligomeric or aggregated protein species.

The change in absorbance of the sample was manually corrected for the contribution of buffer, LDAO, and DTT for the whole temperature range, and the thermal denaturation profiles of each protein were obtained. Each profile was then fitted to a sigmoidal function using SigmaPlot v12.0 (Systat Software), to derive the aggregation nucleation point (*T*_m-start-A340_), midpoint (*T*_m-A340_), and endpoint (*T*_m-end-A340_; schematic is provided in Fig. 5 A), for all the mutants. Here, the cooperativity of the oligomerization and aggregation process was calculated as Δ*T*_m-A340_ = *T*_m-end-A340_ – *T*_m-start-A340_ (schematic is provided in Fig. 8 A). The standard deviation was calculated over a minimum three independent datasets. Thermal parameters derived from the scattering measurement were compared globally for all the mutants in the form of histograms and heat maps, as described above for the CD measurements.

### Isothermal unfolding kinetics measurements using far-UV CD

The unfolding and aggregation of hV3 are coupled. Isothermal unfolding/aggregation kinetics were studied by incubating the samples at various preset temperatures between 40°C and 95°C. This temperature range was chosen based on the unfolding/aggregation transition zone exhibited by hV3 in the far-UV CD thermal denaturation measurements described earlier. In the isothermal experiments, we simultaneously recorded (a) change in ellipticity values (*ME*_215_) and (b) dynode voltage (*HT*_215_ measured in volts), at 215 nm.

The change in ellipticity values upon protein unfolding/aggregation was acquired at every 0.1-s interval at 215 nm, with a bandwidth and data pitch of 1.0 nm, and converted to molar ellipticity (*ME*_215_). Here, *ME*_215_ was directly fitted to single exponential rise function to derive the combined rate for the unfolding and aggregation processes, as reported earlier (Maurya and Mahalakshmi, 2014). Note that the *ME*_215_ follows both unfolding and aggregation of hV3, as the protein aggregates we obtain for hV3 are structured. The natural logarithm values of unfolding and aggregation rates were plotted against the temperature they were acquired as 1,000/T (T is the temperature in degrees Kelvin). The data were then fitted to a polynomial function, to derive the activation energy (*E*_act-ME_) in kcal/mol using the equation: slope = *E*_act_/*R*, as described in the schematic in Fig. 6 A (Maurya and Mahalakshmi, 2014). Here *R* is the ideal gas constant (*R* = 1.987 × 10^−3^ kcal K^−1^ mol^−1^).

Similarly, using the *HT*_215_ profiles recorded at 215 nm, aggregation kinetics of hV3 mutants were measured at various preset temperatures between 40°C and 95°C. Here, an increase in HT voltage is independent of protein unfolding and provides information about the transition of hV3 from the folded to the aggregate state. The HT data were analyzed similar to the isothermal unfolding measurement using *ME*_215_. The data were fitted directly to a single exponential rise function to obtain the rate of aggregation at individual temperatures. Activation energy (*E*_act-HT_) was calculated by fitting the natural logarithm of rates plotted against their respective temperatures to a polynomial function, as described in schematic in Fig. 6 A, right panel. All the samples after isothermal unfolding kinetics experiment were checked on 12% SDS-PAGE to assess the oligomeric and aggregated species, and sample integrity (not depicted).

### Isothermal unfolding kinetics experiments using UV light scattering measurements

Similar to *E*_act-ME_ and *E*_act-HT_, the activation energy for protein aggregation from scattering measurements (*E*_act-A340_) was also determined. To measure the aggregation kinetics of hV3, the increase in absorbance at 340 nm (*A*_340_) was monitored at different preset temperatures between 40°C and 95°C. Here, an increase in *A*_340_ indicates that the protein transitions from native to the aggregated species, due to protein denaturation induced by temperature stress. The data were corrected for contributions from buffer, LDAO, and DTT. The individual kinetics traces were then fitted to a single exponential rise function, and the rates thus derived were further used to calculate the activation energy (*E*_act-A340_) of aggregation. Note that we have ignored the lag phase of all the kinetic traces for the analysis, and only exponential phase has been fitted to estimate *k*_U_. The activation energy values calculated from ellipticity (*E*_act-ME_) and dynode voltage (*E*_act-HT_) were compared with the *E*_act_ derived from *A*_340_ (*E*_act-A340_) to obtain a global mechanism for hV3 unfolding and aggregation.

### Aggregation index calculation using UV absorbance spectroscopy

For aggregation index (*AI*) calculation, folded samples were first heated at 94°C to 95°C for 10 min for aggregates to be formed. The entire sample (containing monomers, oligomers, and aggregates) was cooled immediately to room temperature and quantified directly by measuring the absorbance at 280, 320, and 340 nm (*A*_280_, *A*_320_, and *A*_340_, respectively). The respective extinction coefficient for each mutant was used to determine their concentrations. All three absorbance values (*A*_280_, *A*_320_ and *A*_340_) were then used to calculate the aggregation index for the different samples using the formulas

$$AI_{340}=100\times \left[A_{340}/\left(A_{280}-A_{340}\right)\right],$$

$$AI_{320}=100\times \left[A_{320}/\left(A_{280}-A_{320}\right)\right].$$

*AI* was also monitored as a function of temperature using the same procedure. Each sample was incubated at the defined temperature (between 60°C and 94°C) for 10 min. The *AI*_320_ and *AI*_340_ thus measured at different temperatures were plotted against the respective temperatures to determine the dependence of protein aggregation on the temperature. Samples after *AI* calculation were loaded on 12% SDS-PAGE gels to confirm sample integrity (not depicted).

### Global analysis of thermal parameters

The thermal parameters described in Table 1 were derived as explained in the sections above. Data from two to three independent experiments were analyzed to derive the mean and SD; the values were then used for global analysis. The thermal parameters were divided into distinct subsets based on whether they define protein stability, protein aggregation, or both. Far-UV CD wavelength scans, *T*_m-start_, *T*_m_, *T*_m-end_, and *E*_act_ derived from *ME*_215_ were used to measure hV3 secondary structure and protein stability. *T*_m-start_, *T*_m_, and *T*_m-end_ monitored using *A*_340_, as well as *E*_act_ monitored using *HT*_215_ and *A*_340_, were used to measure aggregation propensity. *AI*_320_ and *AI*_340_ measured using UV scattering were also used to measure aggregation propensity. Δ*H*_ME215_ and Δ*T*_m_ were used to monitor the rate of unfolding or aggregation. Analysis of each thermal parameter was performed across the mutants, as follows: (a) For each thermal parameter, the dataset was normalized independently between 0 and 1 to identify the least and most stable variant, respectively. (b) Thermal parameters being compared were placed together and the mutants were sorted globally from lowest to highest values. (c) The data were color coded between shades of red, yellow, and green to generate the heat maps. Heat maps were used to analyze the stability and aggregation tendency for all the proteins globally. (d) The most and least stable mutants obtained by comparing similar thermal parameters (point b) were used for interpretation of the results. (e) Minor variations in thermal parameters were ignored in the analysis, and we compared only the major changes that were significant. In this process, meaningful conclusions could be reached.

**Table 1.** Summary of measured parameters for hV3.

Parameter measured		Protein characteristic or process that is monitored
*ME*	*ME*_215_	Secondary structure content
*T*_m_	*ME*_215_	Stability of folded state
	*A*_340_	Stability of folded monomer; aggregation tendency
*T*_m-start_	*ME*_215_	Stability, temperature at which protein unfolding is nucleated
	*A*_340_	Stability, temperature at which protein aggregation is nucleated
*T*_m-end_	*ME*_215_	Stability, end-point temperature of protein unfolding
	*A*_340_	Stability, end-point temperature of protein aggregation
Δ*T*_m_	*ME*_215_	Indirect measure of rate of unfolding and aggregation
	*A*_340_	Indirect measure of rate of aggregation
Δ*H*_ME-app_	*ME*_215_	Apparent unfolding enthalpy; indirect measure of unfolding cooperativity
*E*_act_	*ME*_215_	Stability, activation energy of unfolding and aggregation
	*A*_340_	Stability, activation energy of aggregation
	*HT*_215_	Stability, activation energy of aggregation
*AI*	*A*_320_	Aggregation propensity
	*A*_340_	Aggregation propensity

*ME*, molar ellipticity measured using CD spectropolarimetry; *A*_340_ or *A*_320_, absorbance measured using UV absorbance spectroscopy at 340 nm or 320 nm, respectively.

All mutants and all datasets were used for the global analysis. The major differences in thermal parameters (high significance) were used for deducing the overall conclusions. The complete data are presented in the supplemental information.

### Characterization of β-aggregates using thioflavin T (ThT) fluorescence

Aggregated protein samples obtained after isothermal unfolding were used for ThT assay. Here, the aggregated protein sample was first diluted ∼3.5-fold with 0.5% LDAO (prepared in 50 mM sodium phosphate buffer, pH 7.2, containing 100 mM NaCl), and a final ThT concentration of 10.0 µM was used. After mixing the dye, all the samples were incubated at 25°C for 15 min, with constant mixing to prevent settling of the protein aggregates. Fluorescence emission scans were recorded at a fixed excitation wavelength of 430 nm on a FluoroMax4 spectrofluorometer (Horiba Jobin-Yvon). Emission spectra were acquired between 460 and 550 nm at 1-nm increments. A fixed slit width of 5 nm for both excitation and emission was used. The emission intensity and wavelength of maximum of emission were monitored for all protein samples. Here, the fluorescence emission intensity and the wavelength of emission maximum of ThT dye changes according to whether ThT is bound to the folded, unfolded, or aggregated state of the protein. ThT fluorescence from unfolded (in 8 M GdnHCl), folded (in 1% LDAO), and aggregated (obtained directly by resuspending the lyophilized protein powder in buffer) proteins samples were also recorded as negative and positive controls, respectively. All the data were corrected for contributions from ThT, buffer, LDAO, GdnHCl, and DTT.

### Characterization of VDAC aggregates using scanning electron microscopy (SEM)

The hV3 protein powder was resuspended in 20 mM Tris-HCl buffer, pH 8.5, and drop-coated onto carbon tapes placed on brass stubs. The sample was allowed to air dry overnight at 25°C. The samples were further dried under a nitrogen stream for 10 min and kept in a desiccator. Then a gold film was deposited for 120 s on the dried sample, using a Q150R rotary-pumped sputter coater instrument (Quorum Technology). The gold-coated samples were then imaged using a high-resolution field emission scanning electron microscope (Zeiss UltraPlus FE-SEM) at an accelerating voltage of 10 kV.

### Prediction of aggregation-prone zones using in silico methods

In silico prediction was executed using the protein sequences of hVDAC1, hVDAC2, and hVDAC3 (hV1, hV2, hV3). All three sequences were obtained from the NCBI database (hVDAC1: NP_003365.1; hVDAC2: NP_003366.2; hVDAC3: NP_005653.3). We used the following algorithms: Tango, Waltz, Aggrescan, FISH Amyloid, Zyggregator, FoldAmyloid, PASTA 2.0, AMYL-PRED, AMYL-PRED 2, GAP, PaFig, AmyloidMutants, Amyloidogenic Pattern, MetAmyl, Average Packing Density, β-Strand Contiguity, Hexapeptide Conformational Energy, NetCSSP, Possible secondary structure Conformational Switches, and CamSol for prediction of amyloidogenic regions in all three VDAC polypeptide sequences. Details are in the Supplemental notes. Note that all the algorithms are used to predict amyloidogenic regions, and not merely the aggregation-prone regions. Here, the raw protein sequence was used as input in each tool; all the tools were run with default settings, except PASTA 2.0, where the threshold was fixed at 90% for prediction of amyloidogenic segments to increase the confidence in the predicted values. Scores were assigned to each residue (0, not amyloidogenic; 1, amyloidogenic) for results obtained from each prediction tool. Then, the aggregation propensity of the whole sequence was compared across the 20 prediction tools by the sum of scores. The consensus residues predicted from a minimum of four methods (aggregation tendency score of ≥20%) was derived and presented as a histogram. These segments were also mapped on the modeled structures of hV1, hV2, and hV3.

### Online supplemental information

The Supplemental notes describe (a) primary and secondary aggregation site of hV3 and (b) additional details of in silico methods. Table S1 explains the involvement of hVDACs in neurodegenerative diseases. Fig. S1 compares the folding and stability of hV3 in different DPRs, and the oligomerization tendency of various hV3 variants. Fig. S2 is the extension of Fig. 5 and explains the importance of β7–β9 in thermal stability of hV3 variants. Fig. S3 and S4 are the extension of Fig. 6 and show kinetics traces and compare the activation energy of various hV3 variants. Figs. S5 and S6 compare the aggregation propensity of all the hV3 variants and highlight the involvement of β1–β4 in promoting hV3 aggregation. Fig. S7 is the extension of Fig. 7 and compares the thermal parameters from barrel unfolding and aggregation. Fig. S8 is the extension of Fig. 8 and compares the unfolding and aggregation cooperativity of hV3 variants. Fig. S9 shows the binding of native hV3 aggregates to ThT dye. Fig. S10 is the extension of Fig. 8 and Figs. S5, S6, S7, and S8 and shows the global comparison of aggregation propensity of all the hV3 variants. Figs. S11, S12, and S13 compare the predicted aggregation zones across the VDAC isoforms, identified from in silico tools or web servers.

## Results and discussion

### Screening experimental conditions for VDAC folding, unfolding, and aggregation

**Figure 1.**
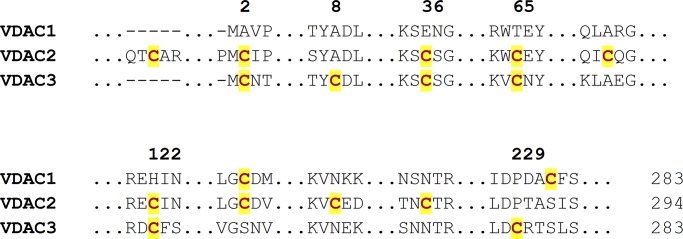
**Cysteines of hVDAC isoforms.** Multiple sequence alignment of the three hVDAC isoforms highlighting cysteines in the sequence (red font, yellow highlight). Cysteines are numbered according to the hV3 sequence.

VDACs are 19-stranded β-barrels with an N-terminal helix α1. In humans, the mitochondrial outer membrane has three VDAC isoforms: 1, 2, and 3. Of the three VDACs, hVDAC2 and hVDAC3 (hV3) are rich in cysteines (Fig. 1). Cysteines respond to mitochondrial oxidative stress through oxidative modifications of their side chain (Guardiani et al., 2016; Reina et al., 2016a,b; Saletti et al., 2017, 2018; Sorrentino et al., 2017; Iadanza et al., 2018). In particular, hV3 is known to oligomerize and aggregate upon oxidation of its cysteines to S–OH, S–O_2_H, and S–O_3_H (Okazaki et al., 2015; De Pinto et al., 2016; Guardiani et al., 2016; Reina et al., 2016a,b; Saletti et al., 2017). Our recent reports (Reina et al., 2016a) also identify the susceptibility of hVDAC3 to aggregation under oxidative stress. Hence, aggregation and cysteine oxidation are linked in hV3. Based on these findings, we probed the relevance of cysteines in hVDAC3 aggregation. Considering the importance of hV3 thiols, we used cysteines as our reporters to identify molecular factors that, at the residue level, stabilize the VDAC scaffold or can trigger barrel oligomerization and aggregation.

**Figure 2.**
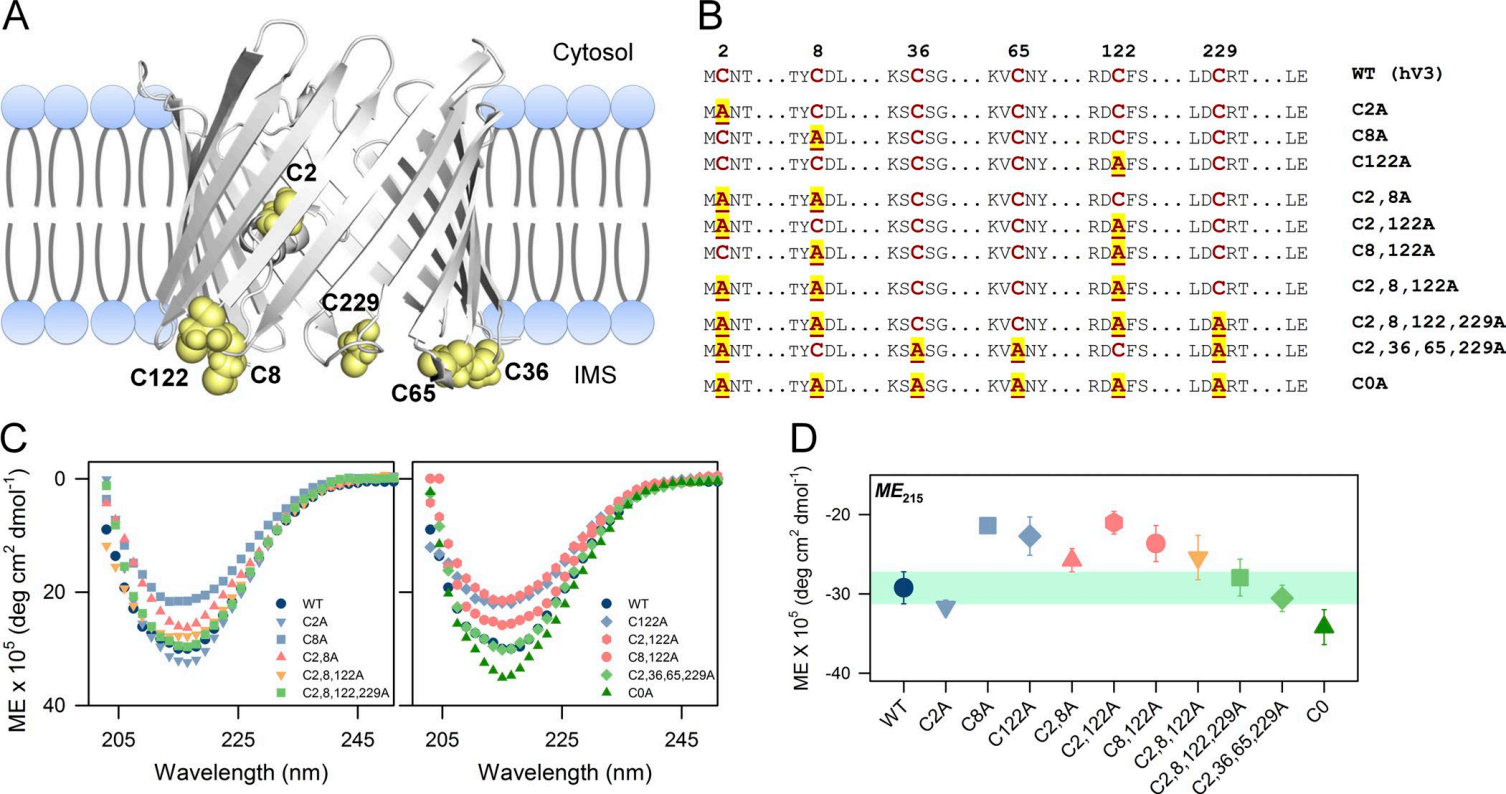
**Structural features of hV3. (A)** Cartoon representation of hV3 β-barrel scaffold, highlighting the distribution of cysteines. IMS, intermembrane space. **(B)** List of hV3 cysteine mutants used in this study. C→A substitutions are highlighted. **(C)** The secondary structure of hV3 mutants derived from far-UV CD. All the proteins exhibit a typical β-sheet structure. **(D)** Comparison of secondary structure content at *ME*_215_ of the various cysteine mutants. Data for WT hV3 is shown as a shaded zone across the plot for comparison. Note how the mutation of C8 and C122 considerably lowers hV3 secondary structure (single Cys mutants C8A, C122A; and double mutants C2,8A, C2,122A, and C8,122A).

hV3 has six cysteines, namely C2, C8, C36, C65, C122, and C229, that are situated toward the intermembrane space (Fig. 2 A). We have previously observed that hVDAC2 cysteines are important for barrel stability and function (Maurya and Mahalakshmi, 2013, 2015). We have also established that channel gating characteristics of LDAO-refolded hV3 are cysteine dependent (Reina et al., 2016a). The abundance of cysteines in hV3 plays an important role in structural stabilization of this barrel and regulates barrel oligomerization and aggregation owing to its chemical nature (Guardiani et al., 2016; Reina et al., 2016a). We created a series of Cys→Ala mutations (Fig. 2 B) and used them as reporters of hV3 stability, unfolding, and aggregation. Cysteines have varied contributions in different proteins and are generally mutated to Ser or Ala. Here, our choice of Ala (over Ser) was based on (a) similar transfer free energy values for Cys and Ala in transmembrane β-barrels (Moon and Fleming, 2011) and (b) similar hydropathy values (Moon and Fleming, 2011). In addition, Cys→Ala substitution offers minimal perturbation in secondary structure.

The hV3 WT and mutants were produced with a C-terminal His_6_ tag as inclusion bodies in *E. coli* and purified (affinity chromatography followed by ion exchange). Preparation of the folded hV3 in concentrations required for spectroscopic measurements was difficult to achieve in phosphocholine lipids, as aggregation is predominant in this condition (i.e., hV3 folding efficiency is poor). LDAO has been used previously for structural and functional studies of various VDACs (Maurya and Mahalakshmi, 2013, 2015; Schredelseker et al., 2014; Okazaki et al., 2015; Rostovtseva et al., 2015; Reina et al., 2016a; Bergdoll et al., 2018), as it supports the β-barrel scaffold of VDACs. We were able to fold hV3 in LDAO, in a range of DPRs from 1,700:1 to 17,000:1 (details of the folding protocol are in Materials and methods). In LDAO micelles, hV3 adopts a β-sheet structure characteristic of VDACs (Fig. 2 C). Additionally, LDAO-refolded hV3 shows channel gating characteristics when incorporated in phosphocholine membranes (Okazaki et al., 2015; Reina et al., 2016a), suggesting that LDAO-refolded hV3 remains functionally active.

**Figure 3.**
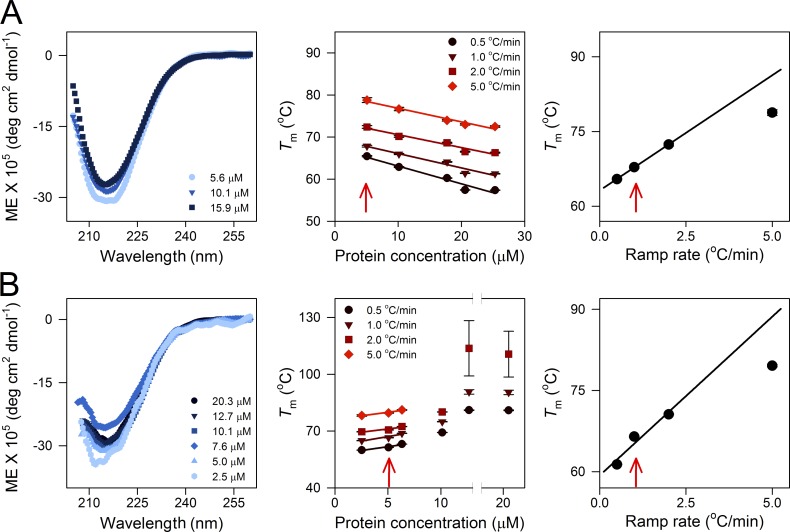
**Screening of folding conditions for hV3 in LDAO micelles.** hV3 folding and stability at various protein and LDAO concentrations were screened by changing the DPR. At DPRs <8,500:1, we obtain a large population of folded dimers and higher-order oligomers of hV3. This population of oligomers varies across the preparations. Further, the thermal parameters we measure vary nonlinearly with increasing hV3 concentrations, thus giving rise to experimental artifacts in the analysis. This is demonstrated using the results from screens performed using the C2,8,122,229A variant, as this mutant is moderately stable and has a high aggregation propensity across the series. **(A)** Effect of protein concentration on structure and thermal stability of hV3 at a constant detergent concentration of 1% LDAO, measured by varying the DPR. Left: The protein exhibits the anticipated β-sheet structure upon folding in LDAO. Here, we observe a considerable decrease in the secondary structure content when the protein concentration is increased (light blue to dark blue). Middle: Dependence of *T*_m_ of aggregation on protein concentration at various temperature ramp rates (dark red to light red). Here, we observe a linear decrease in *T*_m_ upon increasing the hV3 concentration, at all the ramp rates. The observed linear correlation between protein concentration and *T*_m_ suggests that the aggregation can be studied at any of these concentrations without considerable influence from the absolute protein concentration. We have used the lowest protein concentration (red arrow), to minimize the contribution of nonspecific aggregation in our measurements. Right: Correlation between *T*_m_ and temperature ramp rate, at a fixed DPR of 8,500:1. We observe a linear increase in *T*_m_ at lower ramp rates (<2°C/min); at high ramp rates (>2°C/min), we observe nonlinearity between *T*_m_ and temperature ramp rate. The error bars indicate goodness of fit. **(B)** Effect of protein concentration on structure and thermal stability of hV3 at a constant DPR of ∼8,500:1. Here, both the protein and LDAO concentrations have been proportionately varied from 2.5 to 20 µM and 0.5 to 4%, respectively, so as to maintain a constant DPR. Left: Secondary structure content of hV3 at a constant DPR of ∼8,500:1. The folded protein exhibits similar secondary structure in most of the concentrations (light blue to dark blue), except the lowest LDAO concentration of 0.5%. Middle: Dependence of *T*_m_ of aggregation on protein concentration at various temperature ramp rates (dark red to light red). Here, we observe a linear increase in *T*_m_ upon increasing both LDAO and hV3 concentration between 0.5 and 2% LDAO. Above 2% LDAO, we observe nonlinearity in *T*_m_ and protein concentration at all the ramp rates_._ Thus, the LDAO concentration between 0.5 and 2% is sufficient for optimal folding of hV3 and to study protein unfolding and aggregation. We do not observe aggregation >2% LDAO at a temperature ramp rate of 5°C/min; thus, we were not able to derive the *T*_m_ of aggregation at higher LDAO concentrations. Right: Correlation between *T*_m_ of aggregation and temperature ramp rate at a fixed DPR of ∼8,500:1 (1% LDAO and 5 µM hV3). Here again, we observe a linear correlation between *T*_m_ and temperature ramp rate up to 2°C/min; the dependence is nonlinear at faster ramp rates. The error bars indicate goodness of fit. Overall, the data suggest that a moderate protein and detergent concentration can be used to optimally fold hV3 constructs to study protein unfolding and aggregation. We chose a final concentration of 5 µM hV3 in 1% LDAO for our experiments (DPR = 8,500:1), as we obtained a linear dependence of the protein concentration on the *T*_m_ and ramp rate (denoted in all the graphs by a red arrow).

Next, we varied the hV3, LDAO concentrations, and DPRs to screen for conditions that were optimal for hV3 folding, stability, and unfolding/aggregation measurements (Figs. 3 and S1 A). Increasing the protein concentration led to a concomitant loss in β-sheet content. hV3 stability proportionally decreases when the protein concentration increases or DPR decreases (Fig. 3). At high LDAO concentrations (>2% LDAO), hV3 does not unfold within temperatures that can be monitored spectroscopically. At a DPR of ∼8,500:1, where the LDAO concentration is between 0.5 and 2.0%, hV3 is well structured, is stable at 25°C, and shows minimal nonspecific oligomerization or aggregation when folded. Here, the unfolding/aggregation temperature linearly correlated with the protein concentration as well as the temperature ramping rate. In this DPR, we also observed that our hV3 aggregation measurements are least influenced by the absolute protein concentration, and experimental artifacts are not detected (see legend of Fig. 3 for details; also see Fig. S1 A). Hence, we chose a final concentration of 5 µM hV3 in 1% LDAO for our experiments (DPR ≈ 8,500:1; see Materials and methods for more details).

**Figure 4.**
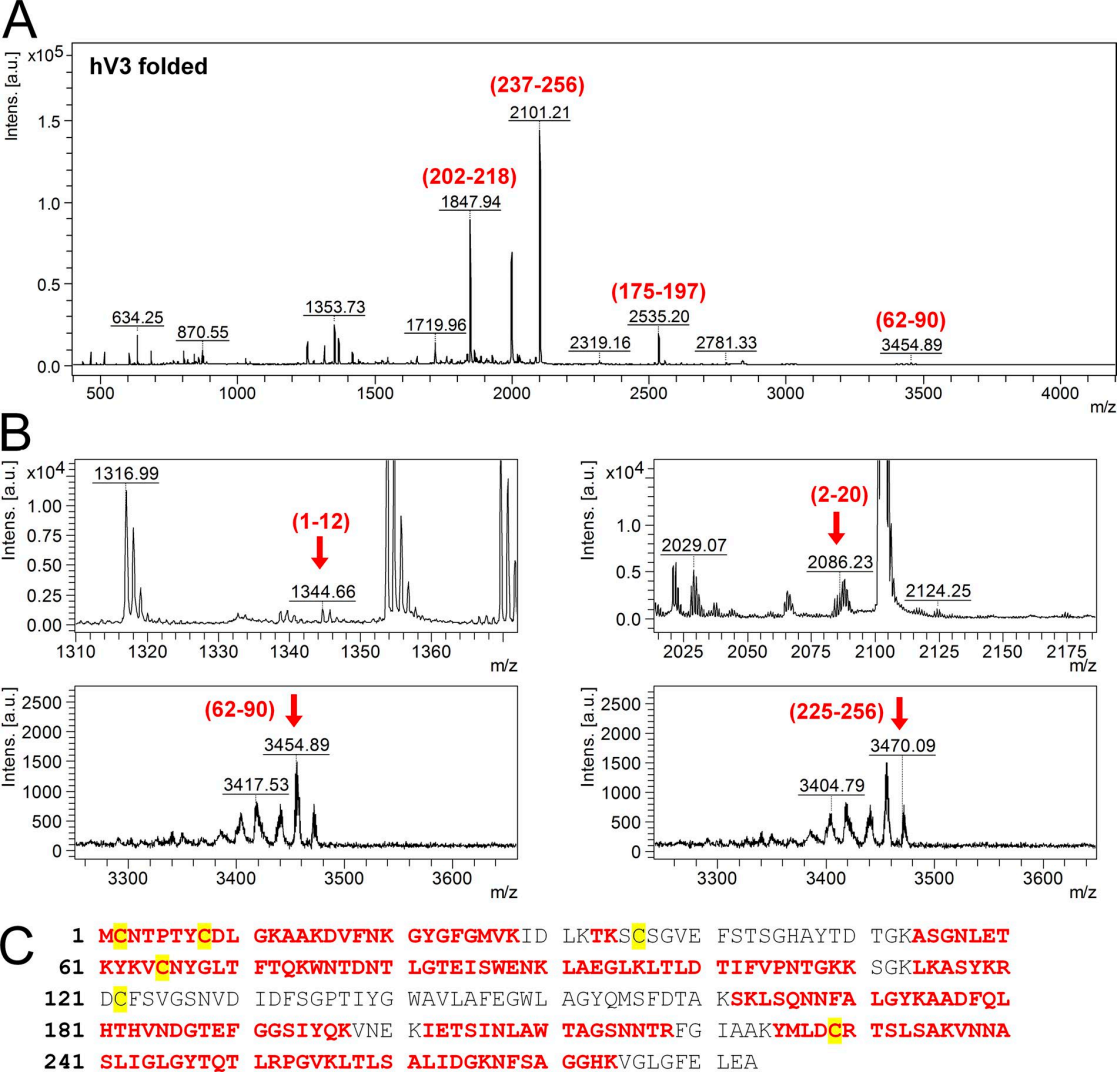
**Mass spectrometric (MS) analysis of folded hV3 to map the oxidation state of cysteines. (A and B)** Representative MS/MS profile of folded hV3 monomer obtained from in-gel trypsin digested samples prepared without iodoacetamide treatment followed by analysis on a MALDI-ToF/ToF mass spectrometer. The regions marked in red (residue numbers are indicated above each peak) are the peaks identified using BioTools v3.2 as corresponding to hV3. Note that hV3-generated peptides show lower ionization efficiency owing to the inherent hydrophobicity of the sequence. Peaks corresponding to the cysteine residues of hV3 have been presented in B and are marked by red arrows. None of the observed peaks could be mapped to a disulfide-bonded species. **(C)** Total coverage of the MS data on the hV3 sequence (marked in red). The cysteines have been highlighted in yellow. Four of the six cysteines were identified from the MS studies. The other cysteines (C36 and C122) are present in large hydrophobic segments. It has not been possible to map these peptides even in prior MS studies of hV3, owing to the extremely hydrophobic nature and poor ionization efficiency of these segments (Saletti et al., 2017). MS fingerprinting using other proteases such as pepsin also did not yield the peptide fragments corresponding to these cysteines (not depicted).

As hV3 WT has six cysteines that are expected to remain in the reduced state under physiological conditions, we checked for intramolecular disulfides using mass spectrometric footprinting. Our results confirm that cysteines remain in their reduced state in folded hV3 WT (Fig. 4). Additionally, we retained DTT in all preparations to prevent disulfide bond formation. In our electrophoretic mobility studies, however, we observed a minor population of oligomeric species that was independent of the cysteine content (Fig. S1, B and C). We believe that these are mutant-specific nondisulfide oligomers.

### Docking of N-helix on barrel scaffold stabilizes hV3

First, we probed the effect of cysteine perturbation on the hV3 secondary structure using far-UV CD. All mutants show β-sheet structure with the anticipated negative exciton maximum at 215 nm (Fig. 2 C). Interestingly, mutating C8 (located in α1) or C122 (located in loop between strands β7 and β8) lowers hV3 secondary structure content, while mutating C2 (in α1) increases hV3 structure (Fig. 2 D). Further, the effect of cysteine mutation is nonadditive, and we did not observe a direct correlation of secondary structure with the total number of cysteines. For example, although the Cys-less mutant C0A is the most structured protein, the difference of secondary structure (*ME*_215_) between C0A and WT is only marginal. Overall, the data suggest a position-specific effect of the mutation on hV3 structure.

**Figure 5.**
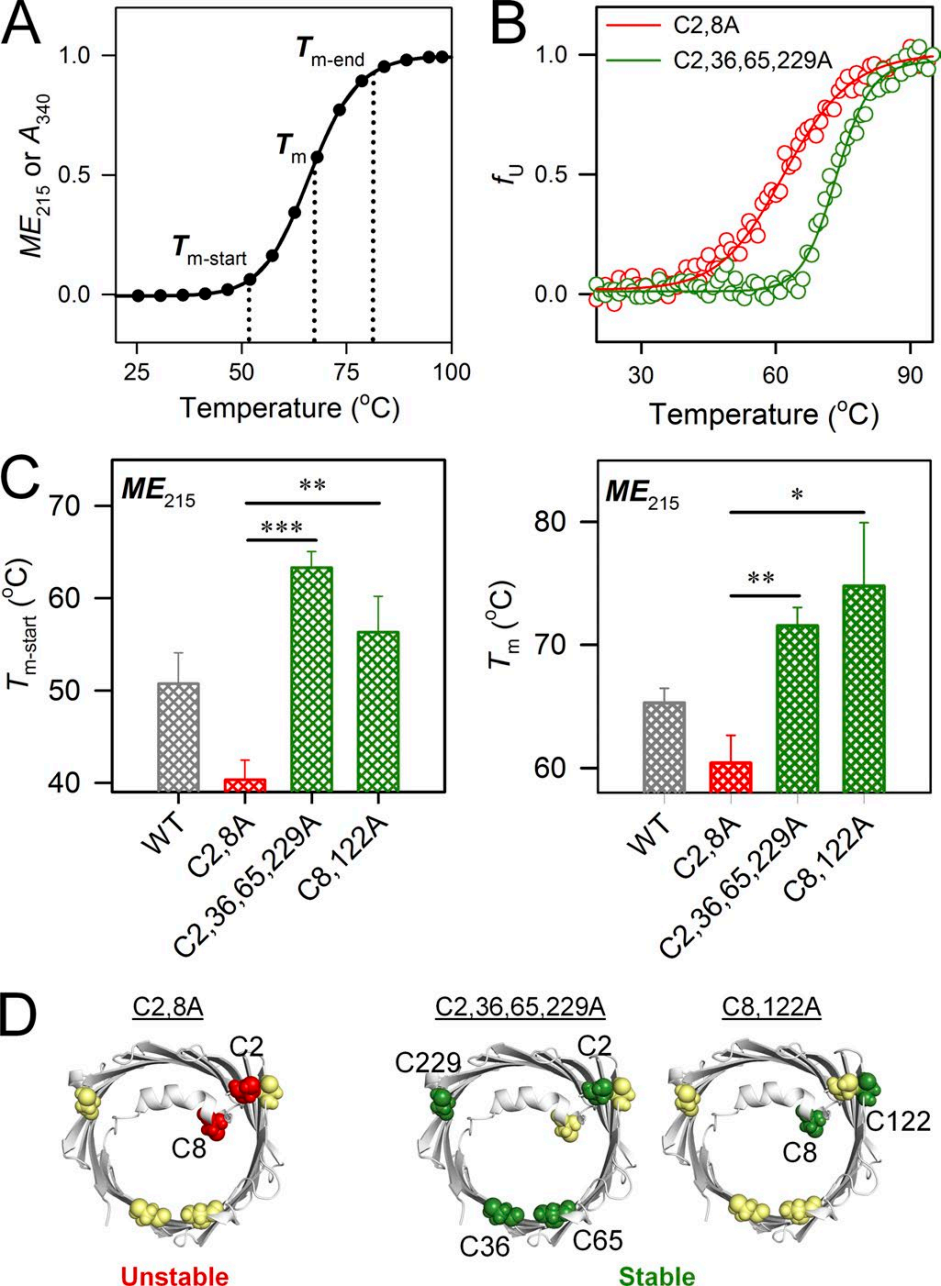
**Stability of hV3 is modulated by specific cysteine residues. (A)** Schematic illustrating the thermal parameters measured using far-UV CD from changes in *ME*_215_ or absorbance at 340 nm (*A*_340_) normalized between 0 and 1. The profile illustrates *T*_m-start_, *T*_m_, and *T*_m-end_, used here as indicators of protein stability. Here, *T*_m-start_ = temperature where unfolding or aggregation is nucleated; *T*_m_ = the temperature where 50% unfolding or aggregation is achieved; and *T*_m-end_ = the temperature where unfolding or aggregation is completed. **(B)** Thermal denaturation profiles of C2,8A (red) and C2,36,65,229A (green) variants normalized between 0 and 1, highlighting the dependence of the measured thermal parameters on the mutation. Here, C2,36,65,229A protein exhibits high value of *T*_m-start_ and *T*_m_, denoting high stability. **(C)** Comparison of *T*_m-start_ and *T*_m_ derived from *ME*_215_ measurements for the most (C2,36,65,229A and C8,122A in green) and least (C2,8A in red) stable mutants of hV3, obtained from the global comparison of *T*_m-start_ and *T*_m_ from *ME*_215_ and *A*_340_ (see Fig. S2 C). Data for WT is shown in gray for comparison only. Error bars represent SD derived from a minimum of three independent experiments. Statistical significance (***, P < 0.001; **, P < 0.01; *, P < 0.05) was derived using *t* test (unpaired). **(D)** Schematic highlighting the importance of C2/8-C122 interactions for hV3 stability. When both C2 and C8 are mutated (red spheres) the protein is least stable (left); mutating or retaining both C8 and C122A as a pair stabilizes the barrel (right).

Next, we addressed the contribution of cysteines to hV3 stability and aggregation. As chemical denaturants dissolve protein aggregates, all hV3 mutants were subjected to thermal stress to initiate and accelerate protein unfolding and aggregation. This was necessary to study hV3 aggregation characteristics within realistic timescales. It must be noted here that in hV3, unfolding and aggregation events are coupled and quantitative demarcation of both processes is difficult; primarily, our spectroscopic measurements provide information on hV3 aggregation. Using far-UV CD, we monitored the loss in *ME*_215_ with increase in temperature and obtained the start (*T*_m-start_), midpoint (*T*_m_), and end temperatures (*T*_m-end_) of unfolding and aggregation (Fig. 5 A). If the measured value of thermal parameters (*T*_m-start_, *T*_m_, and *T*_m-end_) is high, it denotes that the protein is highly stable (Fig. S2 and Table 1). However, these parameters can vary independently, complicating the measurement of protein stability.

To obtain a reliable analysis of protein stability, unfolding, and aggregation, we prioritized those mutants with most significant stabilizing or destabilizing features across the experimentally measured parameters listed in Table 1 (see Materials and methods for more details). In this process, the major changes that are noteworthy are compared easily without influence from minor variations, and meaningful conclusions can be drawn. Additionally, our experimental conditions are chosen such that they are least influenced by the protein concentrations used (discussed earlier).

hV3 shows irreversible thermal denaturation and exhibits a two-state unfolding profile, where unfolding is coupled with protein aggregation. Interestingly, while the thermal parameters vary across the mutants (see Fig. S2 for complete data), the WT and C0A show similar values of *T*_m-start_ and *T*_m_, which again suggests that some cysteines are stabilizing while others may have a destabilizing role. In addition, the thermal parameters do not correlate with the total cysteine content of the protein (Fig. S2), suggesting that hV3 stability is dependent on the presence or absence of specific cysteines.

Of particular interest are the C2,36,65,229A and C8,122A mutants that are highly stable, as well as the C2,8A (highly destabilized; Fig. 5, B and C). First, although C8,122A shows lowered secondary structure (Fig. 2 D), the protein is surprisingly stable to thermal denaturation (high *T*_m-start_ and *T*_m_; Fig. 5 C). Second, when only C8 and C122 are retained or removed (C2,36,65,229A and C8,122A mutants, respectively), hV3 stability is high. Third, nearly all the variants where C122A is mutated are stable (Fig. S2). The common feature we identify from these results is that mutating α1 cysteines (C2 and C8) lowers hV3 stability, whereas mutating only the β7–β9 cysteine (C122) increases barrel stability (Fig. 5, C and D). Although C122 promotes hV3 structure (Fig. 2 D), stability of the region corresponding to β7–β9 appears intrinsically lowered. On the other hand, C2 and C8 contribute to stabilizing hV3. Hence, C2/C8 appears to interact with C122 and modulate the hV3 stability.

In folded hV3, C122 is located in the loop and maps to the region between strands β7 and β9. C2 and C8 are present in the N-terminal helix α1. In hV3, α1 docks on β10–β15 and forms an interaction network with β7–β9 (Fig. 2 A). In hVDAC1, L21 (on α1) and V154 (on β9) form stabilizing interactions required for helix docking (Zachariae et al., 2012). Our previous observations from single-channel conductance and cell survival studies of hV3 reaffirms that this α1 interaction is important for VDAC stability and function (De Pinto et al., 2016; Reina et al., 2016a). We also obtained evidence for the importance of the α1–β7–β9 interaction from the *T*_m-end_ data, as well as by directly monitoring hV3 aggregation kinetics using CD, dynode voltage (*HT*_215_), and UV scattering spectroscopy (absorbance at 320 nm, *A*_320_; or 340 nm, *A*_340_; details are in Figs. S2, S3, S4, and S5). Hence, the structural and stability measurements (Figs. 2 D and 5 C) together suggest that the β7–β9 segment, important for hV3 structure, is stabilized by α1 (α1–β7–β9 interaction).

### Activation energy measurements support the importance of α1–β7–β9 interaction

**Figure 6.**
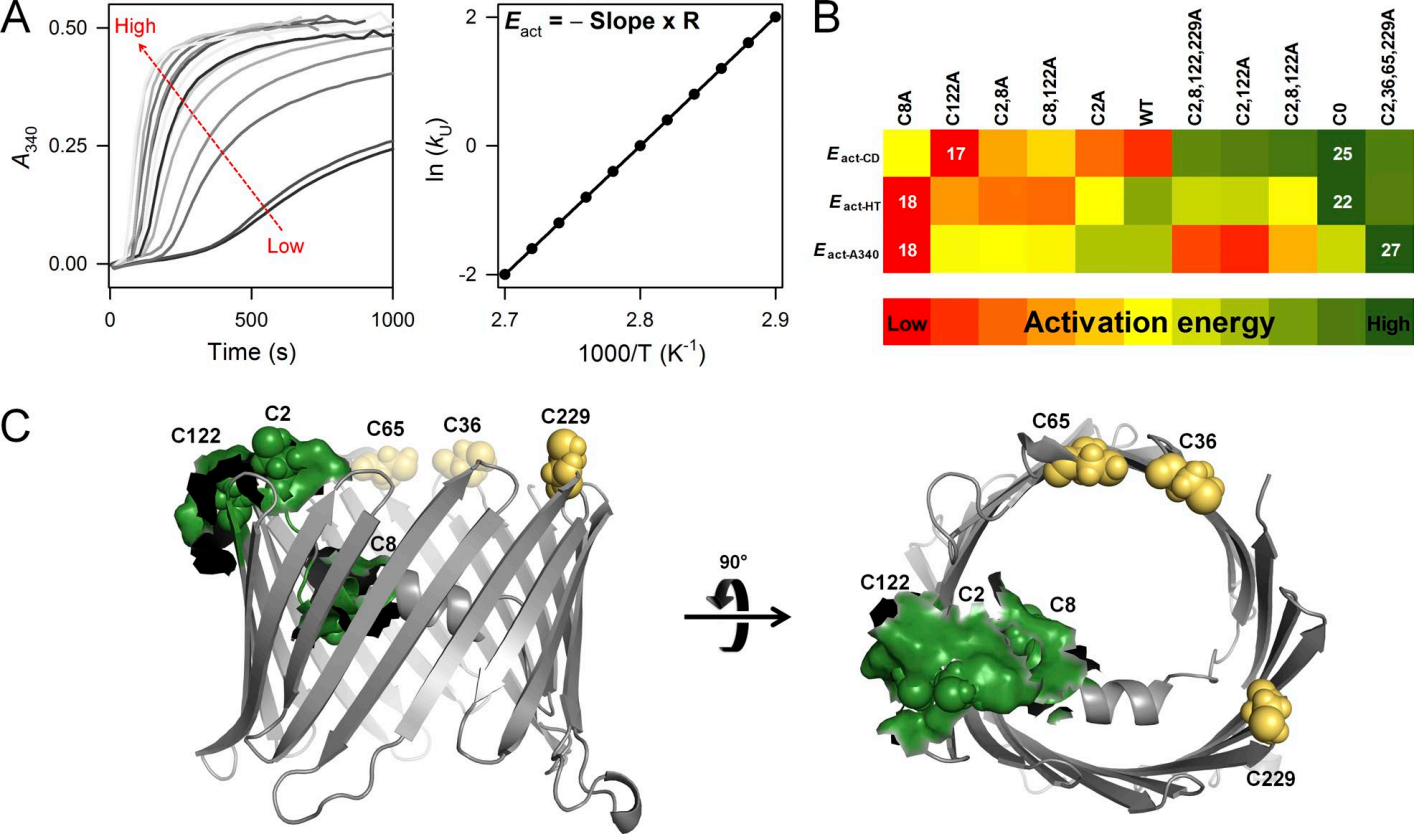
**Stability of hV3 is modulated by interaction of β7–β9 with the N-helix. (A)** Representative data illustrating how the rate of increase in *A*_340_ is faster at higher temperatures (left). The rate of aggregation proportionally increases with temperature. The data were fitted to an exponential function to obtain the rates of unfolding and aggregation (see Fig. S3). The rates were then fitted to a polynomial function to derive the *E*_act_ (right). Schematic illustrating how *E*_act_ is calculated (right). R = 1.987 kcal K^−1^ mol^−1^. **(B)** Global comparison of *E*_act_ derived from *ME*_215_, *HT*_215_, and *A*_340_, using a heat map scheme. Mutants with the highest *E*_act_ are the most stabilizing and are on the right (green); destabilizing mutants show the lowest *E*_act_ and are on the left (red). In line with stability measurements in Fig. 5, the *E*_act_ also suggest that C2,36,65,229A variant is most stabilizing owing to the presence of C8, C122; and removal of C2, C8, or C122 destabilizes the barrel (see Fig. S4 for details). **(C)** Stabilizing α1-β7-β9 interaction is shown in green as a surface rendering on the hV3 structure. This stabilizing interaction is formed via interaction of C2, C8, and C122 (green spheres; other cysteines are in yellow).

From the thermal denaturation measurements, it is clear that cysteines are important for hV3 scaffold and stability. To study the α1–β7–β9 (C2/C8–C122) interaction further, we measured the activation energy (*E*_act_) barrier for hV3 aggregation under thermal stress. *E*_act_ estimates the energy barrier separating the native and aggregated states of a protein. Here, the rate of unfolding (*k*_U_) increases proportionately with temperature (Fig. 6 A, left, and Fig. S3) and a plot of ln*k*_U_ against temperature can be fitted to the Arrhenius equation to derive the *E*_act_ (Fig. 6 A, right).

hV3 aggregates upon unfolding, and the two processes are coupled; the β-barrel shows a transition from the folded to the aggregated state. As the extent of aggregation is protein concentration dependent, we first tested the effect of hV3 concentration and the ramp rate on the measured data. We obtained a linear dependence of the protein concentration on the *T*_m_ and ramp rate; a nonlinear variation was seen only in very high DPRs (see Fig. 3 for details). Our experimental conditions (5 µM hV3, DPR ≈ 8,500:1) fall well within the linear correlation zone, suggesting that we can study hV3 aggregation without significant influence from the absolute protein concentrations used.

For the *E*_act_ measurements, we monitored the coupled unfolding and aggregation using the change in *ME*_215_ (secondary structure), as well as *HT*_215_ and *A*_340_. The *E*_act-ME215_ monitors the coupled unfolding and aggregation, while *E*_act-HT_ and *E*_act-A340_ measure scattering of light by protein oligomers or aggregates and are a direct measure of the aggregation process. The aggregation kinetics of hV3 exhibits a lag phase, followed by an exponential rise (Fig. 6 A, left, and Fig. S3). To simplify the analysis, we ignored the lag phase and derived the *k*_U_ from the exponential phase (see Materials and methods for more details).

The results are summarized in Figs. 6 B, S3, and S4. In line with stability measurements, variations in the *E*_act_ are influenced by specific cysteines. When C8 is mutated (C8A, C2,8A, C8,122A), the hV3 barrel is destabilized considerably, and rapid protein unfolding and aggregation ensues (Figs. 6 B and S4). This effect is not rescued even if the other cysteines are retained, reaffirming the importance of C8 for hV3 stability. Additionally, the construct that retains the C8–C122 interaction (C2,36,65,229A) is the most stable mutant (Fig. 6 B), suggesting that a highly specific interaction is formed between C8 and C122. Overall, results from the *E*_act_ measurements validate that C8–C122 interaction stabilizes hV3.

Notably, we obtained a non-Arrhenius behavior for the C0 mutant upon thermal denaturation (Fig. S4). Here, we observed two aggregation phases (ln*k*_u-A340_) below and above ∼88°C. Such a non-Arrhenius behavior has been observed for other proteins such as lysozyme (Matagne et al., 2000) and is believed to arise either from the population of intermediates in the unfolding and/or aggregation pathway or the existence of an alternate pathway. Because of the complex nature of hV3 aggregation, we did not study this deviation in C0 any further.

To further understand the importance of the α1–β7–β9 interaction in hV3 aggregation, we measured the aggregation index (*AI*_320_ or *AI*_340_) and end-point temperature of aggregation (*T*_m-end_; Figs. S5 and S6). Here, a high *T*_m-end_ denotes a stable protein that requires very high temperatures to unfold and aggregate. Similarly, a low *AI*_320_ or *AI*_340_ also indicates a stable barrel that forms small aggregates (Fig. S6). Similar to stability measurements, C8,122A, C2,36,65,229A, and C2,8,122A variants are the most stable and exhibit the lowest aggregation propensity. Hence, the stability and lowered aggregation propensity are directly related. Here again, when the α1–β7–β9 interaction is perturbed by mutating C2 and C8 and retaining only C122 in the C2,8A mutant, hV3 aggregation is pronounced (Fig. S6). Further, similar aggregation propensity of WT and C0A suggests that hV3 aggregation is controlled by specific cysteines. Interestingly, the C2,8,122,229A mutant shows the highest aggregation propensity, despite being moderately stable. We also note here that C36 (loop connecting β1–β2) and C65 (loop connecting β3–β4) have been mutated in C2,36,65,229A. While our data suggest the likely existence of a second oligomerization site at β1–β4 (also see Schredelseker et al. [2014]), direct evidence for this is presently limited (see Supplemental notes for details).

Our results from thermal denaturation (Fig. 5) show that the β7–β9 zone is intrinsically destabilized in hV3. Considering that docking of the N-helix is favored in VDAC scaffolds (as opposed to a highly dynamic helix; Teijido et al., 2012; Zachariae et al., 2012), we propose that the destabilization at β7–β9 is offset by a highly stabilizing interaction network formed between α1–β7–β9 (Fig. 6 C). Indeed, simulation studies show that the VDAC barrel is more dynamic and exhibits a more elliptical barrel when the N-helix is removed. In the presence of α1, the barrel wall is rigidified, and the scaffold exhibits lower overall dynamicity (Zachariae et al., 2012). When residues in the α1 segment of hV3 are mutated, it is likely that the interaction network is disrupted and the barrel is destabilized.

In principle, the substitution of cysteine to alanine should not considerably perturb the α1–β7–β9 interaction, as neither residue establishes strong electrostatic or hydrophobic interactions in the structure. However, our experiments clearly reveal that the mutation sufficiently perturbs the hV3 structure to lower the scaffold stability and drive protein aggregation. Based on our data, we conclude that the α1–barrel interaction is crucial for VDAC stability, and even minor perturbations in this docking interaction can adversely affect the protein stability.

### VDAC aggregation proceeds likely through structured oligomers

**Figure 7.**
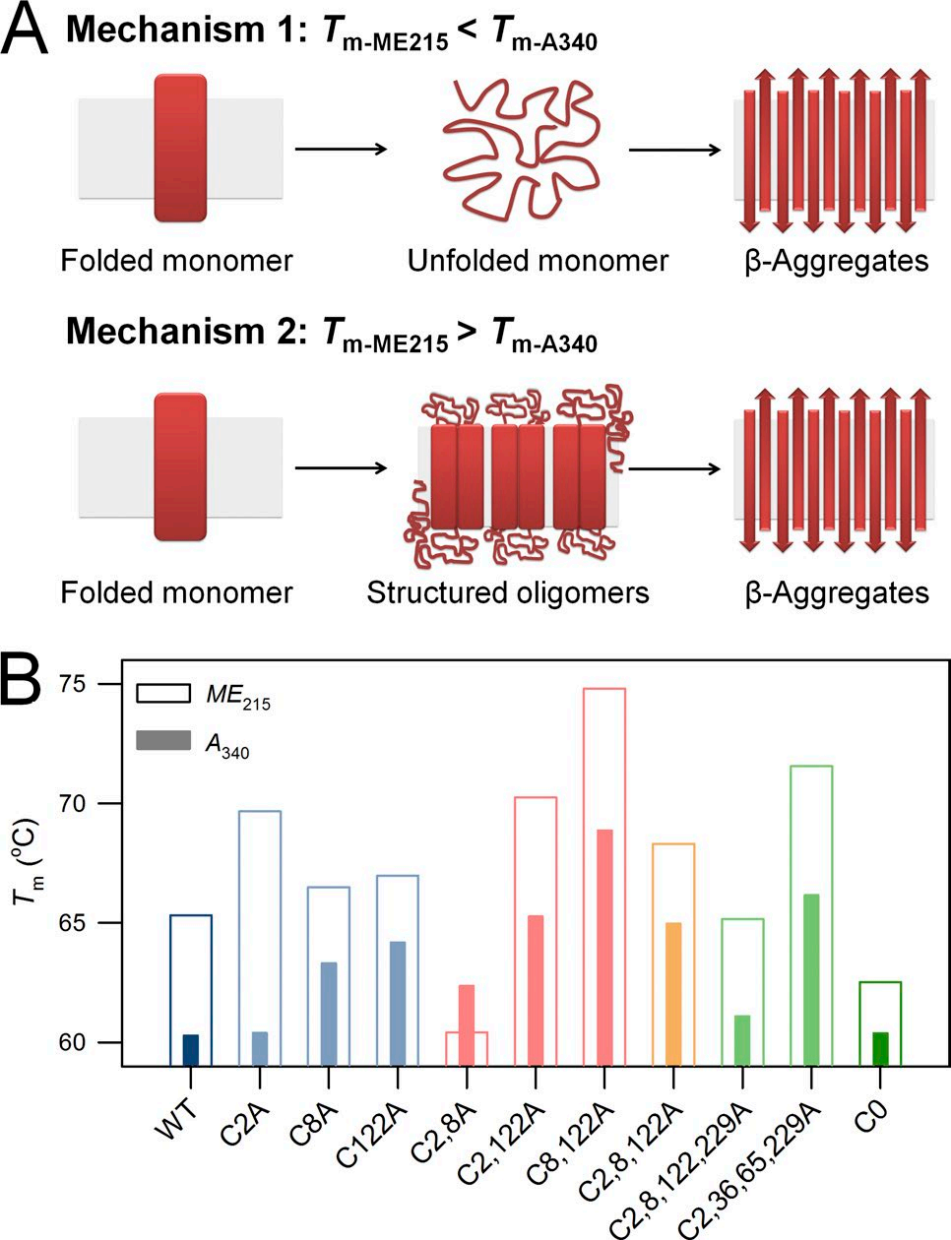
**Understanding the mechanism of hV3 unfolding and aggregation. (A)** Schematic of how possible aggregation mechanisms affect the measured *T*_m_. A lower value of *T*_m-ME215_ than *T*_m-A340_ suggests the transition of native protein to unfolded species followed by the formation of structured aggregates (Mechanism 1). On the other hand, *T*_m-ME215_ greater than *T*_m-A340_ indicates that the folded protein directly forms structured aggregates during the unfolding process; hence, the unfolded species is not directly detected (Mechanism 2). **(B)** Histogram comparing *T*_m-ME215_ and *T*_m-A340_. A rainbow color pattern is used to represent the progressive lowering in cysteine content from hV3 WT (dark blue; left extreme) to the Cys-less mutant C0 (dark green; right extreme). Here, WT and all mutants show *T*_m-A340_ < *T*_m-ME215_, indicating hV3 oligomerization and aggregation before complete unfolding or loss in secondary structure (see Fig. S7 for details). The only deviation is observed in C2,8A (see text for discussion). Overall, hV3 follows Mechanism 2, where protein unfolding and aggregation are coupled.

How do VDACs aggregate? Aggregation largely follows protein unfolding; however, folded proteins can also aggregate by oligomerization (Soto, 2003). To measure the process of hV3 aggregation, we performed scattering measurements at 320 and 340 nm (*A*_320_ and *A*_340_). At these wavelengths, the formation of both soluble and particulate aggregates can be detected. We compared the thermal parameters obtained from scattering (*T*_m-A320_ and *T*_m-A340_) with changes in secondary structure content obtained from *ME*_215_ (*T*_m-ME215_). The *T*_m-A320_ and *T*_m-A340_ provide information on hV3 aggregation, whereas *T*_m-ME215_ represents both unfolding and aggregation. By comparing both the parameters, we can obtain information on whether unfolding precedes aggregation, or vice versa. A lower *T*_m-ME215_ than *T*_m-A340_ indicates that the loss in secondary structure precedes aggregation (Mechanism 1; Fig. 7 A). A higher value of *T*_m-ME215_ indicates the formation of oligomers and aggregates before considerable loss in protein structure occurs (Mechanism 2; Fig. 7 A).

With the exception of C2,8A, we find that hV3 WT and all the mutants exhibit a *T*_m-ME215_ that is ∼3°C to 8°C higher than *T*_m-A340_ (Figs. 7 B and S7). Based on these results, we conclude that hV3 oligomerization and aggregation occur well before complete loss in protein structure and give rise to aggregates with significant β-sheet content (as observed from *ME*_215_). In C2,8A, mutating the α1 cysteines lowers the stability of hV3 (lower *T*_m-ME215_), and thereby causes protein unfolding at temperatures lower than the other mutants. Overall, our work and that of others (Okazaki et al., 2015; Reina et al., 2016a) indicate that hV3 exhibits the propensity to form nondisulfide oligomers. Such oligomeric species may give rise to structured aggregates in the cell under adverse cellular conditions including oxidative modifications (Okazaki et al., 2015; Reina et al., 2016a).

**Figure 8.**
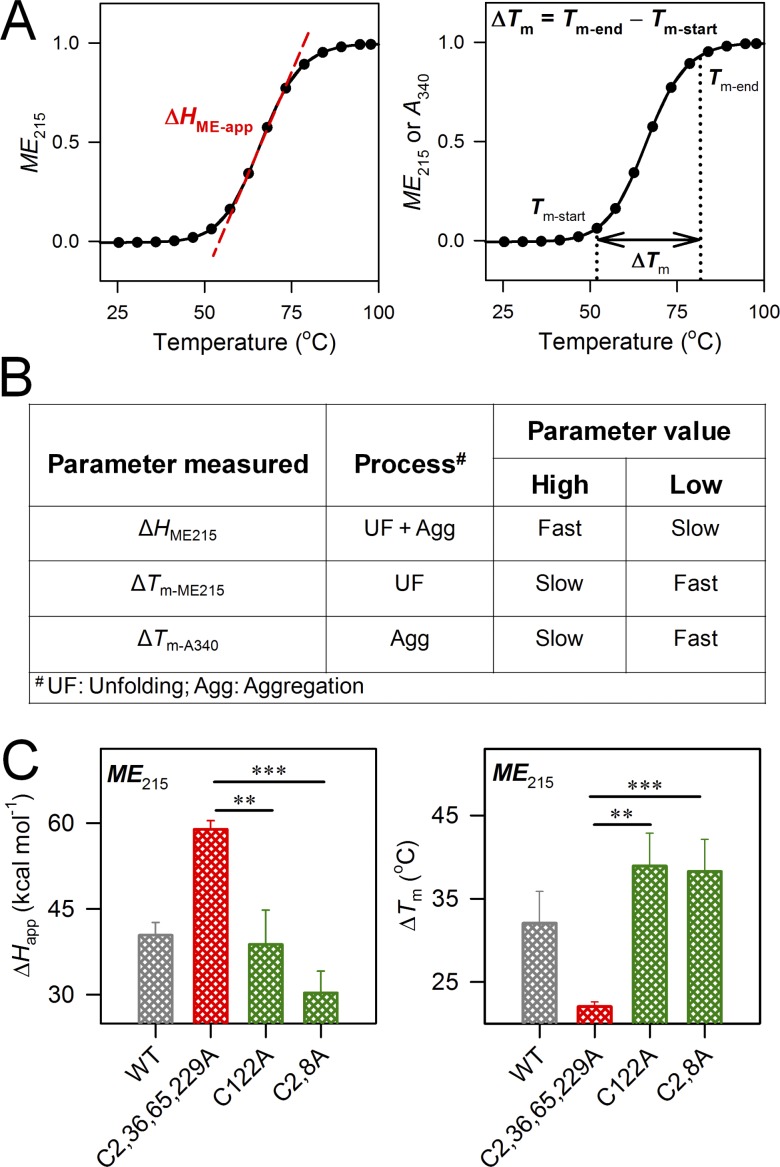
**Deducing the mode of hV3 aggregation. (A)** Schematic of how cooperativity of unfolding and aggregation are measured as Δ*H*_ME-app_ (left) and Δ*T*_m_ (right), with values normalized between 0 and 1. **(B)** Summary of how the kinetics of unfolding and aggregation can be inferred from Δ*H*_ME-app_ and Δ*T*_m_. Comparison of Δ*H*_ME-app_ and Δ*T*_m_ is useful to study the aggregation propensity and rate of aggregation of proteins. A high or low value of various parameters indicates unfolding, aggregation, or unfolding coupled with aggregation. **(C)** Comparison of Δ*H*_ME-app_ and Δ*T*_m_ calculated from *ME*_215_. Here, the most stabilizing and destabilizing mutants derived based on the global comparison of Δ*H*_ME-app_ and Δ*T*_m_ are shown, as described in Fig. S8. Data for WT is shown in gray for comparison only. Error bars represent SD derived from a minimum of three independent experiments. Statistical significance (***, P < 0.001; **, P < 0.01) was derived using *t* test (unpaired). See Figs. S7 and S8 for the complete data and comparison using the heat map scheme.

We also examined the molecular elements of the barrel that promote hV3 oligomerization and aggregation. For this, we used two thermal parameters, namely Δ*H*_ME-app_ (apparent unfolding enthalpy) and Δ*T*_m_ (difference between *T*_m-end_ and *T*_m-start_; Table 1, Fig. 8 A, and Fig. S8). The Δ*H*_ME-app_ is derived from the change in *ME*_215_ when hV3 is subjected to thermal denaturation and denotes the cooperativity of the transition once protein unfolding is nucleated. A high Δ*H*_ME-app_ (and a corresponding low Δ*T*_m_) indicates that aggregation is highly cooperative (Fig. 8 B).

**Figure 9.**
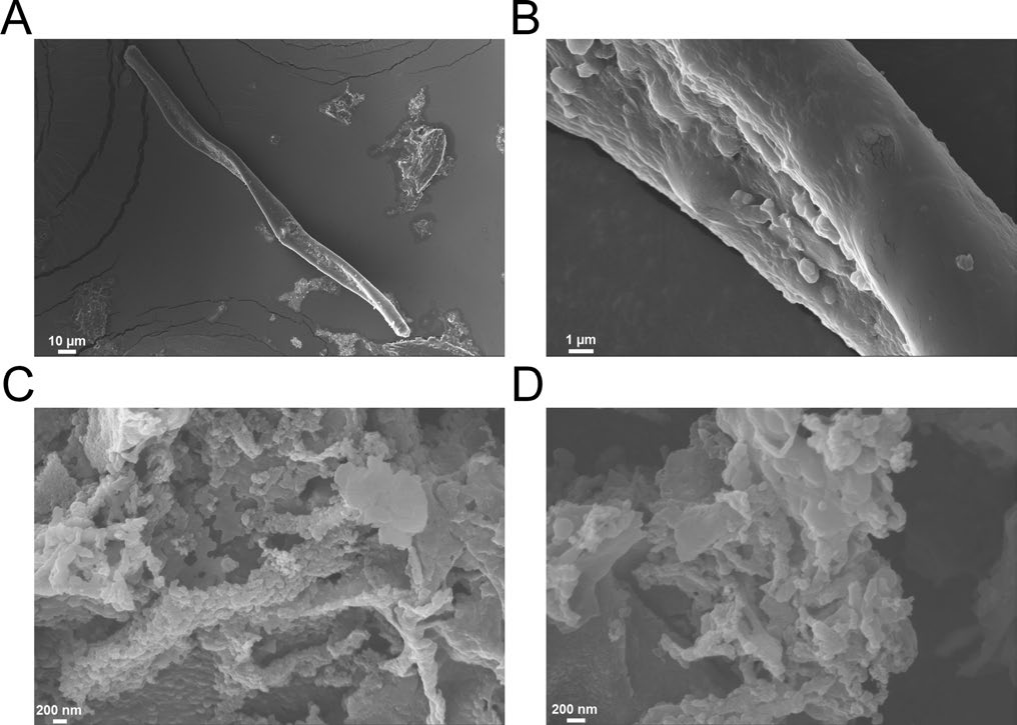
**hV3 shows dual morphologies in the aggregated state.** Representative SEM images of aggregated hV3 WT. **(A and B)** Here, hV3 shows the presence of amyloidogenic fiber-like aggregates presented in different magnifications (low magnification, A; high magnification, B). **(C and D)** hV3 also shows the formation of globular mesh-type aggregates (low magnification, C; high magnification, D). Hence, the aggregated sample of hV3 contains a mixture of structured and amorphous aggregates. Note that we diluted the aggregated samples considerably for SEM experiments to observe well-separated images of the amyloid fibrils.

A comparison of the Δ*H*_ME-app_ and Δ*T*_m_ for the mutants with highest and lowest values is shown in Fig. 8 C. Once aggregation is nucleated, we find that the process is highly cooperative when C8 and C122 are retained (C2,36,65,229A). These aggregates show fluorescence upon binding to ThT (Fig. S9), and SEM images convincingly show distinct amorphous morphologies as well as fibrillar structures (Fig. 9). Mutating the cysteines in the α1–β7–β9 interaction triad lowers the secondary structure (Fig. 2 D), stability (Figs. 5 C and 6 B), and cooperativity of aggregation (Fig. S10). Considering that VDACs do not aggregate under physiological conditions (Betaneli et al., 2012), these interaction surfaces involving β7–β9 must be required for protein oligomerization in the membrane. Our conclusion is supported by the identification of an oligomerization zone at β7–β10 in hVDAC2 (Naghdi et al., 2015).

In silico analysis of the potential VDAC aggregation hotspots using 20 different tools (online servers for predicting aggregation-prone regions; see Materials and methods and Supplemental notes for details) shows that β4 and β9 are aggregation prone (Figs. S11, S12, and S13; also see Supplemental notes for details). When we compare the aggregation zones predicted from in silico analysis, across the VDAC isoforms, we find that most of the aggregation hotspots are common across the three isoforms. Thus, we speculate that all three VDACs offer similar surfaces for oligomerization or aggregation. These results are in line with our experimental data where we find that strands β7–β9 are aggregation prone. Our peptide-based study to map VDAC aggregation also indicates aggregation prone zones in β6–β11 for hV3 (Lella and Mahalakshmi, 2018). Hence, specific segments of the barrel involving β7–β9 that constitute oligomerization and aggregation loci modulate hV3 aggregation. Further, the importance of the α1–β7–β9 interaction is evident from our spectroscopic studies. Putting together our results from all the thermal parameters, we conclude that the α1–β7–β9 interaction promotes hV3 structure and stability and provides an oligomerization interface for the barrel at β7–β9. Under adverse cellular conditions, modification of C2/8 and C122 lowers hV3 stability, causing protein aggregation. These aggregates possess β-sheet content, have both fibrillar and amorphous appearance, and can be formed with partially unfolded hV3. The N-helix is physiologically relevant in voltage gating of VDACs (Reina et al., 2010, 2016a) and in scaffold stability (Zachariae et al., 2012; Maurya and Mahalakshmi, 2015). We find that an additional vital role of α1 is in suppressing VDAC aggregation.

### Conclusions

**Figure 10.**
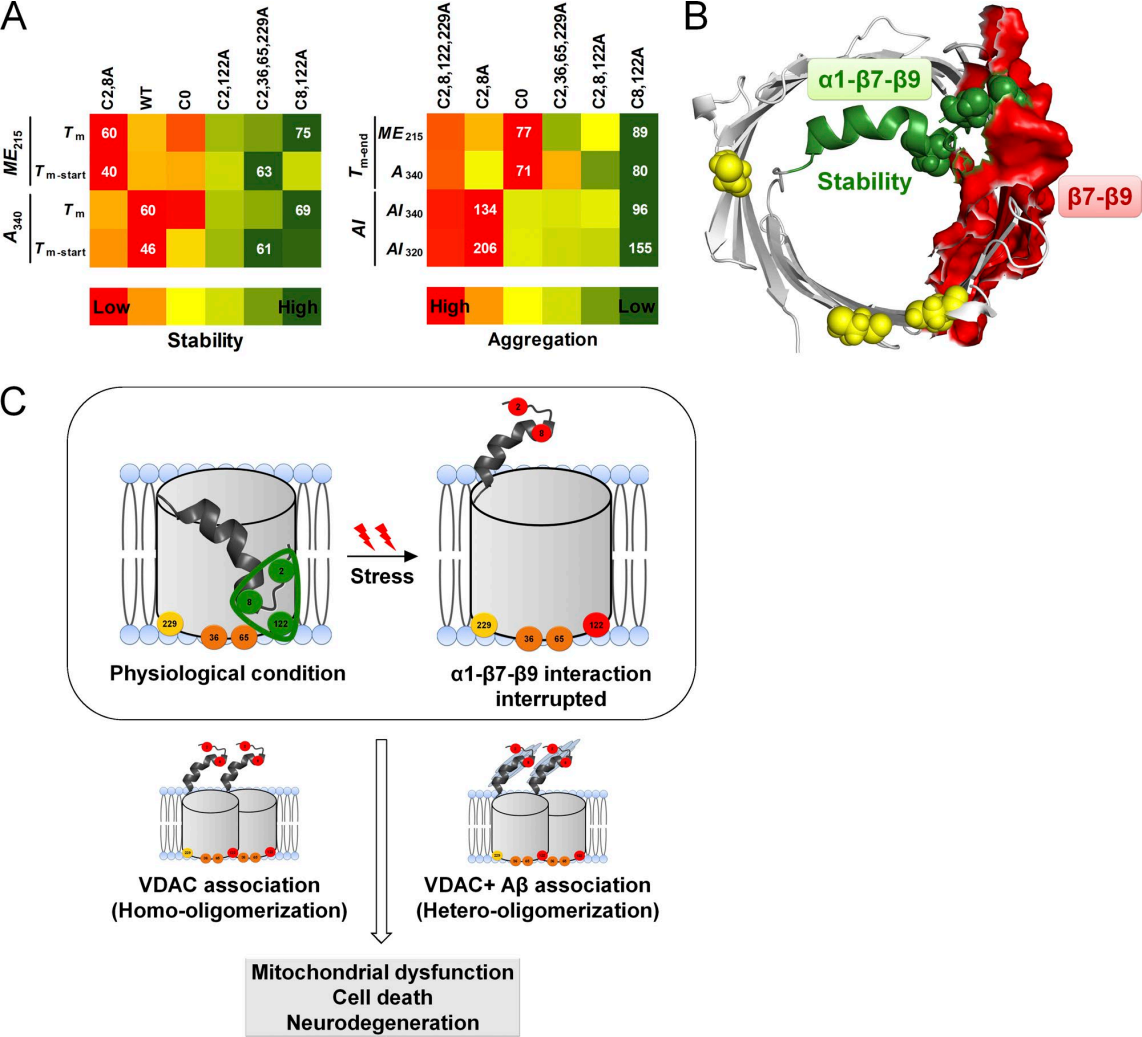
**Summary of scaffold stabilizing interactions and aggregation loci of VDACs. (A)** Summary of the stable and aggregation prone variants deduced from this study. The heat maps show the stability (left) and aggregation tendency (right) for most and least stable hV3 mutants. Numbers in the heat map show the data range for each parameter. The C8,122A and C2,36,65,229A mutants are most stable and exhibit least aggregation across the mutants (A, right side in the heat map, green color). The C2,8,122,229A and C2,8A mutants show highest aggregation propensity because of the presence of C36 and C65 pair (right; left side in the heat map, red color). Note that the presence of C36 and C65 does not considerably influence protein stability (see Fig. S2 C). Overall, the results show the importance of C2, C8, and C122 (α1–β7–β9 interaction triad) for the hV3 stability, and involvement of C36 and C65 for hV3 aggregation. **(B)** Schematic highlighting the α1–β7–β9 interaction triad (surface presentation, green color), dictating stability of the hV3 barrel. When this interaction is disrupted, hV3 is destabilized, and strands β7–β9 now become aggregation prone (shown as red surface presentation). **(C)** Putative model for VDAC aggregation in the cell. The α1–β7–β9 interaction triad (green triangle; C2, C8, C122 as green spheres) may suppress VDAC aggregation under physiological conditions (top left). During cellular stress, this stabilizing α1–β7–β9 interaction (C2, C8, C122 now shown as red spheres) may be disrupted (top right). Evidence for this destabilization is available for irreversible VDAC oxidation (Reina et al., 2016a) and sequestering of the α1 helix by Aβ (Smilansky et al., 2015). Both the processes can induce VDAC oligomerization (lower panel) at the aggregation hotspots. Progressive VDAC aggregation over a prolonged time may result in cell death and neurodegeneration.

VDACs play a central role in cellular bioenergetics (Reina et al., 2016a; Maurya and Mahalakshmi, 2017; Shoshan-Barmatz et al., 2017). VDACs have an inherent tendency to oligomerize, but they do not aggregate in the cell. They hetero-oligomerize with several other proteins including Aβ peptide, α-synuclein, tau, and SOD. Their involvement in diverse cellular processes also links VDACs to the development of pathologies including neurodegenerative diseases such as PD, amyotrophic lateral sclerosis, and AD (Smilansky et al., 2015; Magri and Messina, 2017; Sorrentino et al., 2017). Although VDACs are not known to intrinsically aggregate, they surprisingly present one of the key binding sites for Aβ peptide and are known to modulate Aβ toxicity (Cuadrado-Tejedor et al., 2011; Thinnes, 2011; Smilansky et al., 2015). Other misfolded proteins that cause neuronal dysfunction, including tau, Parkin, and α-synuclein, are also known to bind mitochondrial VDACs (Table S1), causing mitochondrial dysfunction, leading to cellular toxicity (Rostovtseva et al., 2015; Magri and Messina, 2017; Maurya and Mahalakshmi, 2017; Shoshan-Barmatz et al., 2017; Sorrentino et al., 2017; Iadanza et al., 2018). The ability of VDACs to bind and coaggregate with these proteins suggests that VDACs might indeed be predisposed to aggregation (Table S1). Through this study, we find that the intrinsic aggregation-prone region of hV3 maps to strands β7–β9 (Fig. 10). A secondary aggregation site near β1–β4 may further promote VDAC aggregation. Our findings are in good agreement with an earlier structure-based prediction of hVDAC1 oligomerization sites (Geula et al., 2012), crystal structure of zebrafish VDAC2 (Schredelseker et al., 2014), in vivo studies of hVDAC2 (Naghdi et al., 2015), and our peptide-based mapping of hVDAC1–3 aggregation hotspots (Lella and Mahalakshmi, 2018). We also show that the interaction network established between α1–β7–β9 stabilizes the hV3 scaffold and can suppress protein aggregation (Fig. 10) and help maintain proteostasis under physiological conditions.

The importance of cysteines in hV3 channel gating and in oxidative thiol modifications (Okazaki et al., 2015; Reina et al., 2016a) allows us to speculate a functional role for the α1–β7–β9 interaction. Under physiological conditions, the voltage sensor α1 docks near β7–β9; the α1–β7–β9 interaction stabilizes the barrel and suppresses nucleation of aggregation. Evidence for this docking is also available from studies on other VDACs (Magri and Messina, 2017; Maurya and Mahalakshmi, 2017). We find that hV3 aggregation is low when C122 is mutated, suggesting that β7–β9 is intrinsically destabilized. Hence, the vicinity of C122 is a potential nucleating site for aggregation, which is moderated via the α1–β7–β9 interaction. Deletion of α1 (Maurya and Mahalakshmi, 2015), or perturbing the α1–β7–β9 interaction through oxidative cysteine modifications (Reina et al., 2016a), can destabilize the barrel. Subsequently β7–β9 might be able to establish nonnative interactions through barrel oligomerization. Indeed, hetero-oligomerization of VDAC with tBid and BAK is known to occur through the β7–β10 region (Naghdi et al., 2015), supporting the predisposition of this zone to oligomerization. Based on our experimental results and findings from other laboratories (Geula et al., 2012; Schredelseker et al., 2014), we conclude that β7–β9 can form a potential oligomerization interface in VDACs. Perturbation of the α1–β7–β9 interaction over a prolonged time can cause progressive aggregation of VDACs. Notably, these aggregates are extramembranous, highlighting the nonnative interactions established by VDACs with other aggregation-prone proteins. For example, Aβ peptide interacts with and sequesters α1 of hV1, thus affecting channel function and causing VDAC aggregation (Fig. 10 C; Smilansky et al., 2015). These observations highlight the importance of α1 in suppressing VDAC aggregation.

Our study couples spectroscopic methods with cysteine perturbations to allow for the rapid identification of molecular elements that stabilize hV3 and factors that could lead to barrel oligomerization and aggregation. While the complete pathway of VDAC-mediated aggregation still requires exploration, combining our findings with previous in vitro and in vivo observations reveals similar interaction interfaces in all three VDAC isoforms. These interaction interfaces can serve as sites for the rational design of VDAC aggregation blockers using the various available strategies. Additionally, we demonstrate how far-UV CD spectroscopy and UV scattering can be applied reliably to identify aggregation loci of membrane proteins. Our spectroscopic methods can open avenues for the mapping of similar aggregation sites of other membrane proteins.

## Supplementary Material

Supplemental Materials (PDF)

Figure S13 (PDF)
