# Supplementary material for: Helix–strand interaction regulates stability and aggregation of the human mitochondrial membrane protein channel VDAC3
Source: J Gen Physiol. 2019 Jan 23;151(4):489–504. doi: 10.1085/jgp.201812272 (PMC6445588; doi:10.1085/jgp.201812272)
Supplement: Supplemental Materials (PDF) [file JGP_201812272_sm.pdf]

Supplemental material

Gupta et al., https://doi.org/10.1085/jgp.201812272

Notes

hV3 is likely to possess a primary and secondary aggregation site

Our studies also indicate the presence of a second oligomerization site at β1–β4. We obtain data for the second oligomerization site from the C2,8,122,229A mutant. The C2,8,122,229A mutant shows the highest aggregation propensity despite having moderate stability (Figs. S2, S4, S5, and S6). We find that hV3 aggregation is prominent when C36 (strand β1) and C65 (strand β4) are retained (see C2,8,122,229A mutant in Figs. S5 and S6). In line with this observation is the lowered aggregation rates for C2,36,65,229A, where both C36 and C65 have been mutated to alanine. Our observation is in line with previous results (Schredelseker et al., 2014).

β1–β4 forms a part of β17–β19–β1–β3, which is a known oligomerization interface of VDACs (Schredelseker et al., 2014; Bergdoll et al., 2018). Notably, higher-order oligomeric forms of VDAC isoforms (tetramers–octamers) have been observed in crystals as well as in fluorescence imaging studies (Hoogenboom et al., 2007; Raschle et al., 2009; Betaneli et al., 2012; Yu et al., 2012; Schredelseker et al., 2014). Oligomeric forms of the C2,8,122,229A mutant are also visible during electrophoretic separation of unboiled samples (Fig. S1). The oligomerization and aggregation characteristics of this mutant are more prominent than the C2,8A mutant, wherein the interaction triad is disrupted (Figs. S5 and S6). Further, the rate of unfolding and aggregation is high when only C36 and C65 are retained (discussed in Figs. 8 and S8). When we compare the variants of hV3 for pronounced aggregation characteristics (see Figs. S5, S6, S7, and S8), the underlying commonality we identify is that these mutants retain C36 (strand β1) and C65 (strand β4).

On the basis of our results, we hypothesize that the β1–β4 and β7–β9 regions of the barrel constitute two interaction surfaces, which also constitute aggregation loci (Fig. S6 C). Putting our observations together with previous studies, we find that VDACs possess an inherently destabilized β7–β9 region as well as are likely to possess an oligomerization and aggregation prone β1–β4 region.

In silico studies: Aggregation loci prediction using in silico methods

In silico prediction was executed using the protein sequences of hVDAC1, hVDAC2, and hVDAC3 (hV1, hV2, hV3). All three sequences were obtained from the NCBI database (hVDAC1: NP_003365.1; hVDAC2: NP_003366.2; hVDAC3: NP_005653.3). We used Tango (algorithm for prediction of aggregation prone regions in unfolded polypeptide chains; Fernandez-Escamilla et al., 2004), Waltz (algorithm for prediction of amylogenic regions in protein sequences; Maurer-Stroh et al., 2010), Aggrescan (algorithm that predicts aggregation-prone segments in protein sequences; Conchillo-Solé et al., 2007), FISH Amyloid (machine learning method for classification of amino acid sequences; Gasior and Kotulska, 2014), Zyggregator (aggregation propensity prediction based on polypeptide sequences; DuBay et al., 2004; Pawar et al., 2005; Tartaglia et al., 2008; Tartaglia and Vendruscolo, 2008), FoldAmyloid (predicts amyloidogenic regions in the protein chain; Garbuzynskiy et al., 2010), PASTA 2.0 (Prediction of Amyloid Structure Aggregation 2.0; Walsh et al., 2014), AMYL-PRED (Frousios et al., 2009), AMYL-PRED 2 (Tsolis et al., 2013), GAP (Generalized Aggregation Proneness; Thangakani et al., 2014), PaFig (Prediction of amyloid Fibril-forming segments; Tian et al., 2009), AmyloidMutants (predicts the structural and mutational landscapes of amyloid fibrils; O'Donnell et al., 2011), Amyloidogenic Pattern (identifies the sequence pattern related to the amyloid fibrils formation; López de la Paz and Serrano, 2004), MetAmyl (METapredictor for Amyloid proteins; Pawlicki et al., 2008), Average Packing Density (relates the Average Packing Density amino acid of stretches of residues to the formation of amyloid fibrils; Galzitskaya et al., 2006), β-Strand Contiguity (algorithm locates β-strands in the amyloid fibril core using amino acid sequence; Zibaee et al., 2007), Hexapeptide Conformational Energy (isolates aggregation-prone hexapeptides in the polypeptide chain; Zhang et al., 2007), NetCSSP (calculates influence of tertiary interactions on secondary structure propensity; Kim et al., 2009), Possible secondary structure Conformational Switches (predicts conformational switches for both α-helices and β-sheet strands; Hamodrakas et al., 2007), and CamSol (method for protein solubility prediction; Sormanni et al., 2015), for prediction of amyloidogenic regions in all three VDAC polypeptide sequences. Here, the raw protein sequence was used as input in each tool; all the tools were run with default settings, except PASTA 2.0, where the threshold was fixed at 90% for prediction of amyloidogenic segments, as it detects the amyloid hotspot with high confidence. The predicted amyloidogenic segments and residues are highlighted in pink color in the multiple sequence alignment (MSA) shown in Figs. S11 and S13. We assigned scores to each residue (0, not amyloidogenic; 1, amyloidogenic) for results obtained from each prediction tool. Then, we compared the aggregation propensity of the whole sequence across the prediction tools by the sum of scores. The consensus residues predicted from a minimum of four methods (aggregation tendency score of ≥20%) has been highlighted in the bottom panel of MSA as the consensus sequence (pink color in Figs. S11 and S13). The results are presented in the form of a histogram in Fig. S12 A. These segments are also mapped on the modeled structures of hV1, hV2, and hV3 (orange color; Fig. S12 B).

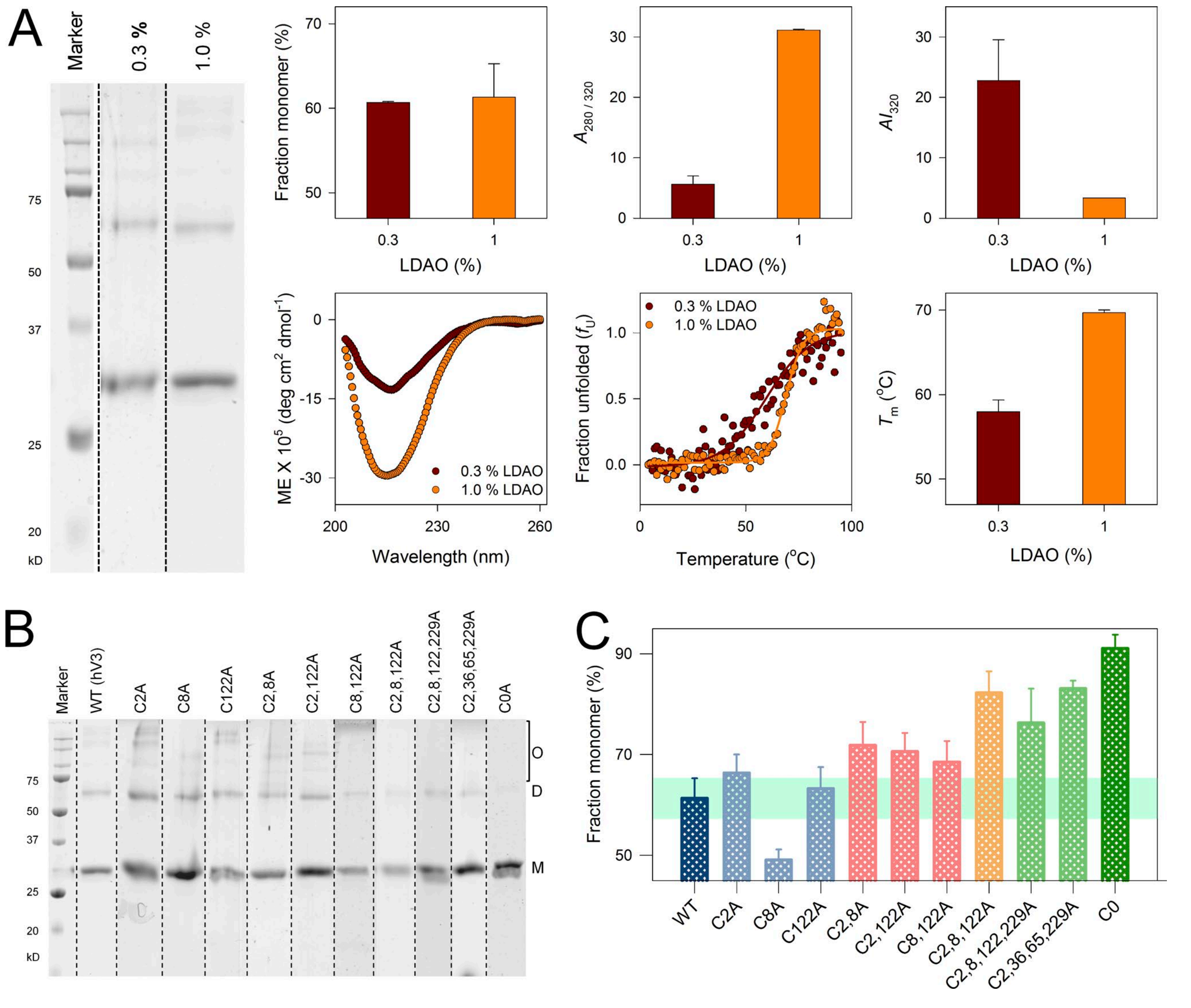

Figure S1. **Comparison of the oligomerization tendency of hV3 variants using electrophoretic mobility. (A)** Representative data showing results of screens with two DPRs (2,575:1, 0.3% LDAO, and 8,500:1, 1% LDAO). Compared here are the SDS-PAGE gel images for unboiled folded hV3 (WT), monomer fraction, absorbance at 280 and 320 nm ($A_{280/320}$) as a measure of protein aggregation, aggregation index (AI_{320}), far-UV CD wavelength scans, thermal denaturation profiles, and midpoint of thermal denaturation (T_m). High $A_{280/320}$, *ME*, T_m, and a corresponding low AI_{320} together indicate a well-folded protein. This is observed in 1% LDAO (DPR 8,500:1). Here, errors are SD derived from two independent experiments (except T_m, which represents goodness of fit). **(B)** Representative SDS-PAGE profiles of folded samples of all the cysteine variants prepared in final LDAO concentration of 1% (as described in Materials and methods). All the folded protein samples were diluted 1.25-fold in reducing dye containing 50 mM DTT. None of the samples were boiled before loading. Images from different gels for each mutant are separated using dotted lines and are presented together for easy comparison. The mutant names are provided above each lane. The profile for WT from A is retained here. The first lane represents the molecular weight marker, and relevant molecular weights (in kD) are indicated. M, monomer species; D, dimer species; and O, higher-order oligomer species observed on SDS-PAGE. **(C)** Fraction monomer (%) calculated from densitometry analysis of the SDS-PAGE profiles similar to the representative images shown in B. The total monomer content present in the folded sample was calculated by gel densitometry using MultiGauge v2.3 (as described in Materials and methods). Here, errors are SD derived from densitometry values of a minimum of two to three independent gels. The shaded area in all plots represents the SD obtained for hV3 WT protein, and is presented to highlight data of those mutants that are significantly different from the WT. A rainbow color pattern is used throughout to represent the progressive change in cysteine content from WT (dark blue; left extreme) to Cys-less mutant C0 (dark green; right extreme). Note that the fraction monomer (%) increases with lowering of the cysteine content of hV3 (lower panel) but lacks a direct correlation to the number of cysteines. For example, C8A (where only one cysteine is mutated) shows the least monomer population, while C2A and C122A (also mutants where only one cysteine is mutated) show monomer fractions that are comparable to WT and higher than C8A. Additionally, note how the triple- and tetra-Cys mutants show comparable monomer populations. Note here that DTT is introduced in our samples to avoid artifacts from disulfide-mediated oligomerization and to capture hV3 oligomerization that occurs via noncovalent (hydrophobic) interactions. Furthermore, our thermal denaturation measurements reveal that hV3 shows enhanced aggregation tendency when cysteines are removed (see Fig. S8). Hence, hV3 aggregation is not driven by disulfide bond formation; instead, specific cysteines can facilitate controlled barrel oligomerization transiently, under oxidative conditions in the cell. Further, nonspecific protein aggregation in the membrane is not driven by the formation of nonnative disulfide bonds in hV3.

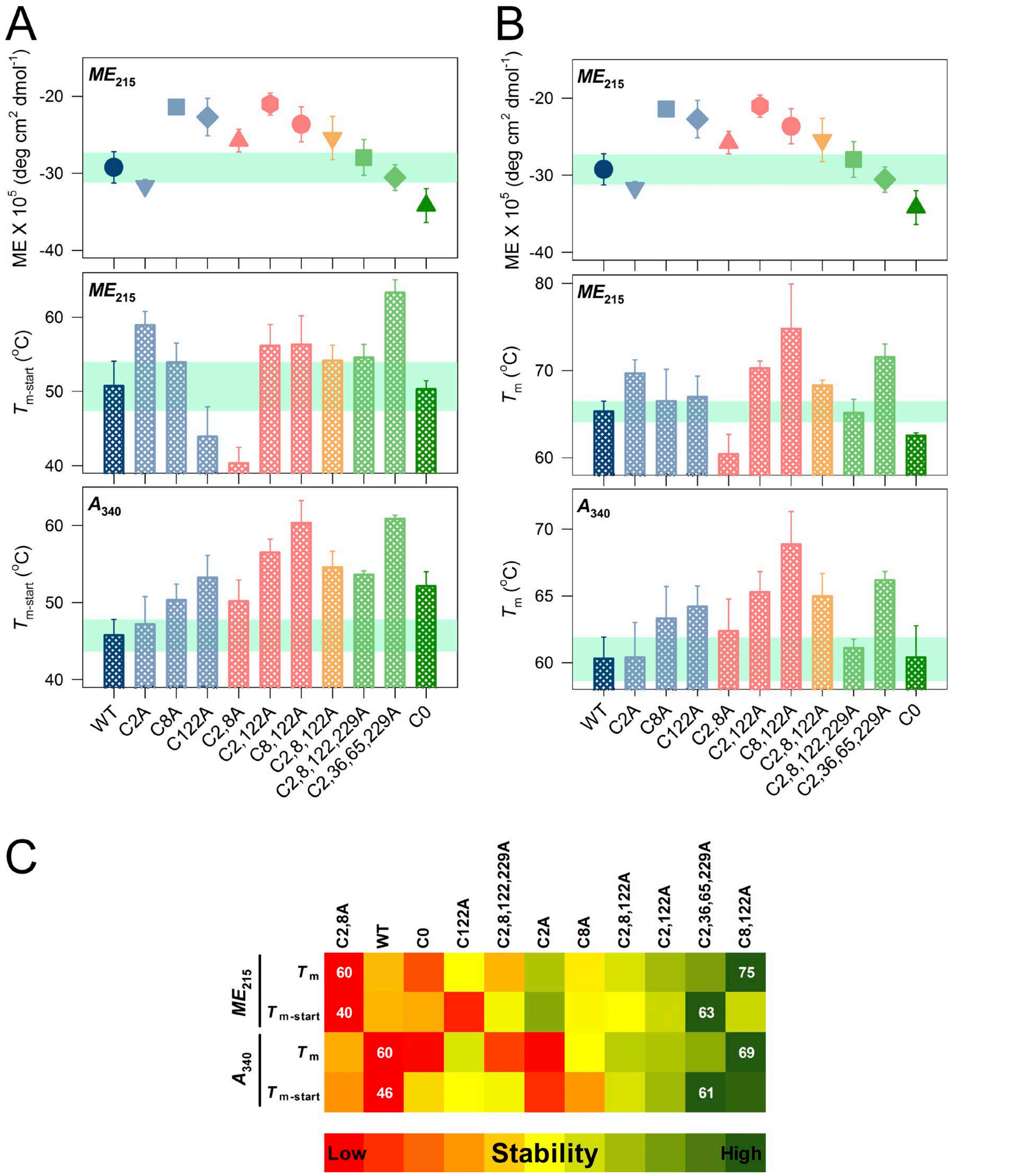

Figure S2. **Stability of hV3 is modulated by interaction of β7-β9 with the N-helix: Inference from thermal denaturation.** Continued from Fig. 5 of the main text. **(A and B)** Top: Secondary structure content of the hV3 constructs compared using molar ellipticity (ME) measured at 215 nm (ME_{215}). The ME_{215} is presented above the $T_{m\text{-start}}$ (A) and T_m (B) datasets, for easy correlation of secondary structure with the thermal parameters. A, middle and bottom: Comparison of the start temperature for thermal denaturation ($T_{m\text{-start}}$) measured using change in ME at 215 nm (middle) and absorbance at 340 nm (bottom). B, middle and bottom: Comparison of the midpoint of thermal denaturation (T_m) measured using change in ME at 215 nm (middle) and absorbance at 340 nm (bottom). A rainbow color pattern is used throughout to represent the progressive lowering in cysteine content from the hV3 WT protein (dark blue; left extreme) to the Cys-less mutant C0 (dark green; right extreme). In all plots, error bars represent SD derived from a minimum of three independent experiments. The shaded area in all plots highlights the SD obtained for WT and is presented to highlight how the thermal parameters we obtained for some mutants are significantly different from WT. Note how the A_{340} parameters (bottom panels) show an overall inverse correlation with the secondary structure content of the hV3 variant. The data are compared globally using the heat map scheme. **(C)** Global comparison of T_m and $T_{m\text{-start}}$ for hV3 variants. The stability pattern observed by different measurement was obtained and the mean was arranged in increasing order to generate the heat map for global comparison (see Global Analysis of Thermal Parameters for more details). Numbers within the heat map indicate the data range for each parameter. The data suggest that C2, C8, and C122 are important for the hV3 scaffold stability.

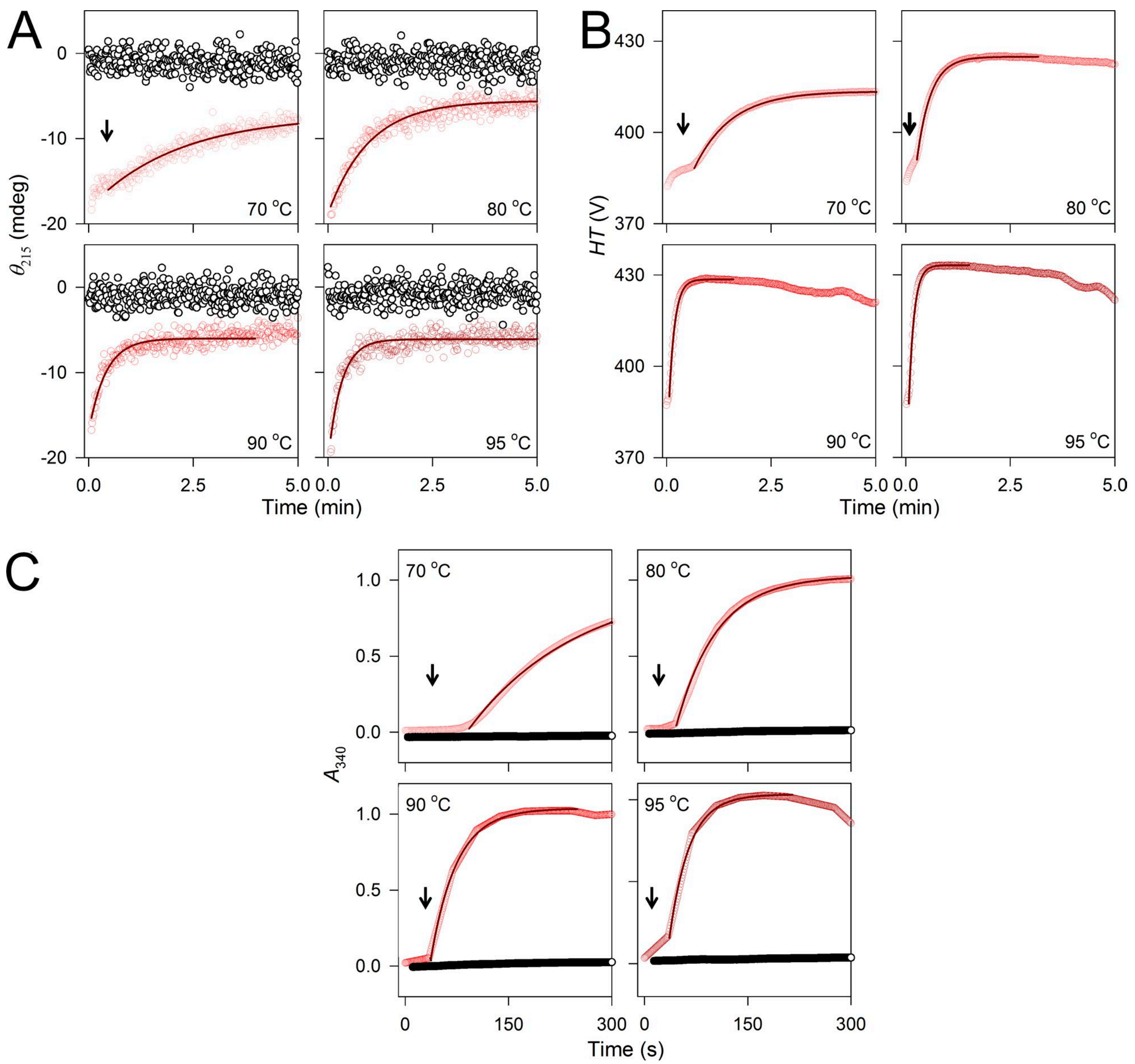

Figure S3. **Kinetics of hV3 unfolding and aggregation.** Continued from Fig. 6 of the main text. **(A–C)** Representative kinetic traces of hV3 WT unfolding and aggregation. The unfolding and aggregation events were monitored by measuring the loss of the absolute ellipticity at 215 nm (θ_{215}) using far-UV CD (A), increase in dynode voltage (HT_{215}) at 215 nm using far-UV CD (B), and increase in scattering at 340 nm (A_{340}) using UV spectroscopy (C). Shown here are data (open circles) recorded at four representative temperatures. The data for buffer containing 1% LDAO, 2 mM DTT, and 160 mM GdnHCl were recorded independently and are shown as black symbols with each kinetics trace. Here, we do not observe any change in the signal with time, suggesting that LDAO and buffer do not contribute to the measured unfolding kinetics of hV3 mutants. Data were fitted to an exponential rise function (fits are shown as solid lines) to derive the unfolding rate (k_U). Note that the lag phase (marked by an arrow) has been ignored and not considered for the analysis, and only exponential phase has been fitted to estimate k_U. When the natural logarithm of the unfolding rate ($\ln k_U$) is plotted against the inverse of temperature (1,000/T, in units of K^{-1}), the data show a linear dependence at the region corresponding to the transition temperature. The data can be fitted to a linear regression function to obtain the activation energy (E_{act}) for unfolding and aggregation (shown as a schematic in Fig. 6 A of the main text).

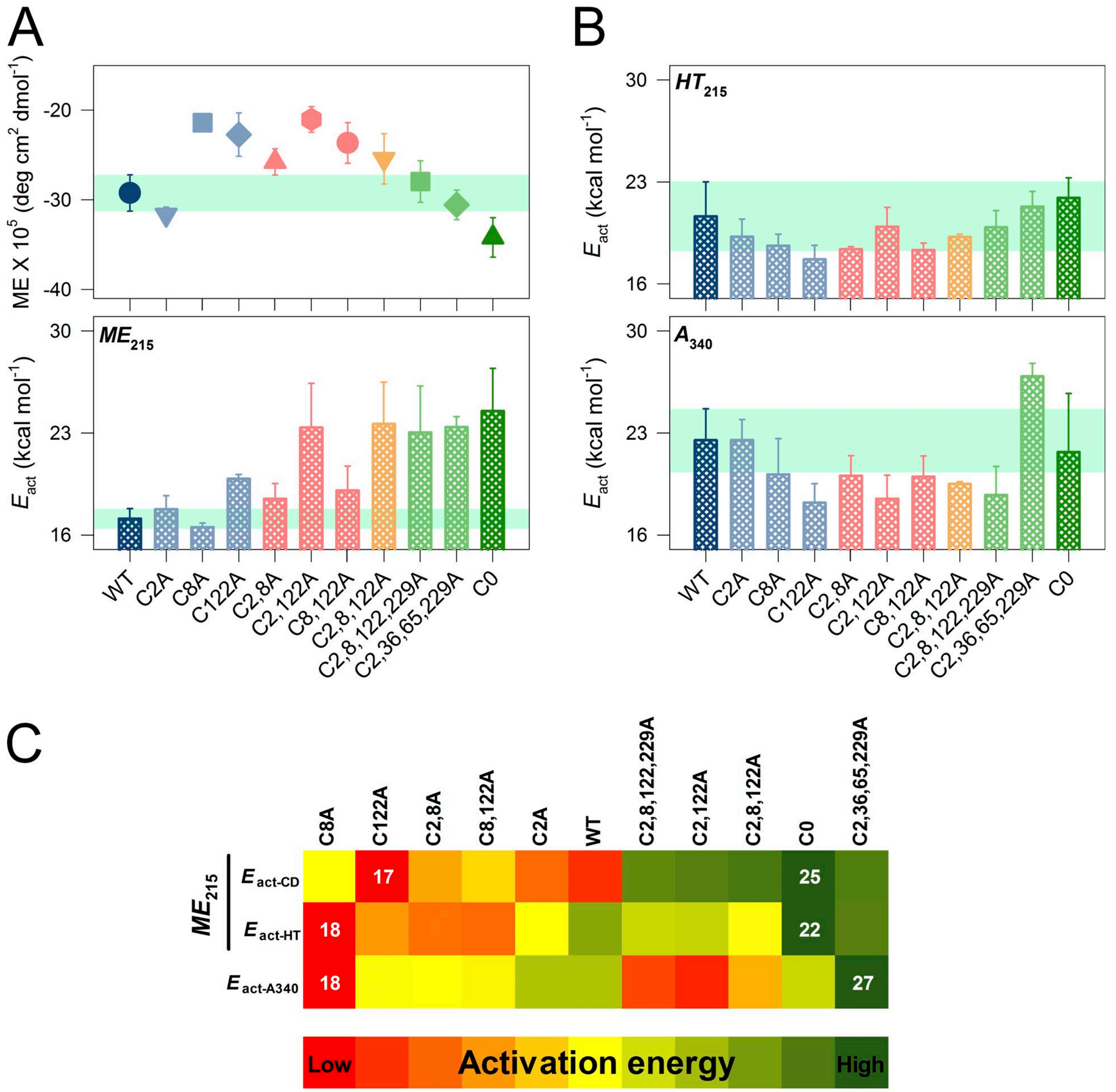

Figure S4. **Stability of hV3 is modulated by interaction of β7–β9 with the N-helix: Inference from activation energy measurements.** Continued from Fig. 6 of the main text. **(A)** Activation energy (E_{act}) of unfolding and aggregation monitored using the change in ME_{215} values (bottom) are compared with the secondary structure content (top, same as Fig. S2 A). **(B)** E_{act} of aggregation monitored independently using a change in the far-UV CD dynode voltage (HT, top) and absorbance at 340 nm (A_{340}, bottom). The individual activation energy data we obtained for each mutant is presented here as histograms. In all plots, errors are SD derived from at least two to three independent experiments. The shaded area in all plots represents the SD obtained for hV3 WT protein, and is presented to highlight how the thermal parameters we obtained for some mutants are significantly different from WT. A rainbow color pattern is used throughout to represent the progressive change in cysteine content from WT (dark blue; left extreme) to Cys-less mutant C0 (dark green; right extreme). Note how the E_{act} (unfolding + aggregation) measured using ME_{215} is considerably different across the hV3 variants compared with the WT protein. A similar difference is not clearly seen when aggregation is independently monitored (B), suggesting that a major contributor for E_{act} arises from protein unfolding. The data are compared globally using the heat map scheme. **(C)** Global comparison of E_{act} across hV3 variants, using a heat map scheme (see Global Analysis of Thermal Parameters for more details). Here, the mean of $E_{act\text{-}CD}$, $E_{act\text{-}HT}$, and $E_{act\text{-}A340}$ was arranged in increasing order for a global comparison. Numbers within the heat map indicate the data range for each parameter. In line with stability measurements in Figs. 5 and S2, the activation energy also suggests that C2,36,65,229A variant is most stabilizing due to the presence of C8, C122; and removal of C2, C8, and C122 destabilizes the barrel. Notes on $E_{act\text{-}A340}$: (a) The mean data for C2,36,65,229A mutant show an unusually high $E_{act\text{-}A340}$. We do not have a convincing explanation for this deviation. (b) We obtained a non-Arrhenius behavior for the C0 mutant upon thermal denaturation. Here, we observed two aggregation phases ($\ln k_{u\text{-}A340}$) below and above ~88°C. As a result, the rates and the associated $E_{act\text{-}A340}$ are overestimated. Such a non-Arrhenius behavior has been reported for other proteins such as lysozyme (Oliveberg et al., 1998; Matagne et al., 2000; Wang and Roberts, 2013) and is believed to arise either from the population of intermediates in the unfolding and/or aggregation pathway or the existence of an alternate pathways. In the case of hV3 C0, we find the non-Arrhenius behavior is limited to the rates obtained from A_{340} (protein aggregation) and does not affect ME_{215} (protein unfolding).

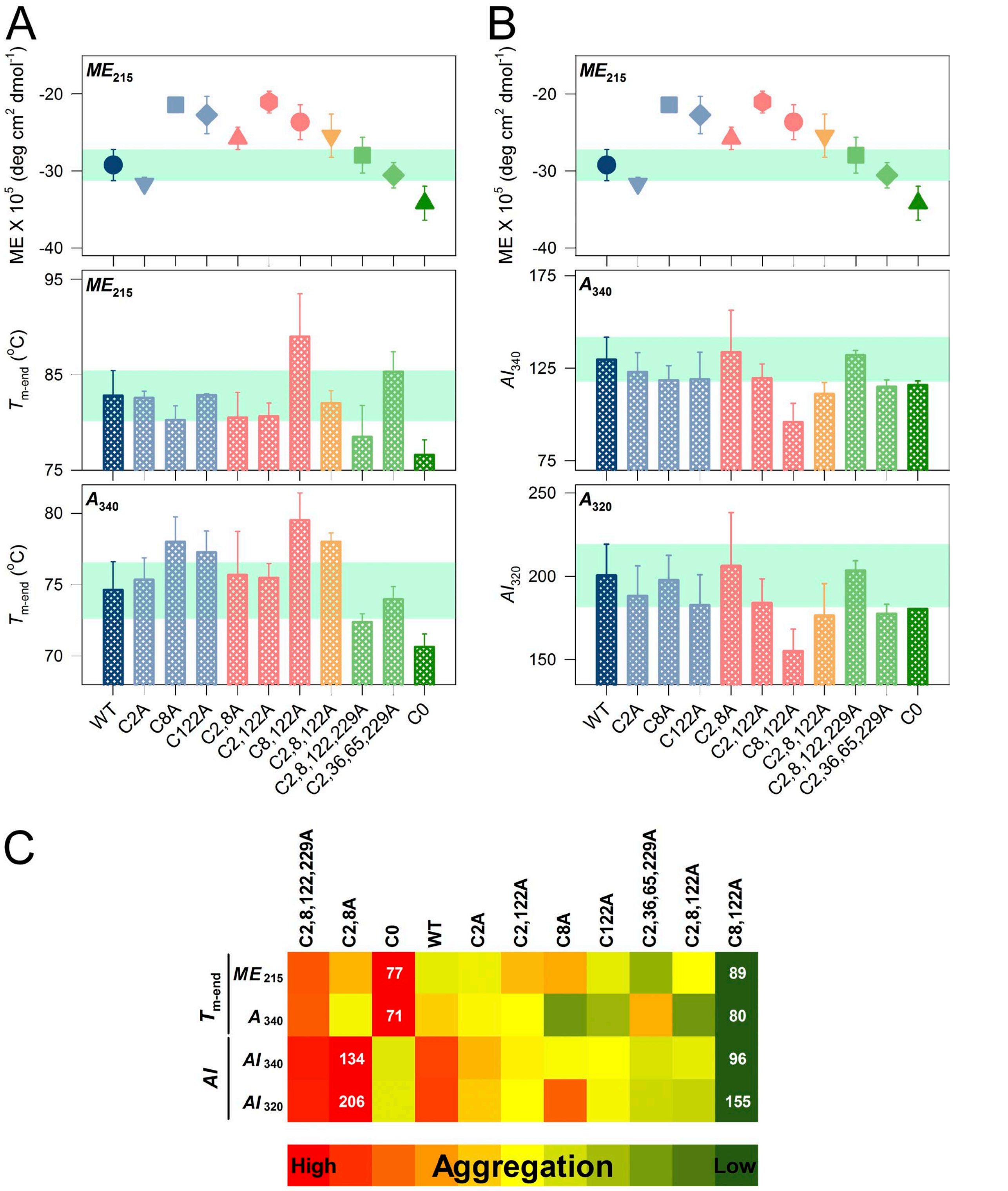

Figure S5. **Aggregation of hV3 is promoted by β1–β4: Comparison of the end-point protein aggregates after thermal denaturation. (A)** Endpoint temperature of thermal denaturation ($T_{m\text{-}end}$) monitored using far-UV CD at 215 nm (middle) is compared with the corresponding endpoint temperature obtained from scattering measurements at 340 nm (bottom). **(B)** Aggregation index calculated at 340 nm (AI_{340}, middle) and 320 nm (AI_{320}, bottom) after heating the folded hV3 variants at 95°C for 10 min. The secondary structure content of the folded protein at 215 nm is also provided in both A and B (ME_{215}, top panels), for comparison. In all plots, errors are SD derived from a minimum of two to three independent experiments. The shaded area in all plots represents the SD obtained for hV3 WT protein, and is presented to highlight how the thermal parameters we obtained for some mutants are significantly different from WT. A rainbow color pattern is used throughout to represent the progressive change in cysteine content from WT (dark blue; left extreme) to the Cys-less mutant C0 (dark green; right extreme). The data are compared globally using the heat map scheme. **(C)** Global comparison of $T_{m\text{-}end}$ and *AI*. Numbers in the heat map show the data range for each parameter. We observe that the stabilizing variants C8,122A and C2,36,65,229A from Figs. 5 and 6 show lesser aggregation propensity. Presence of C2, C8, and C122 is deleterious for the barrel and presence of C36 and C65 promotes the hV3 aggregation.

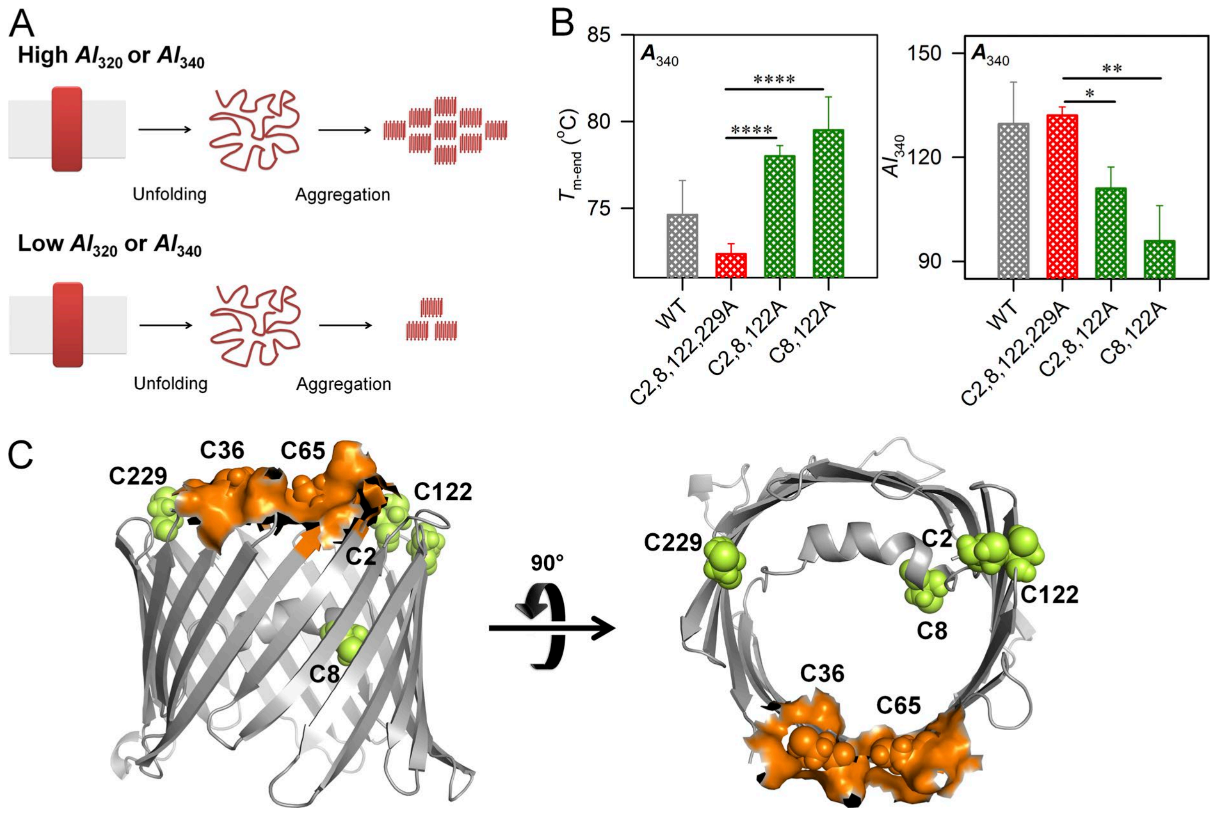

Figure S6. **Aggregation of hV3 is also promoted by β1–β4.** Continued from Fig. S5. **(A)** Schematic correlating the measured *AI* and aggregation propensity. Here, the higher value of AI_{320} or AI_{340} is suggestive of formation of large aggregates, whereas a low value indicates marginal aggregation. **(B)** Comparison of $T_{m\text{-end}}$ and *AI* calculated from A_{340} measurement. The data are compared globally using the heat map scheme in Fig. S5 C. Here, the most stabilizing and destabilizing mutants are compared on the basis of global comparison (Fig. S5 C). We observe that the stabilizing variants from Figs. 5 and 6 also show lesser aggregation propensity. Overall, presence of C2, C8, and C122 is deleterious for the barrel and presence of C36 and C65 promotes the hV3 aggregation (see Fig. S5 C for details). In all plots, error bars represent SD derived from a minimum of two to three independent experiments. Statistical significance (****, $P < 0.0001$; ***, $P < 0.001$; **, $P < 0.01$; *, $P < 0.05$) was derived using *t* test (unpaired). **(C)** The aggregation-prone region (orange) of hV3 is shown on the cartoon representation of hV3. The hV3 shows high aggregation tendency when C36–C65 (β1–β4; orange) are retained. This site represents the potential aggregation surface for hV3 aggregation.

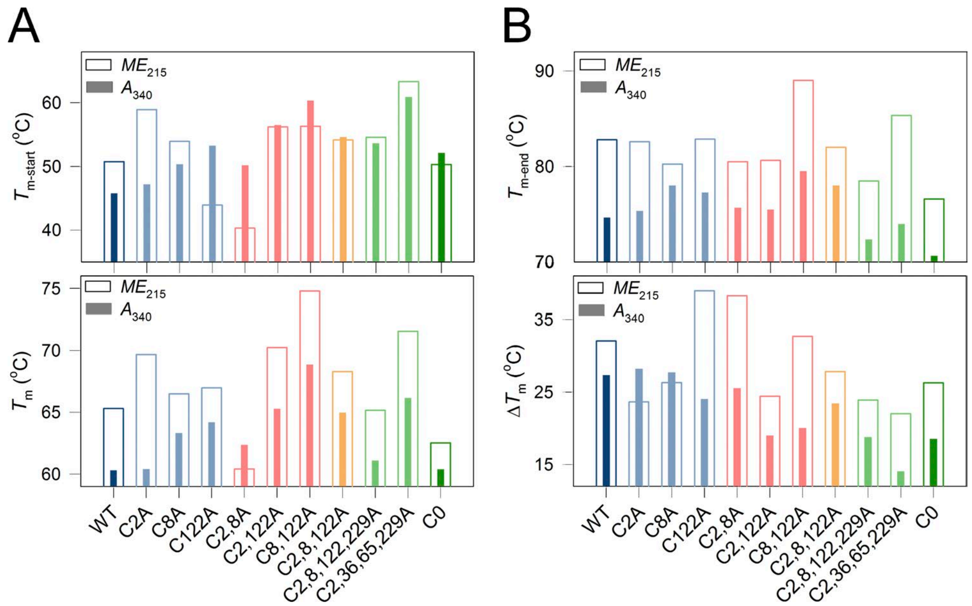

Figure S7. **Deducing the mechanism of hV3 aggregation: Comparison of thermal parameters from barrel unfolding and aggregation.** Continued from Fig. 7 of the main text. **(A and B)** Comparison of the thermal parameters $T_{\text{m-start}}$ (A, top), T_m (A, bottom), $T_{\text{m-end}}$ (B, top), and ΔT_m (B, bottom) derived from far-UV CD measurements at 215 nm (ME_{215}, open histograms) and scattering (A_{340}, filled histograms) measurements. A rainbow color pattern is used throughout to represent the progressive change in cysteine content from WT (dark blue; left extreme) to the Cys-less mutant C0 (dark green; right extreme). The data are a mean of at least two to three independent experiments. Error bars are hidden for clarity. Note how the process of unfolding and aggregation commence at similar temperatures (similar $T_{\text{m-start}}$ values) for the various hV3 variants (A, top). However, the process of aggregation is completed at much lower temperatures than that of complete protein unfolding. This is evident from the other three panels, where T_m, ΔT_m, and $T_{\text{m-end}}$ derived from A_{340} measurements are considerably lower than the ME_{215} measurements. Hence, in hV3, the process of aggregation is driven by the association of structured to partially structured protein oligomeric states. This may explain how hV3, under stress condition, forms ordered assemblies (dimmers and higher-order oligomers) without losing secondary structure. Under conditions of irreversible cellular damage due to ROS or accumulation of Aβ peptide, macromolecular protein assemblies of VDACs are formed, which may be harmful for the cell.

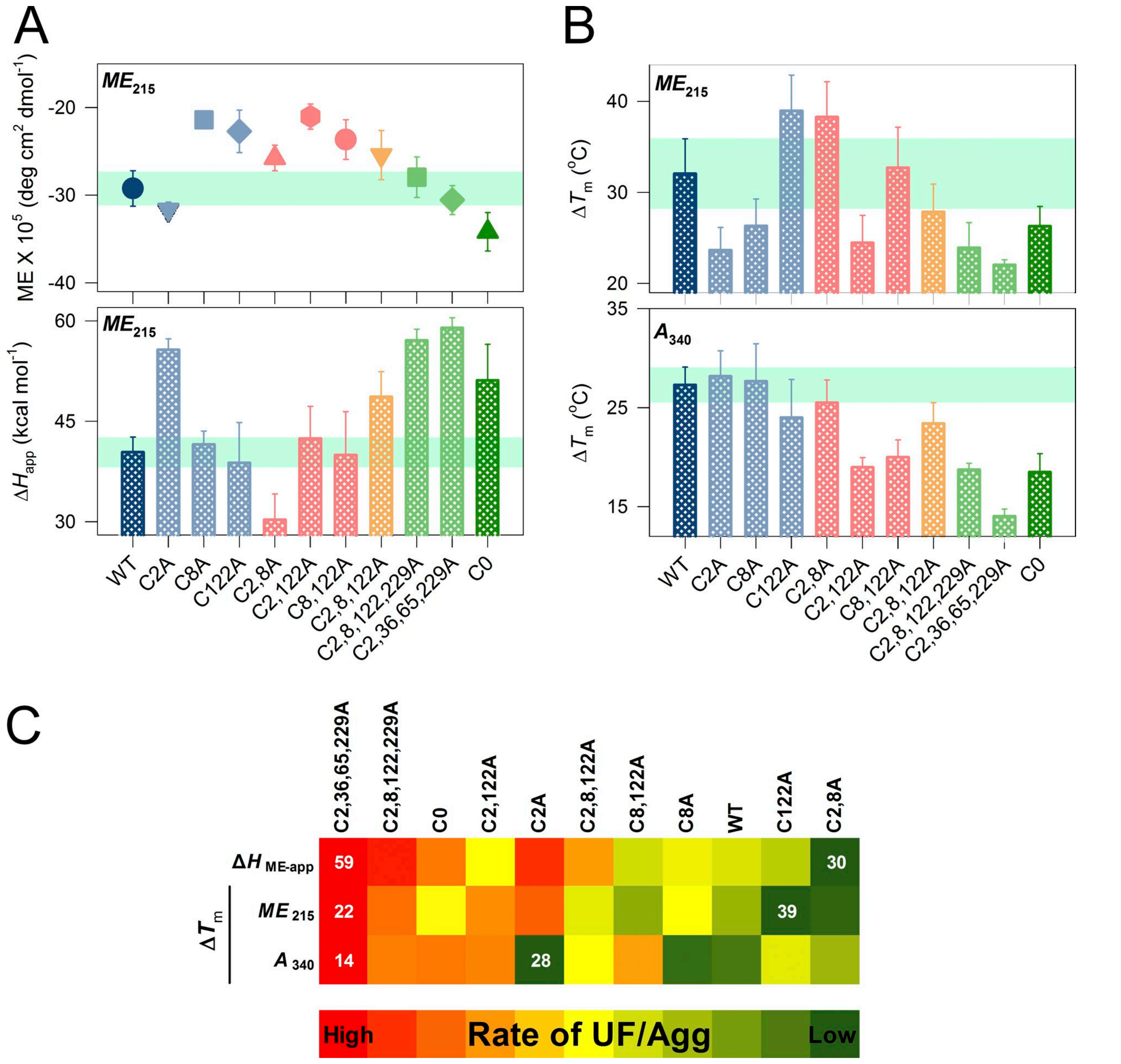

Figure S8. **Deducing the mechanism of hV3 aggregation: Comparison of unfolding and aggregation cooperativity.** Continued from Fig. 8 of the main text. **(A)** The apparent enthalpy of unfolding and aggregation ($\Delta H_{ME\text{-}app}$; bottom) measured across the various hV3 variants using far-UV CD at 215 nm is shown. The secondary structure content at 215 nm is summarized (top) for comparison. Largely, the measured apparent enthalpy correlates directly with the secondary structure content of the hV3 variant. **(B)** Cooperativity of the coupled unfolding and aggregation process is measured as $\Delta T_{m\text{-}ME} = T_{m\text{-}end} - T_{m\text{-}start}$, using far-UV CD thermal denaturation at 215 nm (top). The corresponding cooperativity of aggregation determined using thermal denaturation monitored using scattering measurements at 340 nm is also summarized for the mutants (bottom). A high value for ΔT_m indicates that unfolding ($\Delta T_{m\text{-}ME215}$) and aggregation ($\Delta T_{m\text{-}A340}$) is a slow event, and the process occurs over a larger temperature range of up to 40°C (schematic in Fig. 8). A low ΔT_m indicates that unfolding ($\Delta T_{m\text{-}ME215}$) and aggregation ($\Delta T_{m\text{-}A340}$) are highly cooperative events that are completed as soon as the temperature is incremented even by ~15°C. In all plots, errors are SD derived from a minimum of two to three independent experiments. The shaded area in all plots represents the SD obtained for hV3 WT protein, and is presented to highlight how the thermal parameters we obtained for some mutants are significantly different from WT. A rainbow color pattern is used throughout to represent the progressive change in cysteine content from WT (dark blue; left extreme) to the Cys-less mutant C0 (dark green; right extreme). The data are compared globally using the heat map scheme. **(C)** Global comparison of the unfolding cooperativity across the hV3 variants. Here, the global analysis was done using mean values from $\Delta H_{ME\text{-}app}$ and ΔT_m calculated from ME_{215} and A_{340}. Numbers in the heat map show the data range for each parameter. The most stabilizing mutants exhibit a high rate of unfolding (C2,36,65,229A mutant), suggesting that once the stabilizing interaction is disrupted the hV3 variants show rapid unfolding or aggregation. This again reaffirms the importance of α1–β7–β9 tripartite interaction for barrel stabilization and resistance to aggregation. Note how the $\Delta T_{m\text{-}ME}$ (B, top) correlates inversely with both the $\Delta H_{ME\text{-}app}$ and secondary structure content (ME_{215}) of the hV3 barrel. Hence, the unfolding cooperativity and corresponding enthalpy change depend on the structure and stability of folded hV3. Also note how the $\Delta T_{m\text{-}A340}$ is proportionately reduced with the cysteine content of hV3 (bottom), suggesting that aggregation in hV3 is not driven by the formation of nonspecific disulfide bonds when the protein unfolds.

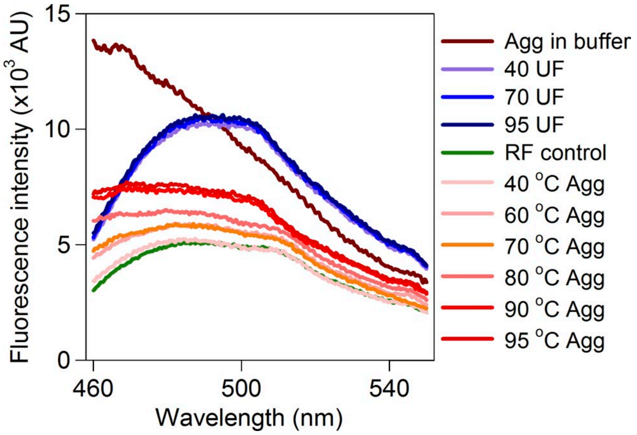

Figure S9. **Heat-induced hV3 aggregates show modest binding to ThT dye.** Representative fluorescence spectra of heat-induced hV3 protein aggregates generated by incubating the folded protein at various temperatures from 40°C to 95°C (light pink to dark red solid lines), for 10 min. Also shown are hV3 protein control samples that were prepared by three different methods: (a) aggregated hV3 WT in buffer (dark brown line); (b) unfolded (UF) protein in ∼5.0 M GdnHCl containing 1% LDAO heated at various temperatures for 10 min (light blue to dark blue lines); and (c) folded protein control in 1× buffer containing 1% LDAO (dark green color). To prepare the unfolded proteins (UF; control sample (b)), the protein samples folded in 1% LDAO were first heat-denatured (as described above) at various temperatures. These aggregates were then dissolved in ∼5.0 M GdnHCl. Control sample (a) should exhibit high ThT binding, while the ThT fluorescence for control samples (b) and (c) is expected to be low. Each dataset shown here was corrected for contributions from buffer, LDAO, and DTT. In ThT experiments with hV3, the aggregated protein shows a blue-shifted fluorescence emission spectrum (with an emission maximum at wavelengths shorter than ∼480 nm) compared with the UF protein (broad emission spectrum centered at ∼500 nm). The data indicate that ThT binds to the hV3 temperature-induced aggregates. However, the ThT fluorescence intensity is lower than those observed for other membrane protein aggregates (unpublished results), suggesting that hV3 retains only a modest β-sheet structure upon unfolding and aggregation.

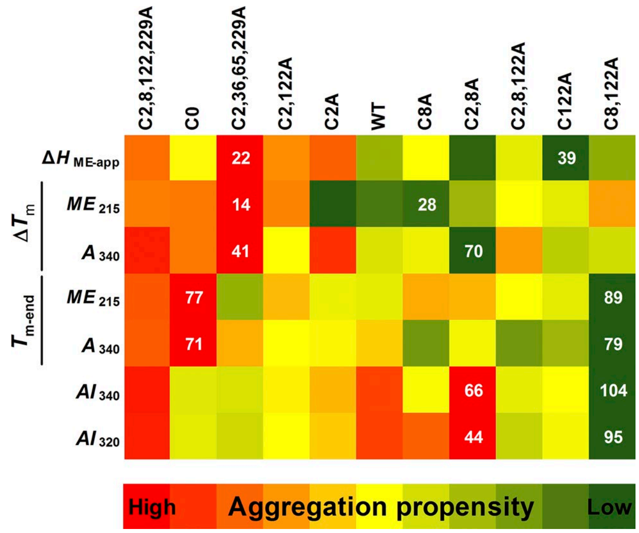

Figure S10. **Deducing the mechanism of hV3 aggregation: Global comparison of aggregation propensity of various hV3 variants.** Continued from Fig. 8 of the main text and Figs. S5, S6, S7, and S8. Global comparison of ΔH_{app}, ΔT_m, $T_{m\text{-}end}$, and *AI* calculated from ME_{215} and A_{340}. Numbers in the heat map show the data range for each parameter. hV3 shows the highest aggregation propensity when only C36 and C65 are present in the barrel (C2,8,122,229A mutant; extreme left). Note how the mutation of C122 considerably lowers aggregation of hV3 (all three mutants C2,8,122A; C122A; C8,122A on the extreme right lack C122), supporting that nucleation of aggregation is likely to occur through strands β7–β9, in the vicinity of C122.

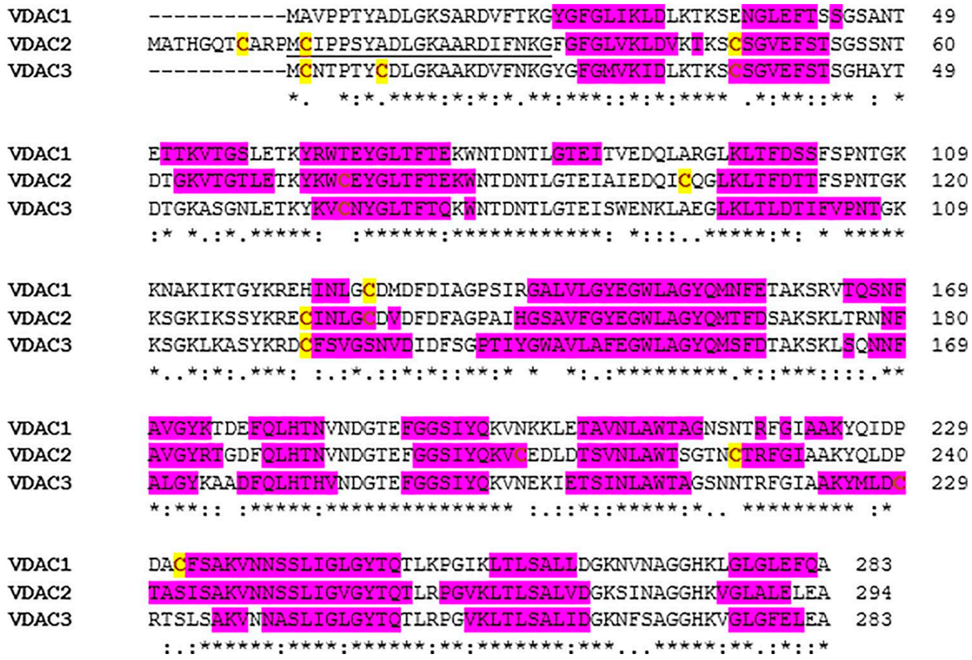

Figure S11. **Predicted aggregation zones compared across the VDAC isoforms.** Multiple sequence alignment of full-length (mature) hV1, hV2, and hV3 proteins is shown. Cysteines in the protein sequence are highlighted in red (font attribute) and yellow (background). The sequence of the N-terminal α1 helix is underlined. Note that hV2 has an extended α1 helix, due to the presence of an additional 11 residues at the N terminus. The aggregation loci are predicted using in silico analysis from 20 tools. Here, the amino acids with aggregation tendency score of ≥20% have been highlighted in pink. These consensus aggregation-prone sequences are also mapped on the modeled structure of all the VDACs (shown in Fig. S12). Note that all three VDACs show similar aggregation-prone regions. The in silico analysis suggests that β4 and β9 are aggregation prone segments on hV3 structure. The detailed in silico analysis is presented in Fig. S13 (A–C).

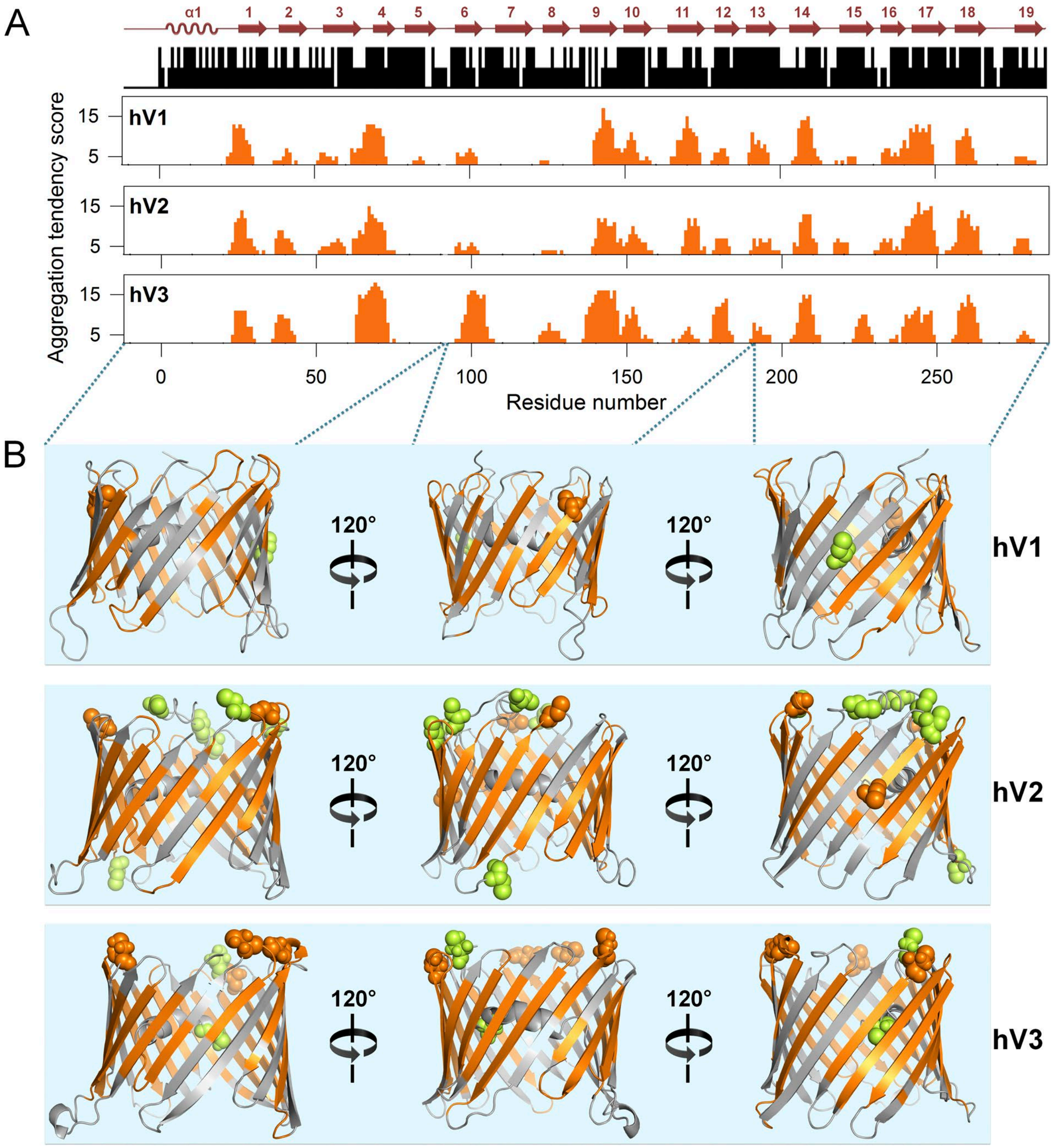

Figure S12. **Mapping aggregation zones of human VDACs in silico. (A)** Aggregation loci of VDACs derived from in silico analysis from 20 tools. Aggregation tendency scores ≥20% are plotted for all the isoforms. See Figs. S11 and S13 for the complete analysis. Shown above the scores are the consensus regions (as black histograms) from the multiple sequence alignment of hVDAC1 (hV1), hVDAC2 (hV2), and hVDAC3 (hV3). The secondary structures adopted by the various regions are shown as cartoon representation (dark red). The N-terminal helix is marked as α1 (squiggle), and the 19 β-strands (thick arrows) of VDAC are numbered. Loops and turns are shown as lines. The additional 11 residues present at the N terminus of only hV2 is also shown as a line before α1. **(B)** Aggregation loci are mapped on the structures of the three human VDAC isoforms in orange color. Cysteines are rendered as spheres (see Fig. 2 A for cysteine annotation); those cysteines present in the predicted aggregation zones are shown as orange.

Figure S13 is provided as a separate PDF file.

Table S1. **Involvement of human VDACs in various neurodegenerative diseases.**

S. No.	VDAC isoform	Interaction partner	Disease	Reference
1	VDAC1	Transactive response DNA-binding protein (TDP-43)	AD and mitophagy	Davis et al., 2018
2	VDAC1<br>VDAC2<br>VDAC3	Aβ peptide	AD and VDAC mediated aggregation	Boulbrima et al., 2016
3	VDAC1	Aβ peptide and phosphorylated tau	AD	Reddy, 2013b
4	VDAC1	Aβ peptide	AD	Smilansky et al., 2015
5	VDAC1	Glycogen synthase kinase 3 (GSK3)	AD	Reddy, 2013a
6	VDAC1	Aβ peptide and phosphorylated tau	AD	Manczak and Reddy, 2012
7	VDAC1	Hexokinases, glucokinase, and creatine kinase	Cancer and neurodegeneration	Magrì et al., 2018
8	VDAC1	Aβ peptide and hexokinases	AD	Cuadrado-Tejedor et al., 2011
9	VDAC1	Amyloid precursor protein (APP) and Aβ peptide	AD	Fernandez-Echevarria et al., 2014
10	VDAC1	B-cell lymphoma 2 (Bcl-2), Bcl-xL, and hexokinase	Cancer	Shoshan-Barmatz et al., 2017b
11	VDAC1	Parkin	Mitochondrial autophagy	Sun et al., 2012
12	VDAC1	VDAC1	Apoptosis	Shoshan-Barmatz et al., 2017a
13	VDAC1	Aβ peptide, tau, and α-synuclein	AD and PD	Magri and Messina, 2017
14	VDAC1	Aβ peptide	AD	Thinnes, 2011
15	VDAC1	Superoxide dismutase (SOD1)	Amyotrophic lateral sclerosis	Israelson et al., 2010
16	VDAC1	α-Synuclein	PD	Rostovtseva et al., 2015
17	VDAC1	TATA-box binding protein (TBP)	Neuronal cell death	Ghosh et al., 2007
18	VDAC1	α-Synuclein	PD	Chu et al., 2014
19	VDAC1	PINK1 and Parkin	PD and Mitochondrial autophagy	Geisler et al., 2010

References

Bergdoll, L.A., M.T. Lerch, J.W. Patrick, K. Belardo, C. Altenbach, P. Bisignano, A. Laganowsky, M. Grabe, W.L. Hubbell, and J. Abramson. 2018. Protonation state of glutamate 73 regulates the formation of a specific dimeric association of mVDAC1. *Proc. Natl. Acad. Sci. USA*. 115:E172–E179. https://doi.org/10.1073/pnas.1715464115

Betaneli, V., E.P. Petrov, and P. Schwille. 2012. The role of lipids in VDAC oligomerization. *Biophys. J.* 102:523–531. https://doi.org/10.1016/j.bpj.2011.12.049

Boulbrima, A., D. Temple, and G. Psakis. 2016. The multiple assemblies of VDAC: from conformational heterogeneity to β-aggregation and amyloid formation. *Biochem. Soc. Trans.* 44:1531–1540. https://doi.org/10.1042/BST20160114

Chu, Y., J.G. Goldman, L. Kelly, Y. He, T. Waliczek, and J.H. Kordower. 2014. Abnormal alpha-synuclein reduces nigral voltage-dependent anion channel 1 in sporadic and experimental Parkinson's disease. *Neurobiol. Dis.* 69:1–14. https://doi.org/10.1016/j.nbd.2014.05.003

Conchillo-Solé, O., N.S. de Groot, F.X. Avilés, J. Vendrell, X. Daura, and S. Ventura. 2007. AGGRESCAN: a server for the prediction and evaluation of "hot spots" of aggregation in polypeptides. *BMC Bioinformatics*. 8:65. https://doi.org/10.1186/1471-2105-8-65

Cuadrado-Tejedor, M., M. Vilariño, F. Cabodevilla, J. Del Río, D. Frechilla, and A. Pérez-Mediavilla. 2011. Enhanced expression of the voltage-dependent anion channel 1 (VDAC1) in Alzheimer's disease transgenic mice: an insight into the pathogenic effects of amyloid-β. *J. Alzheimers Dis.* 23:195–206. https://doi.org/10.3233/JAD-2010-100966

Davis, S.A., S. Itaman, C.M. Khalid-Janney, J.A. Sherard, J.A. Dowell, N.J. Cairns, and M.A. Gitcho. 2018. TDP-43 interacts with mitochondrial proteins critical for mitophagy and mitochondrial dynamics. *Neurosci. Lett.* 678:8–15. https://doi.org/10.1016/j.neulet.2018.04.053

DuBay, K.F., A.P. Pawar, F. Chiti, J. Zurdo, C.M. Dobson, and M. Vendruscolo. 2004. Prediction of the absolute aggregation rates of amyloidogenic polypeptide chains. *J. Mol. Biol.* 341:1317–1326. https://doi.org/10.1016/j.jmb.2004.06.043

Fernandez-Echevarria, C., M. Díaz, I. Ferrer, A. Canerina-Amaro, and R. Marin. 2014. Aβ promotes VDAC1 channel dephosphorylation in neuronal lipid rafts. Relevance to the mechanisms of neurotoxicity in Alzheimer's disease. *Neuroscience*. 278:354–366. https://doi.org/10.1016/j.neuroscience.2014.07.079

Fernandez-Escamilla, A.M., F. Rousseau, J. Schymkowitz, and L. Serrano. 2004. Prediction of sequence-dependent and mutational effects on the aggregation of peptides and proteins. *Nat. Biotechnol.* 22:1302–1306. https://doi.org/10.1038/nbt1012

Frousios, K.K., V.A. Iconomidou, C.M. Karletidi, and S.J. Hamodrakas. 2009. Amyloidogenic determinants are usually not buried. *BMC Struct. Biol.* 9:44. https://doi.org/10.1186/1472-6807-9-44

Galzitskaya, O.V., S.O. Garbuzynskiy, and M.Y. Lobanov. 2006. Prediction of amyloidogenic and disordered regions in protein chains. *PLOS Comput. Biol.* 2:e177. https://doi.org/10.1371/journal.pcbi.0020177

Garbuzynskiy, S.O., M.Y. Lobanov, and O.V. Galzitskaya. 2010. FoldAmyloid: a method of prediction of amyloidogenic regions from protein sequence. *Bioinformatics*. 26:326–332. https://doi.org/10.1093/bioinformatics/btp691

Gasior, P., and M. Kotulska. 2014. FISH Amyloid - a new method for finding amyloidogenic segments in proteins based on site specific co-occurrence of aminoacids. *BMC Bioinformatics*. 15:54. https://doi.org/10.1186/1471-2105-15-54

Geisler, S., K.M. Holmström, D. Skujat, F.C. Fiesel, O.C. Rothfuss, P.J. Kahle, and W. Springer. 2010. PINK1/Parkin-mediated mitophagy is dependent on VDAC1 and p62/SQSTM1. *Nat. Cell Biol.* 12:119–131. https://doi.org/10.1038/ncb2012
Ghosh, T., N. Pandey, A. Maitra, S.K. Brahmachari, and B. Pillai. 2007. A role for voltage-dependent anion channel Vdac1 in polyglutamine-mediated neuronal cell death. *PLoS One.* 2:e1170. https://doi.org/10.1371/journal.pone.0001170
Hamodrakas, S.J., C. Liappa, and V.A. Iconomidou. 2007. Consensus prediction of amyloidogenic determinants in amyloid fibril-forming proteins. *Int. J. Biol. Macromol.* 41:295–300. https://doi.org/10.1016/j.ijbiomac.2007.03.008
Hoogenboom, B.W., K. Suda, A. Engel, and D. Fotiadis. 2007. The supramolecular assemblies of voltage-dependent anion channels in the native membrane. *J. Mol. Biol.* 370:246–255. https://doi.org/10.1016/j.jmb.2007.04.073
Israelson, A., N. Arbel, S. Da Cruz, H. Ilieva, K. Yamanaka, V. Shoshan-Barmatz, and D.W. Cleveland. 2010. Misfolded mutant SOD1 directly inhibits VDAC1 conductance in a mouse model of inherited ALS. *Neuron.* 67:575–587. https://doi.org/10.1016/j.neuron.2010.07.019
Kim, C., J. Choi, S.J. Lee, W.J. Welsh, and S. Yoon. 2009. NetCSSP: web application for predicting chameleon sequences and amyloid fibril formation. *Nucleic Acids Res.* 37(Web Server):W469–W473. https://doi.org/10.1093/nar/gkp351
López de la Paz, M., and L. Serrano. 2004. Sequence determinants of amyloid fibril formation. *Proc. Natl. Acad. Sci. USA.* 101:87–92. https://doi.org/10.1073/pnas.2634884100
Magri, A., and A. Messina. 2017. Interactions of VDAC with Proteins Involved in Neurodegenerative Aggregation: An Opportunity for Advancement on Therapeutic Molecules. *Curr. Med. Chem.* 24:4470–4487.
Magrì, A., S. Reina, and V. De Pinto. 2018. VDAC1 as Pharmacological Target in Cancer and Neurodegeneration: Focus on Its Role in Apoptosis. *Front Chem.* 6:108. https://doi.org/10.3389/fchem.2018.00108
Manczak, M., and P.H. Reddy. 2012. Abnormal interaction of VDAC1 with amyloid beta and phosphorylated tau causes mitochondrial dysfunction in Alzheimer's disease. *Hum. Mol. Genet.* 21:5131–5146. https://doi.org/10.1093/hmg/dds360
Matagne, A., M. Jamin, E.W. Chung, C.V. Robinson, S.E. Radford, and C.M. Dobson. 2000. Thermal unfolding of an intermediate is associated with non-Arrhenius kinetics in the folding of hen lysozyme. *J. Mol. Biol.* 297:193–210. https://doi.org/10.1006/jmbi.2000.3540
Maurer-Stroh, S., M. Debulpaep, N. Kuemmerer, M. Lopez de la Paz, I.C. Martins, J. Reumers, K.L. Morris, A. Copland, L. Serpell, L. Serrano, et al. 2010. Exploring the sequence determinants of amyloid structure using position-specific scoring matrices. *Nat. Methods.* 7:237–242. https://doi.org/10.1038/nmeth.1432
O'Donnell, C.W., J. Waldispühl, M. Lis, R. Halfmann, S. Devadas, S. Lindquist, and B. Berger. 2011. A method for probing the mutational landscape of amyloid structure. *Bioinformatics.* 27:i34–i42. https://doi.org/10.1093/bioinformatics/btr238
Oliveberg, M., Y.J. Tan, M. Silow, and A.R. Fersht. 1998. The changing nature of the protein folding transition state: implications for the shape of the free-energy profile for folding. *J. Mol. Biol.* 277:933–943. https://doi.org/10.1006/jmbi.1997.1612
Pawar, A.P., K.F. Dubay, J. Zurdo, F. Chiti, M. Vendruscolo, and C.M. Dobson. 2005. Prediction of "aggregation-prone" and "aggregation-susceptible" regions in proteins associated with neurodegenerative diseases. *J. Mol. Biol.* 350:379–392. https://doi.org/10.1016/j.jmb.2005.04.016
Pawlicki, S., A. Le Béchec, and C. Delamarche. 2008. AMYPdb: a database dedicated to amyloid precursor proteins. *BMC Bioinformatics.* 9:273. https://doi.org/10.1186/1471-2105-9-273
Raschle, T., S. Hiller, T.Y. Yu, A.J. Rice, T. Walz, and G. Wagner. 2009. Structural and functional characterization of the integral membrane protein VDAC-1 in lipid bilayer nanodiscs. *J. Am. Chem. Soc.* 131:17777–17779. https://doi.org/10.1021/ja907918r
Reddy, P.H. 2013a. Amyloid beta-induced glycogen synthase kinase 3β phosphorylated VDAC1 in Alzheimer's disease: implications for synaptic dysfunction and neuronal damage. *Biochim. Biophys. Acta.* 1832:1913–1921. https://doi.org/10.1016/j.bbadis.2013.06.012
Reddy, P.H. 2013b. Is the mitochondrial outermembrane protein VDAC1 therapeutic target for Alzheimer's disease? *Biochim. Biophys. Acta.* 1832:67–75. https://doi.org/10.1016/j.bbadis.2012.09.003
Rostovtseva, T.K., P.A. Gurnev, O. Protchenko, D.P. Hoogerheide, T.L. Yap, C.C. Philpott, J.C. Lee, and S.M. Bezrukov. 2015. α-Synuclein Shows High Affinity Interaction with Voltage-dependent Anion Channel, Suggesting Mechanisms of Mitochondrial Regulation and Toxicity in Parkinson Disease. *J. Biol. Chem.* 290:18467–18477. https://doi.org/10.1074/jbc.M115.641746
Schredelseker, J., A. Paz, C.J. López, C. Altenbach, C.S. Leung, M.K. Drexler, J.N. Chen, W.L. Hubbell, and J. Abramson. 2014. High resolution structure and double electron-electron resonance of the zebrafish voltage-dependent anion channel 2 reveal an oligomeric population. *J. Biol. Chem.* 289:12566–12577. https://doi.org/10.1074/jbc.M113.497438
Shoshan-Barmatz, V., Y. Krelin, and Q. Chen. 2017a. VDAC1 as a Player in Mitochondria-Mediated Apoptosis and Target for Modulating Apoptosis. *Curr. Med. Chem.* 24:4435–4446.
Shoshan-Barmatz, V., Y. Krelin, A. Shteinfer-Kuzmine, and T. Arif. 2017b. Voltage-Dependent Anion Channel 1 As an Emerging Drug Target for Novel Anti-Cancer Therapeutics. *Front. Oncol.* 7:154. https://doi.org/10.3389/fonc.2017.00154
Smilansky, A., L. Dangoor, I. Nakdimon, D. Ben-Hail, D. Mizrachi, and V. Shoshan-Barmatz. 2015. The Voltage-dependent Anion Channel 1 Mediates Amyloid β Toxicity and Represents a Potential Target for Alzheimer Disease Therapy. *J. Biol. Chem.* 290:30670–30683. https://doi.org/10.1074/jbc.M115.691493
Sormanni, P., F.A. Aprile, and M. Vendruscolo. 2015. The CamSol method of rational design of protein mutants with enhanced solubility. *J. Mol. Biol.* 427:478–490. https://doi.org/10.1016/j.jmb.2014.09.026
Sun, Y., A.A. Vashisht, J. Tchieu, J.A. Wohlschlegel, and L. Dreier. 2012. Voltage-dependent anion channels (VDACs) recruit Parkin to defective mitochondria to promote mitochondrial autophagy. *J. Biol. Chem.* 287:40652–40660. https://doi.org/10.1074/jbc.M112.419721
Tartaglia, G.G., and M. Vendruscolo. 2008. The Zyggregator method for predicting protein aggregation propensities. *Chem. Soc. Rev.* 37:1395–1401. https://doi.org/10.1039/b706784b
Tartaglia, G.G., A.P. Pawar, S. Campioni, C.M. Dobson, F. Chiti, and M. Vendruscolo. 2008. Prediction of aggregation-prone regions in structured proteins. *J. Mol. Biol.* 380:425–436. https://doi.org/10.1016/j.jmb.2008.05.013
Thangakani, A.M., S. Kumar, R. Nagarajan, D. Velmurugan, and M.M. Gromiha. 2014. GAP: towards almost 100 percent prediction for β-strand-mediated aggregating peptides with distinct morphologies. *Bioinformatics.* 30:1983–1990. https://doi.org/10.1093/bioinformatics/btu167
Thinnes, F.P. 2011. Apoptogenic interactions of plasmalemmal type-1 VDAC and Aβ peptides via GxxxG motifs induce Alzheimer's disease - a basic model of apoptosis? *Wien. Med. Wochenschr.* 161:274–276. https://doi.org/10.1007/s10354-011-0887-5
Tian, J., N. Wu, J. Guo, and Y. Fan. 2009. Prediction of amyloid fibril-forming segments based on a support vector machine. *BMC Bioinformatics.* 10(Suppl 1):S45. https://doi.org/10.1186/1471-2105-10-S1-S45
Tsolis, A.C., N.C. Papandreou, V.A. Iconomidou, and S.J. Hamodrakas. 2013. A consensus method for the prediction of 'aggregation-prone' peptides in globular proteins. *PLoS One.* 8:e54175. https://doi.org/10.1371/journal.pone.0054175
Walsh, I., F. Seno, S.C. Tosatto, and A. Trovato. 2014. PASTA 2.0: an improved server for protein aggregation prediction. *Nucleic Acids Res.* 42(W1):W301–W307. https://doi.org/10.1093/nar/gku399
Wang, W., and C.J. Roberts. 2013. Non-Arrhenius protein aggregation. *AAPS J.* 15:840–851. https://doi.org/10.1208/s12248-013-9485-3
Yu, T.Y., T. Raschle, S. Hiller, and G. Wagner. 2012. Solution NMR spectroscopic characterization of human VDAC-2 in detergent micelles and lipid bilayer nanodiscs. *Biochim. Biophys. Acta.* 1818:1562–1569. https://doi.org/10.1016/j.bbamem.2011.11.012
Zhang, Z., H. Chen, and L. Lai. 2007. Identification of amyloid fibril-forming segments based on structure and residue-based statistical potential. *Bioinformatics.* 23:2218–2225. https://doi.org/10.1093/bioinformatics/btm325
Zibaee, S., O.S. Makin, M. Goedert, and L.C. Serpell. 2007. A simple algorithm locates beta-strands in the amyloid fibril core of alpha-synuclein, Abeta, and tau using the amino acid sequence alone. *Protein Sci.* 16:906–918. https://doi.org/10.1110/ps.062624507
