# Supplementary material for: Helix–strand interaction regulates stability and aggregation of the human mitochondrial membrane protein channel VDAC3
Source: J Gen Physiol. 2019 Jan 23;151(4):489–504. doi: 10.1085/jgp.201812272 (PMC6445588; doi:10.1085/jgp.201812272)
Supplement: Figure S13 (PDF) [file JGP_201812272_FigS13.pdf]

Figure S13. **Predicted aggregation zones from 20 in silico tools for each VDAC isoform. (A–C)** Aggregation-prone regions predicted by in silico analysis from 20 tools (listed on the left) are mapped on the sequence of hV1 (A), hV2 (B), and hV3 (C). In silico analysis was performed using default parameters, unless specified (see description in Materials and methods). Aggregation-prone residues predicted from each tool are highlighted in pink. The consensus aggregation loci (shown separately as the last line and marked as "Consensus") has been generated by selecting amino acid residues with aggregation tendency score of ≥20% across the analysis. The consensus aggregation-prone region has been compared across the three VDACs in Fig. S11 and mapped on the structures of the VDACs in Fig. S12. A.P.D.: Average Packing Density; B.S.C.: β-Strand Contiguity; H.C.E.: Hexapeptide Conformational Energy; P.C.S.: Possible secondary structure Conformational Switches (see Supplemental notes for details).

A

TANGO	MAVPPTYADL	GKSARDVFTK	GYGFGLIKLD	LKTKSENGLE	FTSSGSANTE	TTKVTGSLET	60
Waltz	MAVPPTYADL	GKSARDVFTK	GYGFGLIKLD	LKTKSENGLE	FTSSGSANTE	TTKVTGSLET	60
Aggrescan	MAVPPTYADL	GKSARDVFTK	GYGFGLIKLD	LKTKSENGLE	FTSSGSANTE	TTKVTGSLET	60
Fish amyloid	MAVPPTYADL	GKSARDVFTK	GYGFGLIKLD	LKTKSENGLE	FTSSGSANTE	TTKVTGSLET	60
Zyggregator	MAVPPTYADL	GKSARDVFTK	GYGFGLIKLD	LKTKSENGLE	FTSSGSANTE	TTKVTGSLET	60
Fold amyloid	MAVPPTYADL	GKSARDVFTK	GYGFGLIKLD	LKTKSENGLE	FTSSGSANTE	TTKVTGSLET	60
PASTA	MAVPPTYADL	GKSARDVFTK	GYGFGLIKLD	LKTKSENGLE	FTSSGSANTE	TTKVTGSLET	60
Amyl-pred	MAVPPTYADL	GKSARDVFTK	GYGFGLIKLD	LKTKSENGLE	FTSSGSANTE	TTKVTGSLET	60
Amyl-pred2	MAVPPTYADL	GKSARDVFTK	GYGFGLIKLD	LKTKSENGLE	FTSSGSANTE	TTKVTGSLET	60
GAP	MAVPPTYADL	GKSARDVFTK	GYGFGLIKLD	LKTKSENGLE	FTSSGSANTE	TTKVTGSLET	60
PaFig	MAVPPTYADL	GKSARDVFTK	GYGFGLIKLD	LKTKSENGLE	FTSSGSANTE	TTKVTGSLET	60
Amyl-mutants	MAVPPTYADL	GKSARDVFTK	GYGFGLIKLD	LKTKSENGLE	FTSSGSANTE	TTKVTGSLET	60
Amyl-patterns	MAVPPTYADL	GKSARDVFTK	GYGFGLIKLD	LKTKSENGLE	FTSSGSANTE	TTKVTGSLET	60
MetAmyl	MAVPPTYADL	GKSARDVFTK	GYGFGLIKLD	LKTKSENGLE	FTSSGSANTE	TTKVTGSLET	60
A.P.D.	MAVPPTYADL	GKSARDVFTK	GYGFGLIKLD	LKTKSENGLE	FTSSGSANTE	TTKVTGSLET	60
B.S.C.	MAVPPTYADL	GKSARDVFTK	GYGFGLIKLD	LKTKSENGLE	FTSSGSANTE	TTKVTGSLET	60
H.C.E	MAVPPTYADL	GKSARDVFTK	GYGFGLIKLD	LKTKSENGLE	FTSSGSANTE	TTKVTGSLET	60
NetCSSP	MAVPPTYADL	GKSARDVFTK	GYGFGLIKLD	LKTKSENGLE	FTSSGSANTE	TTKVTGSLET	60
P.C.S.	MAVPPTYADL	GKSARDVFTK	GYGFGLIKLD	LKTKSENGLE	FTSSGSANTE	TTKVTGSLET	60
CamSol	MAVPPTYADL	GKSARDVFTK	GYGFGLIKLD	LKTKSENGLE	FTSSGSANTE	TTKVTGSLET	60
Consensus	MAVPPTYADL	GKSARDVFTK	GYGFGLIKLD	LKTKSENGLE	FTSSGSANTE	TTKVTGSLET	60
	**********	**********	**********	**********	**********	**********	

TANGO	KYRWTEYGLT	FTEKWNTDNT	LGTEITVEDQ	LARGLKLTFD	SSFSPNTGKK	NAKIKTGYKR	120
Waltz	KYRWTEYGLT	FTEKWNTDNT	LGTEITVEDQ	LARGLKLTFD	SSFSPNTGKK	NAKIKTGYKR	120
Aggrescan	KYRWTEYGLT	FTEKWNTDNT	LGTEITVEDQ	LARGLKLTFD	SSFSPNTGKK	NAKIKTGYKR	120
Fish amyloid	KYRWTEYGLT	FTEKWNTDNT	LGTEITVEDQ	LARGLKLTFD	SSFSPNTGKK	NAKIKTGYKR	120
Zyggregator	KYRWTEYGLT	FTEKWNTDNT	LGTEITVEDQ	LARGLKLTFD	SSFSPNTGKK	NAKIKTGYKR	120
Fold amyloid	KYRWTEYGLT	FTEKWNTDNT	LGTEITVEDQ	LARGLKLTFD	SSFSPNTGKK	NAKIKTGYKR	120
PASTA	KYRWTEYGLT	FTEKWNTDNT	LGTEITVEDQ	LARGLKLTFD	SSFSPNTGKK	NAKIKTGYKR	120
Amyl-pred	KYRWTEYGLT	FTEKWNTDNT	LGTEITVEDQ	LARGLKLTFD	SSFSPNTGKK	NAKIKTGYKR	120
Amyl-pred2	KYRWTEYGLT	FTEKWNTDNT	LGTEITVEDQ	LARGLKLTFD	SSFSPNTGKK	NAKIKTGYKR	120
GAP	KYRWTEYGLT	FTEKWNTDNT	LGTEITVEDQ	LARGLKLTFD	SSFSPNTGKK	NAKIKTGYKR	120
PaFig	KYRWTEYGLT	FTEKWNTDNT	LGTEITVEDQ	LARGLKLTFD	SSFSPNTGKK	NAKIKTGYKR	120
Amyl-mutants	KYRWTEYGLT	FTEKWNTDNT	LGTEITVEDQ	LARGLKLTFD	SSFSPNTGKK	NAKIKTGYKR	120
Amyl-patterns	KYRWTEYGLT	FTEKWNTDNT	LGTEITVEDQ	LARGLKLTFD	SSFSPNTGKK	NAKIKTGYKR	120
MetAmyl	KYRWTEYGLT	FTEKWNTDNT	LGTEITVEDQ	LARGLKLTFD	SSFSPNTGKK	NAKIKTGYKR	120
A.P.D.	KYRWTEYGLT	FTEKWNTDNT	LGTEITVEDQ	LARGLKLTFD	SSFSPNTGKK	NAKIKTGYKR	120
B.S.C.	KYRWTEYGLT	FTEKWNTDNT	LGTEITVEDQ	LARGLKLTFD	SSFSPNTGKK	NAKIKTGYKR	120
H.C.E	KYRWTEYGLT	FTEKWNTDNT	LGTEITVEDQ	LARGLKLTFD	SSFSPNTGKK	NAKIKTGYKR	120
NetCSSP	KYRWTEYGLT	FTEKWNTDNT	LGTEITVEDQ	LARGLKLTFD	SSFSPNTGKK	NAKIKTGYKR	120
P.C.S.	KYRWTEYGLT	FTEKWNTDNT	LGTEITVEDQ	LARGLKLTFD	SSFSPNTGKK	NAKIKTGYKR	120
CamSol	KYRWTEYGLT	FTEKWNTDNT	LGTEITVEDQ	LARGLKLTFD	SSFSPNTGKK	NAKIKTGYKR	120
Consensus	KYRWTEYGLT	FTEKWNTDNT	LGTEITVEDQ	LARGLKLTFD	SSFSPNTGKK	NAKIKTGYKR	120
	**********	**********	**********	**********	**********	**********	

TANGO	EHINLGCDMD	FDIAGPSIRG	ALVLGYEGWL	AGYQMNFETA	KSRVTQSNFA	VGYKTDEFQL	180
Waltz	EHINLGCDMD	FDIAGPSIRG	ALVLGYEGWL	AGYQMNFETA	KSRVTQSNFA	VGYKTDEFQL	180
Aggrescan	EHINLGCDMD	FDIAGPSIRG	ALVLGYEGWL	AGYQMNFETA	KSRVTQSNFA	VGYKTDEFQL	180
Fish amyloid	EHINLGCDMD	FDIAGPSIRG	ALVLGYEGWL	AGYQMNFETA	KSRVTQSNFA	VGYKTDEFQL	180
Zyggregator	EHINLGCDMD	FDIAGPSIRG	ALVLGYEGWL	AGYQMNFETA	KSRVTQSNFA	VGYKTDEFQL	180
Fold amyloid	EHINLGCDMD	FDIAGPSIRG	ALVLGYEGWL	AGYQMNFETA	KSRVTQSNFA	VGYKTDEFQL	180
PASTA	EHINLGCDMD	FDIAGPSIRG	ALVLGYEGWL	AGYQMNFETA	KSRVTQSNFA	VGYKTDEFQL	180
Amyl-pred	EHINLGCDMD	FDIAGPSIRG	ALVLGYEGWL	AGYQMNFETA	KSRVTQSNFA	VGYKTDEFQL	180
Amyl-pred2	EHINLGCDMD	FDIAGPSIRG	ALVLGYEGWL	AGYQMNFETA	KSRVTQSNFA	VGYKTDEFQL	180
GAP	EHINLGCDMD	FDIAGPSIRG	ALVLGYEGWL	AGYQMNFETA	KSRVTQSNFA	VGYKTDEFQL	180
PaFig	EHINLGCDMD	FDIAGPSIRG	ALVLGYEGWL	AGYQMNFETA	KSRVTQSNFA	VGYKTDEFQL	180
Amyl-mutants	EHINLGCDMD	FDIAGPSIRG	ALVLGYEGWL	AGYQMNFETA	KSRVTQSNFA	VGYKTDEFQL	180
Amyl-patterns	EHINLGCDMD	FDIAGPSIRG	ALVLGYEGWL	AGYQMNFETA	KSRVTQSNFA	VGYKTDEFQL	180
MetAmyl	EHINLGCDMD	FDIAGPSIRG	ALVLGYEGWL	AGYQMNFETA	KSRVTQSNFA	VGYKTDEFQL	180
A.P.D.	EHINLGCDMD	FDIAGPSIRG	ALVLGYEGWL	AGYQMNFETA	KSRVTQSNFA	VGYKTDEFQL	180
B.S.C.	EHINLGCDMD	FDIAGPSIRG	ALVLGYEGWL	AGYQMNFETA	KSRVTQSNFA	VGYKTDEFQL	180
H.C.E	EHINLGCDMD	FDIAGPSIRG	ALVLGYEGWL	AGYQMNFETA	KSRVTQSNFA	VGYKTDEFQL	180
NetCSSP	EHINLGCDMD	FDIAGPSIRG	ALVLGYEGWL	AGYQMNFETA	KSRVTQSNFA	VGYKTDEFQL	180
P.C.S.	EHINLGCDMD	FDIAGPSIRG	ALVLGYEGWL	AGYQMNFETA	KSRVTQSNFA	VGYKTDEFQL	180
CamSol	EHINLGCDMD	FDIAGPSIRG	ALVLGYEGWL	AGYQMNFETA	KSRVTQSNFA	VGYKTDEFQL	180
Consensus	EHINLGCDMD	FDIAGPSIRG	ALVLGYEGWL	AGYQMNFETA	KSRVTQSNFA	VGYKTDEFQL	180
	**********	**********	**********	**********	**********	**********	

TANGO	HTNVNDGTEF	GGSIYQKVNK	KLETAVNLAW	TAGNSNTRFG	IAAKYQIDPD	ACFSAKVNNS	240
Waltz	HTNVNDGTEF	GGSIYQKVNK	KLETAVNLAW	TAGNSNTRFG	IAAKYQIDPD	ACFSAKVNNS	240
Aggrescan	HTNVNDGTEF	GGSIYQKVNK	KLETAVNLAW	TAGNSNTRFG	IAAKYQIDPD	ACFSAKVNNS	240
Fish amyloid	HTNVNDGTEF	GGSIYQKVNK	KLETAVNLAW	TAGNSNTRFG	IAAKYQIDPD	ACFSAKVNNS	240
Zyggregator	HTNVNDGTEF	GGSIYQKVNK	KLETAVNLAW	TAGNSNTRFG	IAAKYQIDPD	ACFSAKVNNS	240
Fold amyloid	HTNVNDGTEF	GGSIYQKVNK	KLETAVNLAW	TAGNSNTRFG	IAAKYQIDPD	ACFSAKVNNS	240
PASTA	HTNVNDGTEF	GGSIYQKVNK	KLETAVNLAW	TAGNSNTRFG	IAAKYQIDPD	ACFSAKVNNS	240
Amyl-pred	HTNVNDGTEF	GGSIYQKVNK	KLETAVNLAW	TAGNSNTRFG	IAAKYQIDPD	ACFSAKVNNS	240
Amyl-pred2	HTNVNDGTEF	GGSIYQKVNK	KLETAVNLAW	TAGNSNTRFG	IAAKYQIDPD	ACFSAKVNNS	240
GAP	HTNVNDGTEF	GGSIYQKVNK	KLETAVNLAW	TAGNSNTRFG	IAAKYQIDPD	ACFSAKVNNS	240
PaFig	HTNVNDGTEF	GGSIYQKVNK	KLETAVNLAW	TAGNSNTRFG	IAAKYQIDPD	ACFSAKVNNS	240
Amyl-mutants	HTNVNDGTEF	GGSIYQKVNK	KLETAVNLAW	TAGNSNTRFG	IAAKYQIDPD	ACFSAKVNNS	240
Amyl-patterns	HTNVNDGTEF	GGSIYQKVNK	KLETAVNLAW	TAGNSNTRFG	IAAKYQIDPD	ACFSAKVNNS	240
MetAmyl	HTNVNDGTEF	GGSIYQKVNK	KLETAVNLAW	TAGNSNTRFG	IAAKYQIDPD	ACFSAKVNNS	240
A.P.D.	HTNVNDGTEF	GGSIYQKVNK	KLETAVNLAW	TAGNSNTRFG	IAAKYQIDPD	ACFSAKVNNS	240
B.S.C.	HTNVNDGTEF	GGSIYQKVNK	KLETAVNLAW	TAGNSNTRFG	IAAKYQIDPD	ACFSAKVNNS	240
H.C.E.	HTNVNDGTEF	GGSIYQKVNK	KLETAVNLAW	TAGNSNTRFG	IAAKYQIDPD	ACFSAKVNNS	240
NetCSSP	HTNVNDGTEF	GGSIYQKVNK	KLETAVNLAW	TAGNSNTRFG	IAAKYQIDPD	ACFSAKVNNS	240
P.C.S.	HTNVNDGTEF	GGSIYQKVNK	KLETAVNLAW	TAGNSNTRFG	IAAKYQIDPD	ACFSAKVNNS	240
CamSol	HTNVNDGTEF	GGSIYQKVNK	KLETAVNLAW	TAGNSNTRFG	IAAKYQIDPD	ACFSAKVNNS	240
Consensus	HTNVNDGTEF	GGSIYQKVNK	KLETAVNLAW	TAGNSNTRFG	IAAKYQIDPD	ACFSAKVNNS	240
	**********	**********	**********	**********	**********	**********	

TANGO	SLIGLGYTQT	LKPGIKLTLS	ALLDGKNVNA	GGHKLGLGLE	FQA	283
Waltz	SLIGLGYTQT	LKPGIKLTLS	ALLDGKNVNA	GGHKLGLGLE	FQA	283
Aggrescan	SLIGLGYTQT	LKPGIKLTLS	ALLDGKNVNA	GGHKLGLGLE	FQA	283
Fish amyloid	SLIGLGYTQT	LKPGIKLTLS	ALLDGKNVNA	GGHKLGLGLE	FQA	283
Zyggregator	SLIGLGYTQT	LKPGIKLTLS	ALLDGKNVNA	GGHKLGLGLE	FQA	283
Fold amyloid	SLIGLGYTQT	LKPGIKLTLS	ALLDGKNVNA	GGHKLGLGLE	FQA	283
PASTA	SLIGLGYTQT	LKPGIKLTLS	ALLDGKNVNA	GGHKLGLGLE	FQA	283
Amyl-pred	SLIGLGYTQT	LKPGIKLTLS	ALLDGKNVNA	GGHKLGLGLE	FQA	283
Amyl-pred2	SLIGLGYTQT	LKPGIKLTLS	ALLDGKNVNA	GGHKLGLGLE	FQA	283
GAP	SLIGLGYTQT	LKPGIKLTLS	ALLDGKNVNA	GGHKLGLGLE	FQA	283
PaFig	SLIGLGYTQT	LKPGIKLTLS	ALLDGKNVNA	GGHKLGLGLE	FQA	283
Amyl-mutants	SLIGLGYTQT	LKPGIKLTLS	ALLDGKNVNA	GGHKLGLGLE	FQA	283
Amyl-patterns	SLIGLGYTQT	LKPGIKLTLS	ALLDGKNVNA	GGHKLGLGLE	FQA	283
MetAmyl	SLIGLGYTQT	LKPGIKLTLS	ALLDGKNVNA	GGHKLGLGLE	FQA	283
A.P.D.	SLIGLGYTQT	LKPGIKLTLS	ALLDGKNVNA	GGHKLGLGLE	FQA	283
B.S.C.	SLIGLGYTQT	LKPGIKLTLS	ALLDGKNVNA	GGHKLGLGLE	FQA	283
H.C.E	SLIGLGYTQT	LKPGIKLTLS	ALLDGKNVNA	GGHKLGLGLE	FQA	283
NetCSSP	SLIGLGYTQT	LKPGIKLTLS	ALLDGKNVNA	GGHKLGLGLE	FQA	283
P.C.S.	SLIGLGYTQT	LKPGIKLTLS	ALLDGKNVNA	GGHKLGLGLE	FQA	283
CamSol	SLIGLGYTQT	LKPGIKLTLS	ALLDGKNVNA	GGHKLGLGLE	FQA	283
Consensus	SLIGLGYTQT	LKPGIKLTLS	ALLDGKNVNA	GGHKLGLGLE	FQA	283
	**********	**********	**********	**********	***	

B

TANGO	MATHGQTCAR	PMCIPPSYAD	LGKAARDIFN	KGFGFGLVKL	DVKTKSCSGV	EFSTSGSSNT	60
Waltz	MATHGQTCAR	PMCIPPSYAD	LGKAARDIFN	KGFGFGLVKL	DVKTKSCSGV	EFSTSGSSNT	60
Aggrescan	MATHGQTCAR	PMCIPPSYAD	LGKAARDIFN	KGFGFGLVKL	DVKTKSCSGV	EFSTSGSSNT	60
Fish amyloid	MATHGQTCAR	PMCIPPSYAD	LGKAARDIFN	KGFGFGLVKL	DVKTKSCSGV	EFSTSGSSNT	60
Zyggregator	MATHGQTCAR	PMCIPPSYAD	LGKAARDIFN	KGFGFGLVKL	DVKTKSCSGV	EFSTSGSSNT	60
Fold amyloid	MATHGQTCAR	PMCIPPSYAD	LGKAARDIFN	KGFGFGLVKL	DVKTKSCSGV	EFSTSGSSNT	60
PASTA	MATHGQTCAR	PMCIPPSYAD	LGKAARDIFN	KGFGFGLVKL	DVKTKSCSGV	EFSTSGSSNT	60
Amyl-pred	MATHGQTCAR	PMCIPPSYAD	LGKAARDIFN	KGFGFGLVKL	DVKTKSCSGV	EFSTSGSSNT	60
Amyl-pred2	MATHGQTCAR	PMCIPPSYAD	LGKAARDIFN	KGFGFGLVKL	DVKTKSCSGV	EFSTSGSSNT	60
GAP	MATHGQTCAR	PMCIPPSYAD	LGKAARDIFN	KGFGFGLVKL	DVKTKSCSGV	EFSTSGSSNT	60
PaFig	MATHGQTCAR	PMCIPPSYAD	LGKAARDIFN	KGFGFGLVKL	DVKTKSCSGV	EFSTSGSSNT	60
Amyl-mutants	MATHGQTCAR	PMCIPPSYAD	LGKAARDIFN	KGFGFGLVKL	DVKTKSCSGV	EFSTSGSSNT	60
Amyl-patterns	MATHGQTCAR	PMCIPPSYAD	LGKAARDIFN	KGFGFGLVKL	DVKTKSCSGV	EFSTSGSSNT	60
MetAmyl	MATHGQTCAR	PMCIPPSYAD	LGKAARDIFN	KGFGFGLVKL	DVKTKSCSGV	EFSTSGSSNT	60
A.P.D.	MATHGQTCAR	PMCIPPSYAD	LGKAARDIFN	KGFGFGLVKL	DVKTKSCSGV	EFSTSGSSNT	60
B.S.C.	MATHGQTCAR	PMCIPPSYAD	LGKAARDIFN	KGFGFGLVKL	DVKTKSCSGV	EFSTSGSSNT	60
H.C.E	MATHGQTCAR	PMCIPPSYAD	LGKAARDIFN	KGFGFGLVKL	DVKTKSCSGV	EFSTSGSSNT	60
NetCSSP	MATHGQTCAR	PMCIPPSYAD	LGKAARDIFN	KGFGFGLVKL	DVKTKSCSGV	EFSTSGSSNT	60
P.C.S.	MATHGQTCAR	PMCIPPSYAD	LGKAARDIFN	KGFGFGLVKL	DVKTKSCSGV	EFSTSGSSNT	60
CamSol	MATHGQTCAR	PMCIPPSYAD	LGKAARDIFN	KGFGFGLVKL	DVKTKSCSGV	EFSTSGSSNT	60
Consensus	MATHGQTCAR	PMCIPPSYAD	LGKAARDIFN	KGFGFGLVKL	DVKTKSCSGV	EFSTSGSSNT	60
	**********	**********	**********	**********	**********	**********	

TANGO	DTGKVTGTLE	TKYKWCEYGL	TFTEKWNTDN	TLGTEIAIED	QICQGLKLTF	DTTFSPNTGK	120
Waltz	DTGKVTGTLE	TKYKWCEYGL	TFTEKWNTDN	TLGTEIAIED	QICQGLKLTF	DTTFSPNTGK	120
Aggrescan	DTGKVTGTLE	TKYKWCEYGL	TFTEKWNTDN	TLGTEIAIED	QICQGLKLTF	DTTFSPNTGK	120
Fish amyloid	DTGKVTGTLE	TKYKWCEYGL	TFTEKWNTDN	TLGTEIAIED	QICQGLKLTF	DTTFSPNTGK	120
Zyggregator	DTGKVTGTLE	TKYKWCEYGL	TFTEKWNTDN	TLGTEIAIED	QICQGLKLTF	DTTFSPNTGK	120
Fold amyloid	DTGKVTGTLE	TKYKWCEYGL	TFTEKWNTDN	TLGTEIAIED	QICQGLKLTF	DTTFSPNTGK	120
PASTA	DTGKVTGTLE	TKYKWCEYGL	TFTEKWNTDN	TLGTEIAIED	QICQGLKLTF	DTTFSPNTGK	120
Amyl-pred	DTGKVTGTLE	TKYKWCEYGL	TFTEKWNTDN	TLGTEIAIED	QICQGLKLTF	DTTFSPNTGK	120
Amyl-pred2	DTGKVTGTLE	TKYKWCEYGL	TFTEKWNTDN	TLGTEIAIED	QICQGLKLTF	DTTFSPNTGK	120
GAP	DTGKVTGTLE	TKYKWCEYGL	TFTEKWNTDN	TLGTEIAIED	QICQGLKLTF	DTTFSPNTGK	120
PaFig	DTGKVTGTLE	TKYKWCEYGL	TFTEKWNTDN	TLGTEIAIED	QICQGLKLTF	DTTFSPNTGK	120
Amyl-mutants	DTGKVTGTLE	TKYKWCEYGL	TFTEKWNTDN	TLGTEIAIED	QICQGLKLTF	DTTFSPNTGK	120
Amyl-patterns	DTGKVTGTLE	TKYKWCEYGL	TFTEKWNTDN	TLGTEIAIED	QICQGLKLTF	DTTFSPNTGK	120
MetAmyl	DTGKVTGTLE	TKYKWCEYGL	TFTEKWNTDN	TLGTEIAIED	QICQGLKLTF	DTTFSPNTGK	120
A.P.D.	DTGKVTGTLE	TKYKWCEYGL	TFTEKWNTDN	TLGTEIAIED	QICQGLKLTF	DTTFSPNTGK	120
B.S.C.	DTGKVTGTLE	TKYKWCEYGL	TFTEKWNTDN	TLGTEIAIED	QICQGLKLTF	DTTFSPNTGK	120
H.C.E	DTGKVTGTLE	TKYKWCEYGL	TFTEKWNTDN	TLGTEIAIED	QICQGLKLTF	DTTFSPNTGK	120
NetCSSP	DTGKVTGTLE	TKYKWCEYGL	TFTEKWNTDN	TLGTEIAIED	QICQGLKLTF	DTTFSPNTGK	120
P.C.S.	DTGKVTGTLE	TKYKWCEYGL	TFTEKWNTDN	TLGTEIAIED	QICQGLKLTF	DTTFSPNTGK	120
CamSol	DTGKVTGTLE	TKYKWCEYGL	TFTEKWNTDN	TLGTEIAIED	QICQGLKLTF	DTTFSPNTGK	120
Consensus	DTGKVTGTLE	TKYKWCEYGL	TFTEKWNTDN	TLGTEIAIED	QICQGLKLTF	DTTFSPNTGK	120
	**********	**********	**********	**********	**********	**********	

TANGO	KSGKIKSSYK	RECINLGCDV	DFDFAGPAIH	GSAVFGYEGW	LAGYQMTFDS	AKSKLTRNNF	180
Waltz	KSGKIKSSYK	RECINLGCDV	DFDFAGPAIH	GSAVFGYEGW	LAGYQMTFDS	AKSKLTRNNF	180
Aggrescan	KSGKIKSSYK	RECINLGCDV	DFDFAGPAIH	GSAVFGYEGW	LAGYQMTFDS	AKSKLTRNNF	180
Fish amyloid	KSGKIKSSYK	RECINLGCDV	DFDFAGPAIH	GSAVFGYEGW	LAGYQMTFDS	AKSKLTRNNF	180
Zyggregator	KSGKIKSSYK	RECINLGCDV	DFDFAGPAIH	GSAVFGYEGW	LAGYQMTFDS	AKSKLTRNNF	180
Fold amyloid	KSGKIKSSYK	RECINLGCDV	DFDFAGPAIH	GSAVFGYEGW	LAGYQMTFDS	AKSKLTRNNF	180
PASTA	KSGKIKSSYK	RECINLGCDV	DFDFAGPAIH	GSAVFGYEGW	LAGYQMTFDS	AKSKLTRNNF	180
Amyl-pred	KSGKIKSSYK	RECINLGCDV	DFDFAGPAIH	GSAVFGYEGW	LAGYQMTFDS	AKSKLTRNNF	180
Amyl-pred2	KSGKIKSSYK	RECINLGCDV	DFDFAGPAIH	GSAVFGYEGW	LAGYQMTFDS	AKSKLTRNNF	180
GAP	KSGKIKSSYK	RECINLGCDV	DFDFAGPAIH	GSAVFGYEGW	LAGYQMTFDS	AKSKLTRNNF	180
PaFig	KSGKIKSSYK	RECINLGCDV	DFDFAGPAIH	GSAVFGYEGW	LAGYQMTFDS	AKSKLTRNNF	180
Amyl-mutants	KSGKIKSSYK	RECINLGCDV	DFDFAGPAIH	GSAVFGYEGW	LAGYQMTFDS	AKSKLTRNNF	180
Amyl-patterns	KSGKIKSSYK	RECINLGCDV	DFDFAGPAIH	GSAVFGYEGW	LAGYQMTFDS	AKSKLTRNNF	180
MetAmyl	KSGKIKSSYK	RECINLGCDV	DFDFAGPAIH	GSAVFGYEGW	LAGYQMTFDS	AKSKLTRNNF	180
A.P.D.	KSGKIKSSYK	RECINLGCDV	DFDFAGPAIH	GSAVFGYEGW	LAGYQMTFDS	AKSKLTRNNF	180
B.S.C.	KSGKIKSSYK	RECINLGCDV	DFDFAGPAIH	GSAVFGYEGW	LAGYQMTFDS	AKSKLTRNNF	180
H.C.E	KSGKIKSSYK	RECINLGCDV	DFDFAGPAIH	GSAVFGYEGW	LAGYQMTFDS	AKSKLTRNNF	180
NetCSSP	KSGKIKSSYK	RECINLGCDV	DFDFAGPAIH	GSAVFGYEGW	LAGYQMTFDS	AKSKLTRNNF	180
P.C.S.	KSGKIKSSYK	RECINLGCDV	DFDFAGPAIH	GSAVFGYEGW	LAGYQMTFDS	AKSKLTRNNF	180
CamSol	KSGKIKSSYK	RECINLGCDV	DFDFAGPAIH	GSAVFGYEGW	LAGYQMTFDS	AKSKLTRNNF	180
Consensus	KSGKIKSSYK	RECINLGCDV	DFDFAGPAIH	GSAVFGYEGW	LAGYQMTFDS	AKSKLTRNNF	180
	**********	**********	**********	**********	**********	**********	

TANGO	AVGYRTGDFQ	LHTNVNDGTE	FGGSIYQKVC	EDLDTSVNLA	WTSGTNCTRF	GIAAKYQLDP	240
Waltz	AVGYRTGDFQ	LHTNVNDGTE	FGGSIYQKVC	EDLDTSVNLA	WTSGTNCTRF	GIAAKYQLDP	240
Aggrescan	AVGYRTGDFQ	LHTNVNDGTE	FGGSIYQKVC	EDLDTSVNLA	WTSGTNCTRF	GIAAKYQLDP	240
Fish amyloid	AVGYRTGDFQ	LHTNVNDGTE	FGGSIYQKVC	EDLDTSVNLA	WTSGTNCTRF	GIAAKYQLDP	240
Zyggregator	AVGYRTGDFQ	LHTNVNDGTE	FGGSIYQKVC	EDLDTSVNLA	WTSGTNCTRF	GIAAKYQLDP	240
Fold amyloid	AVGYRTGDFQ	LHTNVNDGTE	FGGSIYQKVC	EDLDTSVNLA	WTSGTNCTRF	GIAAKYQLDP	240
PASTA	AVGYRTGDFQ	LHTNVNDGTE	FGGSIYQKVC	EDLDTSVNLA	WTSGTNCTRF	GIAAKYQLDP	240
Amyl-pred	AVGYRTGDFQ	LHTNVNDGTE	FGGSIYQKVC	EDLDTSVNLA	WTSGTNCTRF	GIAAKYQLDP	240
Amyl-pred2	AVGYRTGDFQ	LHTNVNDGTE	FGGSIYQKVC	EDLDTSVNLA	WTSGTNCTRF	GIAAKYQLDP	240
GAP	AVGYRTGDFQ	LHTNVNDGTE	FGGSIYQKVC	EDLDTSVNLA	WTSGTNCTRF	GIAAKYQLDP	240
PaFig	AVGYRTGDFQ	LHTNVNDGTE	FGGSIYQKVC	EDLDTSVNLA	WTSGTNCTRF	GIAAKYQLDP	240
Amyl-mutants	AVGYRTGDFQ	LHTNVNDGTE	FGGSIYQKVC	EDLDTSVNLA	WTSGTNCTRF	GIAAKYQLDP	240
Amyl-patterns	AVGYRTGDFQ	LHTNVNDGTE	FGGSIYQKVC	EDLDTSVNLA	WTSGTNCTRF	GIAAKYQLDP	240
MetAmyl	AVGYRTGDFQ	LHTNVNDGTE	FGGSIYQKVC	EDLDTSVNLA	WTSGTNCTRF	GIAAKYQLDP	240
A.P.D.	AVGYRTGDFQ	LHTNVNDGTE	FGGSIYQKVC	EDLDTSVNLA	WTSGTNCTRF	GIAAKYQLDP	240
B.S.C.	AVGYRTGDFQ	LHTNVNDGTE	FGGSIYQKVC	EDLDTSVNLA	WTSGTNCTRF	GIAAKYQLDP	240
H.C.E.	AVGYRTGDFQ	LHTNVNDGTE	FGGSIYQKVC	EDLDTSVNLA	WTSGTNCTRF	GIAAKYQLDP	240
NetCSSP	AVGYRTGDFQ	LHTNVNDGTE	FGGSIYQKVC	EDLDTSVNLA	WTSGTNCTRF	GIAAKYQLDP	240
P.C.S.	AVGYRTGDFQ	LHTNVNDGTE	FGGSIYQKVC	EDLDTSVNLA	WTSGTNCTRF	GIAAKYQLDP	240
CamSol	AVGYRTGDFQ	LHTNVNDGTE	FGGSIYQKVC	EDLDTSVNLA	WTSGTNCTRF	GIAAKYQLDP	240
Consensus	AVGYRTGDFQ	LHTNVNDGTE	FGGSIYQKVC	EDLDTSVNLA	WTSGTNCTRF	GIAAKYQLDP	240
	**********	**********	**********	**********	**********	**********	

TANGO	TASISAKVNN	SSLIGVGYTQ	TLRPGVKLTL	SALVDGKSIN	AGGHKVGLAL	ELEA	294
Waltz	TASISAKVNN	SSLIGVGYTQ	TLRPGVKLTL	SALVDGKSIN	AGGHKVGLAL	ELEA	294
Aggrescan	TASISAKVNN	SSLIGVGYTQ	TLRPGVKLTL	SALVDGKSIN	AGGHKVGLAL	ELEA	294
Fish amyloid	TASISAKVNN	SSLIGVGYTQ	TLRPGVKLTL	SALVDGKSIN	AGGHKVGLAL	ELEA	294
Zyggregator	TASISAKVNN	SSLIGVGYTQ	TLRPGVKLTL	SALVDGKSIN	AGGHKVGLAL	ELEA	294
Fold amyloid	TASISAKVNN	SSLIGVGYTQ	TLRPGVKLTL	SALVDGKSIN	AGGHKVGLAL	ELEA	294
PASTA	TASISAKVNN	SSLIGVGYTQ	TLRPGVKLTL	SALVDGKSIN	AGGHKVGLAL	ELEA	294
Amyl-pred	TASISAKVNN	SSLIGVGYTQ	TLRPGVKLTL	SALVDGKSIN	AGGHKVGLAL	ELEA	294
Amyl-pred2	TASISAKVNN	SSLIGVGYTQ	TLRPGVKLTL	SALVDGKSIN	AGGHKVGLAL	ELEA	294
GAP	TASISAKVNN	SSLIGVGYTQ	TLRPGVKLTL	SALVDGKSIN	AGGHKVGLAL	ELEA	294
PaFig	TASISAKVNN	SSLIGVGYTQ	TLRPGVKLTL	SALVDGKSIN	AGGHKVGLAL	ELEA	294
Amyl-mutants	TASISAKVNN	SSLIGVGYTQ	TLRPGVKLTL	SALVDGKSIN	AGGHKVGLAL	ELEA	294
Amyl-patterns	TASISAKVNN	SSLIGVGYTQ	TLRPGVKLTL	SALVDGKSIN	AGGHKVGLAL	ELEA	294
MetAmyl	TASISAKVNN	SSLIGVGYTQ	TLRPGVKLTL	SALVDGKSIN	AGGHKVGLAL	ELEA	294
A.P.D.	TASISAKVNN	SSLIGVGYTQ	TLRPGVKLTL	SALVDGKSIN	AGGHKVGLAL	ELEA	294
B.S.C.	TASISAKVNN	SSLIGVGYTQ	TLRPGVKLTL	SALVDGKSIN	AGGHKVGLAL	ELEA	294
H.C.E	TASISAKVNN	SSLIGVGYTQ	TLRPGVKLTL	SALVDGKSIN	AGGHKVGLAL	ELEA	294
NetCSSP	TASISAKVNN	SSLIGVGYTQ	TLRPGVKLTL	SALVDGKSIN	AGGHKVGLAL	ELEA	294
P.C.S.	TASISAKVNN	SSLIGVGYTQ	TLRPGVKLTL	SALVDGKSIN	AGGHKVGLAL	ELEA	294
CamSol	TASISAKVNN	SSLIGVGYTQ	TLRPGVKLTL	SALVDGKSIN	AGGHKVGLAL	ELEA	294
Consensus	TASISAKVNN	SSLIGVGYTQ	TLRPGVKLTL	SALVDGKSIN	AGGHKVGLAL	ELEA	294
	**********	**********	**********	**********	**********	****	

C

TANGO	MCNTPTYCDL	GKAAKDVFNK	GYGFGMVKID	LKTKSCSGVE	FSTSGHAYTD	TGKASGNLET	60
Waltz	MCNTPTYCDL	GKAAKDVFNK	GYGFGMVKID	LKTKSCSGVE	FSTSGHAYTD	TGKASGNLET	60
Aggrescan	MCNTPTYCDL	GKAAKDVFNK	GYGFGMVKID	LKTKSCSGVE	FSTSGHAYTD	TGKASGNLET	60
FISH amyloid	MCNTPTYCDL	GKAAKDVFNK	GYGFGMVKID	LKTKSCSGVE	FSTSGHAYTD	TGKASGNLET	60
Zyggregator	MCNTPTYCDL	GKAAKDVFNK	GYGFGMVKID	LKTKSCSGVE	FSTSGHAYTD	TGKASGNLET	60
Fold amyloid	MCNTPTYCDL	GKAAKDVFNK	GYGFGMVKID	LKTKSCSGVE	FSTSGHAYTD	TGKASGNLET	60
PASTA	MCNTPTYCDL	GKAAKDVFNK	GYGFGMVKID	LKTKSCSGVE	FSTSGHAYTD	TGKASGNLET	60
Amyl-pred	MCNTPTYCDL	GKAAKDVFNK	GYGFGMVKID	LKTKSCSGVE	FSTSGHAYTD	TGKASGNLET	60
Amyl-pred2	MCNTPTYCDL	GKAAKDVFNK	GYGFGMVKID	LKTKSCSGVE	FSTSGHAYTD	TGKASGNLET	60
GAP	MCNTPTYCDL	GKAAKDVFNK	GYGFGMVKID	LKTKSCSGVE	FSTSGHAYTD	TGKASGNLET	60
Pafig	MCNTPTYCDL	GKAAKDVFNK	GYGFGMVKID	LKTKSCSGVE	FSTSGHAYTD	TGKASGNLET	60
Amyl-mutants	MCNTPTYCDL	GKAAKDVFNK	GYGFGMVKID	LKTKSCSGVE	FSTSGHAYTD	TGKASGNLET	60
Amyl-patterns	MCNTPTYCDL	GKAAKDVFNK	GYGFGMVKID	LKTKSCSGVE	FSTSGHAYTD	TGKASGNLET	60
MetAmyl	MCNTPTYCDL	GKAAKDVFNK	GYGFGMVKID	LKTKSCSGVE	FSTSGHAYTD	TGKASGNLET	60
A.P.D.	MCNTPTYCDL	GKAAKDVFNK	GYGFGMVKID	LKTKSCSGVE	FSTSGHAYTD	TGKASGNLET	60
B.S.C.	MCNTPTYCDL	GKAAKDVFNK	GYGFGMVKID	LKTKSCSGVE	FSTSGHAYTD	TGKASGNLET	60
H.C.E.	MCNTPTYCDL	GKAAKDVFNK	GYGFGMVKID	LKTKSCSGVE	FSTSGHAYTD	TGKASGNLET	60
NetCSSP	MCNTPTYCDL	GKAAKDVFNK	GYGFGMVKID	LKTKSCSGVE	FSTSGHAYTD	TGKASGNLET	60
P.C.S.	MCNTPTYCDL	GKAAKDVFNK	GYGFGMVKID	LKTKSCSGVE	FSTSGHAYTD	TGKASGNLET	60
CamSol	MCNTPTYCDL	GKAAKDVFNK	GYGFGMVKID	LKTKSCSGVE	FSTSGHAYTD	TGKASGNLET	60
Consensus	MCNTPTYCDL	GKAAKDVFNK	GYGFGMVKID	LKTKSCSGVE	FSTSGHAYTD	TGKASGNLET	60
	**********	**********	**********	**********	**********	**********	

TANGO	KYKVCNYGLT	FTQKWNTDNT	LGTEISWENK	LAEGLKLTLD	TIFVPNTGKK	SGKLKASYKR	120
Waltz	KYKVCNYGLT	FTQKWNTDNT	LGTEISWENK	LAEGLKLTLD	TIFVPNTGKK	SGKLKASYKR	120
Aggrescan	KYKVCNYGLT	FTQKWNTDNT	LGTEISWENK	LAEGLKLTLD	TIFVPNTGKK	SGKLKASYKR	120
FISH amyloid	KYKVCNYGLT	FTQKWNTDNT	LGTEISWENK	LAEGLKLTLD	TIFVPNTGKK	SGKLKASYKR	120
Zyggregator	KYKVCNYGLT	FTQKWNTDNT	LGTEISWENK	LAEGLKLTLD	TIFVPNTGKK	SGKLKASYKR	120
Fold amyloid	KYKVCNYGLT	FTQKWNTDNT	LGTEISWENK	LAEGLKLTLD	TIFVPNTGKK	SGKLKASYKR	120
PASTA	KYKVCNYGLT	FTQKWNTDNT	LGTEISWENK	LAEGLKLTLD	TIFVPNTGKK	SGKLKASYKR	120
Amyl-pred	KYKVCNYGLT	FTQKWNTDNT	LGTEISWENK	LAEGLKLTLD	TIFVPNTGKK	SGKLKASYKR	120
Amyl-pred2	KYKVCNYGLT	FTQKWNTDNT	LGTEISWENK	LAEGLKLTLD	TIFVPNTGKK	SGKLKASYKR	120
GAP	KYKVCNYGLT	FTQKWNTDNT	LGTEISWENK	LAEGLKLTLD	TIFVPNTGKK	SGKLKASYKR	120
Pafig	KYKVCNYGLT	FTQKWNTDNT	LGTEISWENK	LAEGLKLTLD	TIFVPNTGKK	SGKLKASYKR	120
Amyl-mutants	KYKVCNYGLT	FTQKWNTDNT	LGTEISWENK	LAEGLKLTLD	TIFVPNTGKK	SGKLKASYKR	120
Amyl-patterns	KYKVCNYGLT	FTQKWNTDNT	LGTEISWENK	LAEGLKLTLD	TIFVPNTGKK	SGKLKASYKR	120
MetAmyl	KYKVCNYGLT	FTQKWNTDNT	LGTEISWENK	LAEGLKLTLD	TIFVPNTGKK	SGKLKASYKR	120
A.P.D.	KYKVCNYGLT	FTQKWNTDNT	LGTEISWENK	LAEGLKLTLD	TIFVPNTGKK	SGKLKASYKR	120
B.S.C.	KYKVCNYGLT	FTQKWNTDNT	LGTEISWENK	LAEGLKLTLD	TIFVPNTGKK	SGKLKASYKR	120
H.C.E.	KYKVCNYGLT	FTQKWNTDNT	LGTEISWENK	LAEGLKLTLD	TIFVPNTGKK	SGKLKASYKR	120
NetCSSP	KYKVCNYGLT	FTQKWNTDNT	LGTEISWENK	LAEGLKLTLD	TIFVPNTGKK	SGKLKASYKR	120
P.C.S.	KYKVCNYGLT	FTQKWNTDNT	LGTEISWENK	LAEGLKLTLD	TIFVPNTGKK	SGKLKASYKR	120
CamSol	KYKVCNYGLT	FTQKWNTDNT	LGTEISWENK	LAEGLKLTLD	TIFVPNTGKK	SGKLKASYKR	120
Consensus	KYKVCNYGLT	FTQKWNTDNT	LGTEISWENK	LAEGLKLTLD	TIFVPNTGKK	SGKLKASYKR	120
	**********	**********	**********	**********	**********	**********	
TANGO	DCFSVGSNVD	IDFSGPTIYG	WAVLAFEGWL	AGYQMSFDTA	KSKLSQNNFA	LGYKAADFQL	180

Waltz	DCFSVGSNVD	IDFSGPTIYG	WAVLAFEGWL	AGYQMSFDTA	KSKLSQNNFA	LGYKAADFQL	180
Aggrescan	DCFSVGSNVD	IDFSGPTIYG	WAVLAFEGWL	AGYQMSFDTA	KSKLSQNNFA	LGYKAADFQL	180
FISH amyloid	DCFSVGSNVD	IDFSGPTIYG	WAVLAFEGWL	AGYQMSFDTA	KSKLSQNNFA	LGYKAADFQL	180
Zyggregator	DCFSVGSNVD	IDFSGPTIYG	WAVLAFEGWL	AGYQMSFDTA	KSKLSQNNFA	LGYKAADFQL	180
Fold amyloid	DCFSVGSNVD	IDFSGPTIYG	WAVLAFEGWL	AGYQMSFDTA	KSKLSQNNFA	LGYKAADFQL	180
PASTA	DCFSVGSNVD	IDFSGPTIYG	WAVLAFEGWL	AGYQMSFDTA	KSKLSQNNFA	LGYKAADFQL	180
Amyl-pred	DCFSVGSNVD	IDFSGPTIYG	WAVLAFEGWL	AGYQMSFDTA	KSKLSQNNFA	LGYKAADFQL	180
Amyl-pred2	DCFSVGSNVD	IDFSGPTIYG	WAVLAFEGWL	AGYQMSFDTA	KSKLSQNNFA	LGYKAADFQL	180
GAP	DCFSVGSNVD	IDFSGPTIYG	WAVLAFEGWL	AGYQMSFDTA	KSKLSQNNFA	LGYKAADFQL	180
Pafig	DCFSVGSNVD	IDFSGPTIYG	WAVLAFEGWL	AGYQMSFDTA	KSKLSQNNFA	LGYKAADFQL	180
Amyl-mutants	DCFSVGSNVD	IDFSGPTIYG	WAVLAFEGWL	AGYQMSFDTA	KSKLSQNNFA	LGYKAADFQL	180
Amyl-patterns	DCFSVGSNVD	IDFSGPTIYG	WAVLAFEGWL	AGYQMSFDTA	KSKLSQNNFA	LGYKAADFQL	180
MetAmyl	DCFSVGSNVD	IDFSGPTIYG	WAVLAFEGWL	AGYQMSFDTA	KSKLSQNNFA	LGYKAADFQL	180
A.P.D.	DCFSVGSNVD	IDFSGPTIYG	WAVLAFEGWL	AGYQMSFDTA	KSKLSQNNFA	LGYKAADFQL	180
B.S.C.	DCFSVGSNVD	IDFSGPTIYG	WAVLAFEGWL	AGYQMSFDTA	KSKLSQNNFA	LGYKAADFQL	180
H.C.E.	DCFSVGSNVD	IDFSGPTIYG	WAVLAFEGWL	AGYQMSFDTA	KSKLSQNNFA	LGYKAADFQL	180
NetCSSP	DCFSVGSNVD	IDFSGPTIYG	WAVLAFEGWL	AGYQMSFDTA	KSKLSQNNFA	LGYKAADFQL	180
P.C.S.	DCFSVGSNVD	IDFSGPTIYG	WAVLAFEGWL	AGYQMSFDTA	KSKLSQNNFA	LGYKAADFQL	180
CamSol	DCFSVGSNVD	IDFSGPTIYG	WAVLAFEGWL	AGYQMSFDTA	KSKLSQNNFA	LGYKAADFQL	180
Consensus	DCFSVGSNVD	IDFSGPTIYG	WAVLAFEGWL	AGYQMSFDTA	KSKLSQNNFA	LGYKAADFQL	180
	**********	**********	**********	**********	**********	**********	

TANGO	HTHVNDGTEF	GGSIYQKVNE	KIETSINLAW	TAGSNNTRFG	IAAKYMLDCR	TSLSAKVNNA	240
Waltz	HTHVNDGTEF	GGSIYQKVNE	KIETSINLAW	TAGSNNTRFG	IAAKYMLDCR	TSLSAKVNNA	240
Aggrescan	HTHVNDGTEF	GGSIYQKVNE	KIETSINLAW	TAGSNNTRFG	IAAKYMLDCR	TSLSAKVNNA	240
Fish amyloid	HTHVNDGTEF	GGSIYQKVNE	KIETSINLAW	TAGSNNTRFG	IAAKYMLDCR	TSLSAKVNNA	240
Zyggregator	HTHVNDGTEF	GGSIYQKVNE	KIETSINLAW	TAGSNNTRFG	IAAKYMLDCR	TSLSAKVNNA	240
Fold amyloid	HTHVNDGTEF	GGSIYQKVNE	KIETSINLAW	TAGSNNTRFG	IAAKYMLDCR	TSLSAKVNNA	240
PASTA	HTHVNDGTEF	GGSIYQKVNE	KIETSINLAW	TAGSNNTRFG	IAAKYMLDCR	TSLSAKVNNA	240
Amyl-pred	HTHVNDGTEF	GGSIYQKVNE	KIETSINLAW	TAGSNNTRFG	IAAKYMLDCR	TSLSAKVNNA	240
Amyl-pred2	HTHVNDGTEF	GGSIYQKVNE	KIETSINLAW	TAGSNNTRFG	IAAKYMLDCR	TSLSAKVNNA	240
GAP	HTHVNDGTEF	GGSIYQKVNE	KIETSINLAW	TAGSNNTRFG	IAAKYMLDCR	TSLSAKVNNA	240
Pafig	HTHVNDGTEF	GGSIYQKVNE	KIETSINLAW	TAGSNNTRFG	IAAKYMLDCR	TSLSAKVNNA	240
Amyl-mutants	HTHVNDGTEF	GGSIYQKVNE	KIETSINLAW	TAGSNNTRFG	IAAKYMLDCR	TSLSAKVNNA	240
Amyl-patterns	HTHVNDGTEF	GGSIYQKVNE	KIETSINLAW	TAGSNNTRFG	IAAKYMLDCR	TSLSAKVNNA	240
MetAmyl	HTHVNDGTEF	GGSIYQKVNE	KIETSINLAW	TAGSNNTRFG	IAAKYMLDCR	TSLSAKVNNA	240
A.P.D.	HTHVNDGTEF	GGSIYQKVNE	KIETSINLAW	TAGSNNTRFG	IAAKYMLDCR	TSLSAKVNNA	240
B.S.C.	HTHVNDGTEF	GGSIYQKVNE	KIETSINLAW	TAGSNNTRFG	IAAKYMLDCR	TSLSAKVNNA	240
H.C.E.	HTHVNDGTEF	GGSIYQKVNE	KIETSINLAW	TAGSNNTRFG	IAAKYMLDCR	TSLSAKVNNA	240
NetCSSP	HTHVNDGTEF	GGSIYQKVNE	KIETSINLAW	TAGSNNTRFG	IAAKYMLDCR	TSLSAKVNNA	240
P.C.S.	HTHVNDGTEF	GGSIYQKVNE	KIETSINLAW	TAGSNNTRFG	IAAKYMLDCR	TSLSAKVNNA	240
CamSol	HTHVNDGTEF	GGSIYQKVNE	KIETSINLAW	TAGSNNTRFG	IAAKYMLDCR	TSLSAKVNNA	240
Consensus	HTHVNDGTEF	GGSIYQKVNE	KIETSINLAW	TAGSNNTRFG	IAAKYMLDCR	TSLSAKVNNA	240
	**********	**********	**********	**********	**********	**********	

TANGO	SLIGLGYTQT	LRPGVKLTLS	ALIDGKNFSA	GGHKVGLGFE	LEALE	285
Waltz	SLIGLGYTQT	LRPGVKLTLS	ALIDGKNFSA	GGHKVGLGFE	LEALE	285
Aggrescan	SLIGLGYTQT	LRPGVKLTLS	ALIDGKNFSA	GGHKVGLGFE	LEALE	285
Fish amyloid	SLIGLGYTQT	LRPGVKLTLS	ALIDGKNFSA	GGHKVGLGFE	LEALE	285
Zyggregator	SLIGLGYTQT	LRPGVKLTLS	ALIDGKNFSA	GGHKVGLGFE	LEALE	285
Fold amyloid	SLIGLGYTQT	LRPGVKLTLS	ALIDGKNFSA	GGHKVGLGFE	LEALE	285
PASTA	SLIGLGYTQT	LRPGVKLTLS	ALIDGKNFSA	GGHKVGLGFE	LEALE	285
Amyl-pred	SLIGLGYTQT	LRPGVKLTLS	ALIDGKNFSA	GGHKVGLGFE	LEALE	285
Amyl-pred2	SLIGLGYTQT	LRPGVKLTLS	ALIDGKNFSA	GGHKVGLGFE	LEALE	285
GAP	SLIGLGYTQT	LRPGVKLTLS	ALIDGKNFSA	GGHKVGLGFE	LEALE	285
Pafig	SLIGLGYTQT	LRPGVKLTLS	ALIDGKNFSA	GGHKVGLGFE	LEALE	285
Amyl-mutants	SLIGLGYTQT	LRPGVKLTLS	ALIDGKNFSA	GGHKVGLGFE	LEALE	285
Amyl-patterns	SLIGLGYTQT	LRPGVKLTLS	ALIDGKNFSA	GGHKVGLGFE	LEALE	285
MetAmyl	SLIGLGYTQT	LRPGVKLTLS	ALIDGKNFSA	GGHKVGLGFE	LEALE	285
A.P.D.	SLIGLGYTQT	LRPGVKLTLS	ALIDGKNFSA	GGHKVGLGFE	LEALE	285
B.S.C.	SLIGLGYTQT	LRPGVKLTLS	ALIDGKNFSA	GGHKVGLGFE	LEALE	285
H.C.E.	SLIGLGYTQT	LRPGVKLTLS	ALIDGKNFSA	GGHKVGLGFE	LEALE	285
NetCSSP	SLIGLGYTQT	LRPGVKLTLS	ALIDGKNFSA	GGHKVGLGFE	LEALE	285
P.C.S.	SLIGLGYTQT	LRPGVKLTLS	ALIDGKNFSA	GGHKVGLGFE	LEALE	285
CamSol	SLIGLGYTQT	LRPGVKLTLS	ALIDGKNFSA	GGHKVGLGFE	LEALE	285
Consensus	SLIGLGYTQT	LRPGVKLTLS	ALIDGKNFSA	GGHKVGLGFE	LEALE	285
	**********	**********	**********	**********	*****	
